# Emerging Concepts in Immuno-Oncology: Insights from Natural Language Processing-Driven Co-Occurrence Analysis

**DOI:** 10.1021/acsomega.5c00693

**Published:** 2025-06-27

**Authors:** Kavita A Iyer, Rumiana Tenchov, Julian M Ivanov, Qiongqiong Angela Zhou

**Affiliations:** 2645 CAS, A Division of the American Chemical Society, Columbus, Ohio 43210, United States

## Abstract

Immuno-oncology, a rapidly evolving field at the forefront of cancer research, leverages the body’s immune system to fight cancer. In this follow-up report, we extend our natural language processing-based analysis to pinpoint the context of emergence of the previously identified emerging concepts. To achieve this relational analysis, we devised a method to identify terms co-occurring in a single sentence in the title or abstract of >350,000 journal and patent publications. The focus of the current study was on intergroup co-occurrences, namely between therapeutic targets, biomarkers, types of therapy, and types of cancer. In our discussion, we have highlighted potentially emerging co-occurring concept pairs between each of the groups exhibiting rapid growth in publications over the past few years but an overall small number of publications. This analysis provides contextual information that may be useful in directing future research efforts in a valuable direction.

## Graphical abstract

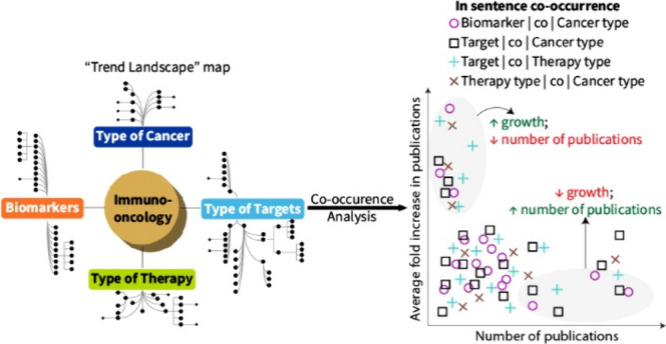

## Introduction

Immuno-oncology, a rapidly advancing field within medical science, represents a paradigm shift in cancer treatment.^1–3^ Leveraging the body’s own immune system to recognize and combat cancer cells, immunotherapy offers a promising alternative to traditional therapies such as chemotherapy and radiation. This approach includes a range of strategies such as immune checkpoint inhibitors (ICIs), chimeric antigenic receptor T-cell therapy (CAR-T), and cancer vaccines,^4^ each designed to enhance the immune system’s natural ability to fight cancer.^5–7^

The growing interest and investment in immuno-oncology are driven by its potential to provide more targeted and effective treatments with fewer side effects compared with conventional methods. As researchers uncover more about the complex interactions between the immune system and cancer, new therapies are continually being developed and refined. The field’s dynamic nature promises not only improved survival rates for cancer patients but also the possibility of long-term remission and even cures for some types of cancer.

We recently presented our findings using a natural language programming (NLP)-based methodology for analyzing publications related to immuno-oncology sourced from the CAS Content Collection,^8^ the largest human-curated repository of scientific publications, and developed CAS TrendScape maps showcasing major concepts in immuno-oncology.^1^ These maps provide a wealth of information in a condensed and easy-to-read format by clustering similar concepts together.^9^

Now, in this follow-up report, we extend our analysis further by examining the co-occurrences of concepts in the literature; we can identify promising intersections of concepts that suggest where the next breakthrough is likely to happen. This approach drives innovation forward by helping researchers focus on emerging ideas and accelerate their next discovery faster.

## Methods

The results and detailed methodology of the NLP analysis have been published previously.^1^ Briefly, a search query was created by subject matter experts to cover the field of immuno-oncology using multiple keywords, including synonyms and pertinent abbreviations. Data for the results of the search query were extracted and included titles and abstracts. Using NLP, we identified candidate phrases from the titles and abstracts ranging between 1 and 6 words in length and eventually led to the identification of 338,054 candidate phrases that were analyzed by subject matter experts. Identified phrases were condensed into groups and subgroups and arranged in a hierarchical manner to create the CAS TrendScape map.^1^

To overcome the lack of context in which topics are emerging, we performed a co-occurrence analysis to look for pairs of emerging topics. For a detailed description of the co-occurrence analysis methodology, please see the Supporting Information. Briefly, we determined two types of co-occurrences: ones occurring in sentences and in abstracts, with each pair of topics consisting of multiple keywords, including synonyms. We computed a number of metrics for co-occurring pairs and treated each pair of co-occurring topics as a new scientific topic.

## Results and Discussion

Co-occurrence analysis in bibliometrics aims at investigating counts of co-occurring terms within a collection of textual units and is used to study the relationships between bibliometric items that appear in the same unit, such as a single sentence or a document. Statistical measures of co-occurrence strength help identify meaningful multiword expressions and collocations that function as lexical units. For a discussion on traditional statistical approaches, challenges associated with them, and greater details about co-occurrence analysis, please see the Supporting Information.

To understand connections between concepts in the immuno-oncology CAS TrendScape map, we performed an NLP-based analysis, which counts the number of co-occurrences of individual emerging concepts in the same sentences of abstracts. This allows us to quantify the degree and strength of connection between any two concepts shown in our published CAS TrendScape maps of emerging concepts in immuno-oncology.^1^

The plot in Figure 1 illustrates the co-occurrences of emerging concepts in immuno-oncology across four major concept pairs—biomarkers and cancer type, therapeutic targets and cancer type, therapeutic targets and therapy types, and therapy and cancer types. Concept pairs co-occurred within a sentence in the title or abstract of publications. Plotted are the number of documents published between 2019 and 2022 where pairs of terms co-occur in the same sentence (*x*-axis) and the average growth rate of documents with those co-occurrences over the same time period (*y*-axis). At the outset, the general trend that emerged was that there is a wide range of growth rates for the combinations with relatively low publication frequency (less than 20–40 documents per year), with a long tail extending to high publication numbers but relatively low growth rates.

**Figure 1:**
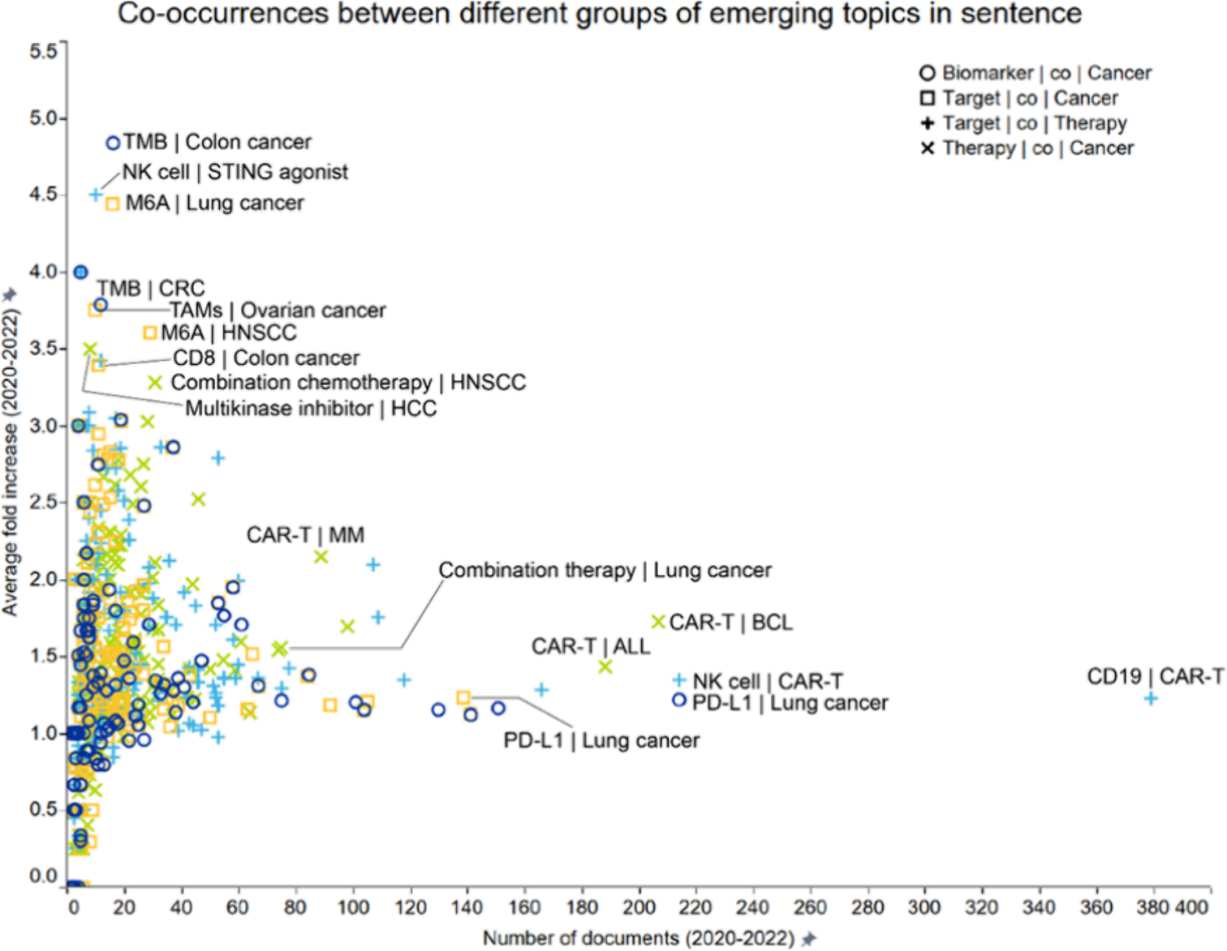
Co-occurrence analysis of emerging concepts in immuno-oncology across four major concept pairs—biomarkers and cancer types (circle open), therapeutic targets and cancer types (box open), therapeutic targets and therapy types (plus sign), and therapy and cancer types (multiplication sign). Concepts pairs co-occurred within a sentence in the title or abstract of publications. Labels outline certain noteworthy combinations exhibiting either a high number of documents (right part of the graph) or high growth rate (upper left).

### In-Sentence Co-Occurrences: Therapeutic Targets and Cancer Types

The identification of targets in cancer treatment has become a cornerstone of precision oncology, with the aim of developing therapies that specifically target cancer cells while sparing normal tissues. The concept of targets in cancer refers to protein molecules or pathways that play crucial roles in the growth and survival of cancer cells. By understanding and exploiting these targets, researchers and clinicians can design treatments that are more effective and have fewer side effects than traditional therapies. The co-occurrence of targets and cancer concepts refers to the interplay between various molecular targets and the characteristics of different cancers. Understanding these relationships is crucial for developing targeted therapies and improving patient outcomes.

Figure 2 illustrates our results from the co-occurrence analysis of emerging concepts in immuno-oncology focused on various types of therapeutic targets and cancer types. Highlighted below are a few interesting co-occurring pairs, many of which involve N6-methyladenosine (m^6^A). We have chosen candidates from both ends of the spectrum: those with high average fold increase but relatively low number of publications (m^6^A | lung cancer, m^6^A | liver cancer, TAMs | ovarian cancer, CD8 | colon cancer, PD-1 | colorectal cancer, and HER2 | NSCLC) indicative of recent growing interest (and hence emergence), as well as concept pairs with relatively lower average fold increase but with a large number of publications (PD-1 | liver cancer, PD-L1 | gastric cancer). Figure 3 shows yearly trends for these therapeutic targets and cancer type co-occurring pairs. All the chosen pairs show an increase in publication after 2019, with the PD1 | liver cancer pair showing a 4-fold increase between 2019 and 2020.

**Figure 2:**
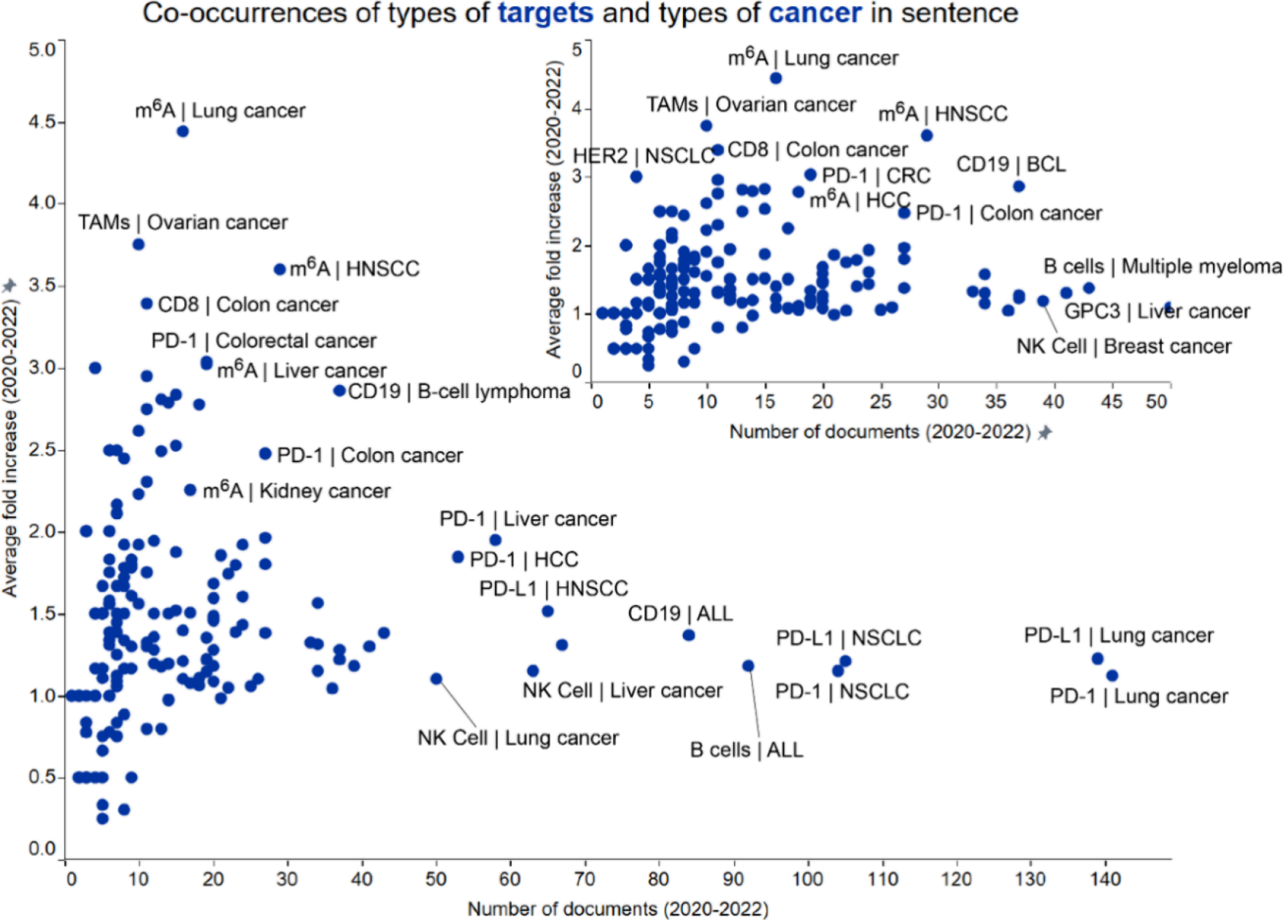
Co-occurrence analysis of emerging concepts in immuno-oncology focused on therapeutic targets and cancer types. Concepts pairs co-occurred within a sentence in the title or abstract of publications. Labels outline certain noteworthy combinations exhibiting either high number of documents (right part of the graph) or high growth rate (upper left). Shown in the inset is a graph focused on data clustered in the lower left corner of the bigger graph.

**Figure 3:**
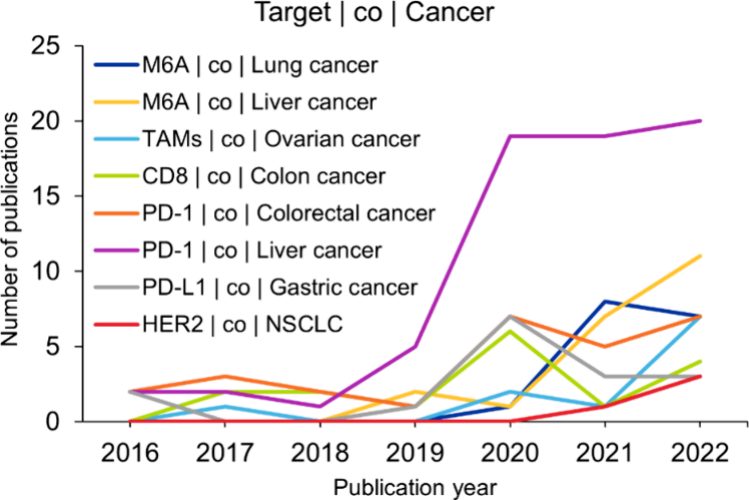
Time trends of a few chosen concept pairs from the category’s therapeutic targets and cancer types. Data include journal and patent publications related to immuno-oncology from the CAS Content Collection for the period 2016–2022.

N6-Methyladenosine (m^6^A) is a type of reversible RNA modification that is achieved using a combination of “writers”, “readers”, and “erasers”.^10^ The dysregulation of m^6^A may lead to tumorigenesis and progression, making m^6^A regulators potential clinical therapeutic targets for cancers.^11,12^

#### m^6^A | Lung cancer

A prevalent and dynamic RNA modification, N6-methyladenosine (m^6^A), plays a crucial role in various biological processes, including mRNA stability, splicing, translation, and degradation. Recent research has highlighted the significance of m^6^A modification in cancer biology, including lung cancer, where it influences tumor development, progression, and response to therapy. Emerging studies have demonstrated that dysregulation of m^6^A is involved in many diseases, especially pulmonary tumorigenesis and progression.^13–19^

m^6^A modifications can contribute to drug resistance in lung cancer. For example, alterations in m^6^A levels can affect the sensitivity of lung cancer cells to chemotherapeutic agents or targeted therapies, such as epidermal growth factor receptor (EGFR) inhibitors. Understanding these mechanisms may help in developing strategies to overcome resistance.^20,21^

m^6^A modifications can influence the tumor immune microenvironment by regulating the expression of immune checkpoint molecules and cytokines. This can impact the efficacy of immunotherapies, such as PD-1/PD-L1 inhibitors, in lung cancer treatment.^22–24^

m^6^A regulators and modified mRNAs can serve as potential biomarkers for lung cancer diagnosis, prognosis, and treatment response. For instance, high levels of METTL3 expression have been associated with poor prognosis in lung cancer patients.^16,25,26^

The m^6^A | lung cancer concept pair co-occurrence exhibits the highest average fold increase in publications (nearly 4.5-fold) of all therapeutic target and cancer type concept pairs (Figure 2), which spotlights it as a truly emerging topic in immuno-oncology. Of note, the pair shows a marked increase in publications after 2019, growing by 8-fold over a two-year period (Figure 3).

#### m6A | Liver Cancer and m6A | Hepatocellular Carcinoma (HCC)

The m^6^A | liver cancer and m^6^A | HCC concept pairs are other combinations exhibiting high average co-occurrence growth rate (Figure 2), thus drawing attention as emerging topics in immuno-oncology.

m^6^A methylation is also critical in the context of liver cancer, particularly HCC, the most common type of liver cancer, influencing tumor biology and the immune response.^27–33^

Key m^6^A regulators, including methyltransferases (writers such as METTL3 and METTL14), demethylases (erasers such as FTO and ALKBH5), and m^6^A-binding proteins (readers such as YTHDF1–3), play critical roles in HCC cell proliferation and survival.^10,34,35^ For instance, METTL3-mediated m^6^A modification has been shown to enhance the stability of mRNAs that promote HCC progression.^10,36^

m^6^A modifications can regulate the epithelial-mesenchymal transition (EMT) process, which is essential for cancer metastasis. Changes in m^6^A levels can influence the expression of EMT-related genes, thereby affecting the invasive and metastatic potential of HCC cells.^37,38^

m^6^A modifications play a role in maintaining the stemness^39^ of cancer stem cells, which are implicated in HCC initiation and recurrence. For example, METTL3 can enhance the expression of stemness-related genes, contributing to the self-renewal capacity of cancer stem cells.^40,41^

m^6^A modifications can influence the immune microenvironment of HCC by regulating the expression of immune-related genes and cytokines. This can impact the response to immunotherapies, such as checkpoint inhibitors, in HCC treatment.^22,42,43^

While the m^6^A | liver cancer concept pair exhibits a lower average growth rate (around 3-fold) as compared to the m^6^A | lung cancer concept pair, the growth in publications shows a decidedly upward trend, increasing by 11-fold over 2020–2022 (Figure S2).

#### TAMs | Ovarian Cancer

Tumor-associated macrophages (TAMs) are critical components of the ovarian cancer microenvironment, promoting tumor growth, immune evasion, and metastasis.^44,45^ Targeting TAMs presents a promising strategy in immuno-oncology to improve the efficacy of existing treatments and develop new therapeutic approaches.^46,47^ Ongoing research and clinical trials are actively trying to further clarify the roles of TAMs and optimize strategies for modulating their function in ovarian cancer.^48,49^

Ovarian cancer cells secrete chemokines (e.g., CCL2, CCL5) and cytokines (e.g., CSF-1, IL-6) that recruit and polarize macrophages to the tumor site. Furthermore, they play a role in tumor progression: (i) TAMs inhibit T cell function and promote regulatory T (T_reg_) cells, facilitating immune evasion; (ii) TAMs secrete pro-angiogenic factors such as vascular endothelial growth factor (VEGF), promoting blood vessel formation; (iii) TAMs facilitate metastasis by degrading the extracellular matrix and promoting cancer cell invasion.^50–55^

The TAMs | ovarian cancer co-occurrence has been observed in a fair number of papers with an average fold increase in publications being between 3.5- to 4-fold for the period 2020–2022 (Figure 2) to qualify it as an emerging topic. Similar to other identified concept pairs, the TAMs | ovarian cancer pair shows a 7-fold increase in publications from 2021 to 2022 (Figure 3).

#### CD8 | Colon Cancer

CD8+ T cells play a crucial role in the immune response against colon cancer, particularly in the context of immuno-oncology. These cells are pivotal for antitumor immunity, and their presence and activity within the tumor microenvironment (TME) are associated with better clinical outcomes. CD8+ T cells can directly kill cancer cells through the release of perforin and granzymes, which induce apoptosis in target cells.^56,57^ They produce cytokines such as IFN-γ and TNF-α, which have antitumor effects and can enhance the function of other immune cells.^58–61^

The presence of CD8+ tumor-infiltrating lymphocytes (TILs) within the tumor is often correlated with improved prognosis and survival in colon cancer patients. CD8+ T cells are essential for the immune system’s ability to recognize and eliminate emerging cancer cells, preventing tumor development and progression.^62–65^

Thus, CD8+ T cells are central to the immune response against colon cancer, and their activity is a key determinant of patient outcomes. Immuno-oncology strategies, including ICIs, adoptive cell transfer, cancer vaccines, and oncolytic viruses, leverage the power of CD8+ T cells to combat colon cancer. Ongoing research aims to enhance the effectiveness of these therapies by improving T cell infiltration, overcoming immune evasion mechanisms, and personalizing treatment approaches based on individual tumor profiles.^66–68^

With an average fold increase of nearly 3.5-fold for the period 2020–2022, the CD8 | colon cancer concept pair is among the fastest growing ones (Figure 2).

#### PD-1 | Colorectal Cancer (CRC)

Programmed cell death protein 1 (PD-1) and its ligand PD-L1 play crucial roles in immune evasion by cancer cells, including in colorectal cancer (CRC). In immuno-oncology, targeting the PD-1/PD-L1 pathway has emerged as a promising therapeutic strategy for various cancers.^69–74^

PD-1 is an inhibitory receptor expressed on the surface of T cells, B cells, and natural killer (NK) cells. When PD-1 binds to its ligands, PD-L1 or PD-L2, it transmits an inhibitory signal that reduces T cell activity, leading to decreased proliferation, cytokine production, and cytotoxic activity.^75,76^

PD-L1 is often upregulated on the surface of cancer cells and TAMs within the TME. This upregulation helps tumors evade immune surveillance by inhibiting the activity of PD-1-expressing T cells.^77–80^

The PD-1/PD-L1 pathway plays a pivotal role in immune evasion by CRC, particularly in microsatellite instability-high (MSI-H)/mismatch repair-deficient (dMMR) tumors. US FDA-approved PD-1 inhibitors such as pembrolizumab and nivolumab have shown significant efficacy in this subset of patients, leading to improved clinical outcomes. Combination therapies and emerging approaches are being actively investigated to extend the benefits of PD-1 blockade to a broader range of CRC patients, including those with microsatellite stable (MSS) tumors. Understanding and targeting the underlying mechanisms of resistance and optimizing therapeutic strategies will be crucial for advancing the treatment of colorectal cancer in the field of immuno-oncology.^73,74,81–84^

The PD-1 | colorectal cancer concept pair appears to be on the higher end of the spectrum, with an average fold increase of >3-fold (Figure 2).

#### PD-1 | Liver Cancer

Liver cancer, particularly HCC, has been a significant focus in the field of immuno-oncology due to the unique liver immune environment and the high prevalence of chronic liver diseases that predispose to cancer development. Liver immune tolerance is primarily regulated by various mechanisms, including the PD-1/PD-L1 pathway, which helps prevent autoimmunity but can also facilitate immune evasion by liver tumors.^85–88^

HCC cells and the surrounding stromal cells can express high levels of PD-L1. When PD-L1 binds to PD-1 on T cells, it inhibits T cell activation and proliferation, reduces cytokine production, and promotes T cell exhaustion. The liver’s microenvironment is immunosuppressive, which helps in maintaining tolerance to various antigens. TAMs and other immune cells in the liver can express PD-L1, contributing further to the immunosuppressive milieu.^89–93^

Drugs that block the PD-1/PD-L1 pathway can restore T cell activity and enhance the immune system's ability to target and kill cancer cells.^79,94^

The PD-1 | liver cancer concept pair is an example of co-occurring concepts that increased on average at a modest rate (2-fold) over 2020–2022 while having a relatively high number of publications overall (Figure 2), indicating that it is perhaps more well-established than the rest.

#### PD-L1 | Gastric Cancer

Gastric cancer is one of the leading causes of cancer-related deaths worldwide.^95,96^ The PD-1/PD-L1 pathway has emerged as a significant target in the treatment of gastric cancer due to its role in immune evasion by tumors. Gastric cancer cells can express PD-L1, which binds to PD-1 receptors on T cells, leading to suppression of T cell activity. This interaction inhibits the immune response, allowing cancer cells to grow and proliferate unchecked. The TME in gastric cancer is often immunosuppressive, with various cells (e.g., TAMs, myeloid-derived suppressor cells) expressing PD-L1 and contributing to immune evasion.^97–100^

Besides being a therapeutic target, a relationship has been reported between PD-L1 expression and the clinical features, molecular markers, and molecular subtypes of gastric cancer, which highlights the possible prognostic role of PD-L1, especially soluble PD-L1 and exosomal PD-L1 in patients with gastric cancer.^101–103^

#### HER2 | Nonsmall Cell Lung Cancer (NSCLC)

Human epidermal growth factor receptor 2 (HER2) is a member of the ErbB family of receptor tyrosine kinases, which is involved in the regulation of cell growth and differentiation. HER2 overexpression and amplification are well-known in breast cancer, but they also play a significant role in other cancers, including nonsmall cell lung cancer (NSCLC). HER2 alterations in NSCLC can include overexpression, gene amplification, and mutations. These alterations are less common in NSCLC compared to breast cancer, but they represent a distinct subset of lung cancer patients with specific therapeutic implications. HER2 overexpression and amplification lead to increased signaling through the HER2 pathway, promoting cell proliferation and survival. HER2 mutations are often found in the kinase domain; these mutations can activate HER2 signaling independently of ligand binding, driving oncogenesis.^104–108^

The heterogeneity of HER2 alterations is a challenge—the diversity of HER2 alterations (overexpression, amplification, mutations) in NSCLC requires tailored therapeutic approaches.^109,110^

The role of ICIs in HER2-positive NSCLC is an area of active investigation. Combining ICIs with targeted therapies against HER2 is being explored to enhance antitumor efficacy through combined mechanisms of action.^111–113^

While targeted therapy is available for some cancers driven by HER2, people living with HER2-mutated NSCLC have high unmet needs. In 2022, the US FDA approved the anti-HER antibody-drug conjugate (ADC) trastuzumab deruxtecan (T-DXd, Enhertu) for the treatment of advanced NSCLC driven by mutant HER2.^114^ Trastuzumab deruxtecan was previously approved for the treatment of HER2-positive breast cancer, including in patients with low levels of the HER2 protein.^115^ Recently, a highly selective and potent small molecule inhibitor of HER2, zongertinib, a HER2 tyrosine kinase inhibitor, has been reported as a potential treatment for patients with NSCLC with a specific mutation in the HER2 gene.^116^ These recent approvals correlate well with the increase in publications over 2020–2022 (Figure 2 and Figure S3) and indicate this concept pair might be one to look out for in the future.

#### HER2 | Gastric Cancer

HER2 is a protein that plays a critical role in the regulation of cell growth and differentiation and overexpression or amplification of HER2 is found in a subset of gastric cancers, having significant implications for treatment and prognosis.^117,118^

HER2 is a receptor tyrosine kinase that, when overexpressed or amplified, promotes tumor cell proliferation, survival, and metastasis through activation of downstream signaling pathways like PI3K/AKT and MAPK.^119,120^

HER2-positive gastric cancer can be targeted with therapies that specifically inhibit HER2, thus blocking the growth-promoting signals.^121,122^ Approximately 15–20% of gastric cancers exhibit HER2 overexpression or amplification, making it a significant subset of the disease. HER2-positive gastric cancer is often associated with more aggressive disease and poorer prognosis, although HER2-targeted therapies can improve outcomes.^119,123^

Trastuzumab (Herceptin), a monoclonal antibody, is the first HER2-targeted therapy approved for HER2-positive advanced gastric cancer, typically used in combination with chemotherapy. Trastuzumab binds to the HER2 receptor, preventing activation of downstream signaling pathways and promoting antibody-dependent cellular cytotoxicity (ADCC).^124^

Ado-trastuzumab emtansine (T-DM1) is an ADC combining the monoclonal antibody trastuzumab with a cytotoxic agent (emtansine), enabling delivery of emtansine directly to HER2-positive cancer cells, leading to cell death. It is being investigated in clinical trials for HER2-positive gastric cancer.^125,126^

Pertuzumab (Perjeta) is another monoclonal antibody that targets a different epitope on the HER2 receptor. It is used in combination with trastuzumab and chemotherapy in clinical trials. Pertuzumab inhibits dimerization of HER2 with other HER receptors, enhancing the blockade of HER2 signaling.^127,128^

Lapatinib (Tykerb) is a small-molecule tyrosine kinase inhibitor that targets both HER2 and EGFR. It is being evaluated for use in HER2-positive gastric cancer. Lapatinib inhibits the kinase activity of HER2, preventing activation of downstream signaling pathways.^129,130^

Combining HER2-targeted therapies with ICIs is an area of active research. ICIs such as pembrolizumab (Keytruda) and nivolumab (Opdivo) are being evaluated in combination with trastuzumab and chemotherapy. HER2-targeted therapies can enhance antigen presentation and promote immune cell infiltration into tumors, potentially improving the efficacy of ICIs.^131–133^

#### CD19 | B-Cell Lymphoma

CD19 is a protein expressed on the surface of B cells, including malignant B cells in various types of B-cell lymphomas.^134,135^ It plays a crucial role in B cell development, activation, and differentiation. CD19 has become a significant target in immuno-oncology, particularly for therapies designed to treat B-cell lymphomas. CD19 is uniformly expressed across all stages of B-cell development, making it an ideal target for therapies aimed at eliminating B-cell malignancies. CD19 is involved in the B-cell receptor signaling pathway, which is essential for B-cell activation and proliferation. Targeting CD19 disrupts this pathway, leading to the death of malignant B cells.^136–138^

CD19 is expressed in the vast majority of B-cell lymphomas, including diffuse large B-cell lymphoma (DLBCL), follicular lymphoma, mantle cell lymphoma, and chronic lymphocytic leukemia (CLL). CD19 has become the primary target for chimeric antigen receptor T-cell (CAR-T) therapies, which have shown remarkable efficacy in treating relapsed or refractory B-cell lymphomas.^139,140^

##### CAR-T Cell Therapy

- Tisagenlecleucel (Kymriah) has been approved for the treatment of relapsed or refractory DLBCL and pediatric/young adult acute lymphoblastic leukemia (ALL). T cells are engineered to express a CAR that specifically targets CD19, leading to the destruction of CD19-positive B cells.^141,142^

- Axicabtagene ciloleucel (Yescarta) is approved for the treatment of relapsed or refractory large B-cell lymphoma, including DLBCL, primary mediastinal large B-cell lymphoma, and transformed follicular lymphoma. Similar to Kymriah, Yescarta involves engineering T cells to target CD19, enhancing the immune system’s ability to eliminate cancerous B cells.^143,144^

- Lisocabtagene maraleucel (Breyanzi) is approved for the treatment of relapsed or refractory large B-cell lymphoma. It is another CAR-T therapy targeting CD19, designed to provide a robust and durable response in B-cell lymphoma patients.^145,146^

##### Monoclonal Antibodies and Antibody–Drug Conjugates

- Blinatumomab (Blincyto) is a bispecific T-cell engager (BiTE) that targets CD19 and CD3, approved for the treatment of B-cell precursor ALL. Blinatumomab binds to CD19 on B cells and CD3 on T cells, facilitating the immune-mediated destruction of B cells.^147,148^

- Loncastuximab tesirine (Zynlonta) is an ADC targeting CD19, approved for relapsed or refractory large B-cell lymphoma. The monoclonal antibody component targets CD19, delivering a cytotoxic payload directly to the malignant B cells.^149–151^

In summary, the efficacy of immunotherapies depends on the interplay between specific molecular targets and the TME of particular cancer types. Table 1 summarizes representative co-occurring therapeutic targets and specific cancer types along with their clinical prospects.

**Table 1:** Summary of Representative Therapeutic Targets and Specific Cancer Types along with Their Associated Therapies and Clinical Prospects

**therapeutic target**	**cancer type**	**associated therapy**	**clinical prospects**
m6A modification	lung cancer	METTL3/FTO inhibitors	enhances anti-PD-1 response by modulating PD-L1 translation; clinical trials ongoing
m6A modification	liver cancer (HCC)	YTHDF2/ALKBH5 inhibitors	improves NK cell function; combinations with ICIs in development
TAMs (CSF1R+)	ovarian cancer	CSF1R inhibitors (pexidartinib) + anti-PD-1	reverses immunosuppression; phase II trials show improved T-cell infiltration
CD8+ T cells	colon cancer	neoantigen vaccines/OX40 agonists	high CD8+ infiltration correlates with survival; vaccines boost response
PD-1/PD-L1	colorectal cancer (MSI-H)	pembrolizumab/nivolumab	MSI-H tumors show 40–50% response; MSS tumors require combinations (e.g., MEK inhibitors)
HER2	NSCLC (HER2+)	trastuzumab deruxtecan (T-DXd)	objective response rate (ORR) ∼55% in HER2-mutated NSCLC; combinations with ICIs under investigation

### In-Sentence Co-Occurrences: Biomarkers and Types of Cancer

Biomarkers play a critical role in immuno-oncology, aiding in the identification, characterization, and treatment of various cancers.^152–155^ In recent immuno-oncology publications, the co-occurrence of biomarkers with key cancer concepts highlights their importance in understanding TME, predicting treatment responses, and developing targeted therapies. The identification of biomarkers and their co-occurrence with cancer concepts enables personalized medicine approaches, tailoring treatments based on individual patient profiles and tumor characteristics. Biomarker-driven clinical trials focus on enrolling patients based on specific biomarker profiles, improving the likelihood of treatment success, and accelerating the development of new therapies. The co-occurrence of biomarkers and cancer concepts in immuno-oncology publications underscores the complexity and interdependence of immune responses and cancer biology. Advanced analytical techniques are essential for uncovering these relationships, ultimately leading to more effective and personalized cancer immunotherapies.

As in the previous section, highlighted below in greater detail are a few representative concept pairs with high growth (TMB | colon cancer, TMB | CRC, TMB | head neck squamous cell carcinoma (HNSCC), HER2 | gastric cancer, nectin-4 | bladder cancer, and GATA3 | bladder cancer) and concept pairs with moderate growth with relatively higher number of publications (TMB | NSCLC, CD19 | B-cell lymphoma and PD-L1 | liver cancer) (Figure 4).

**Figure 4:**
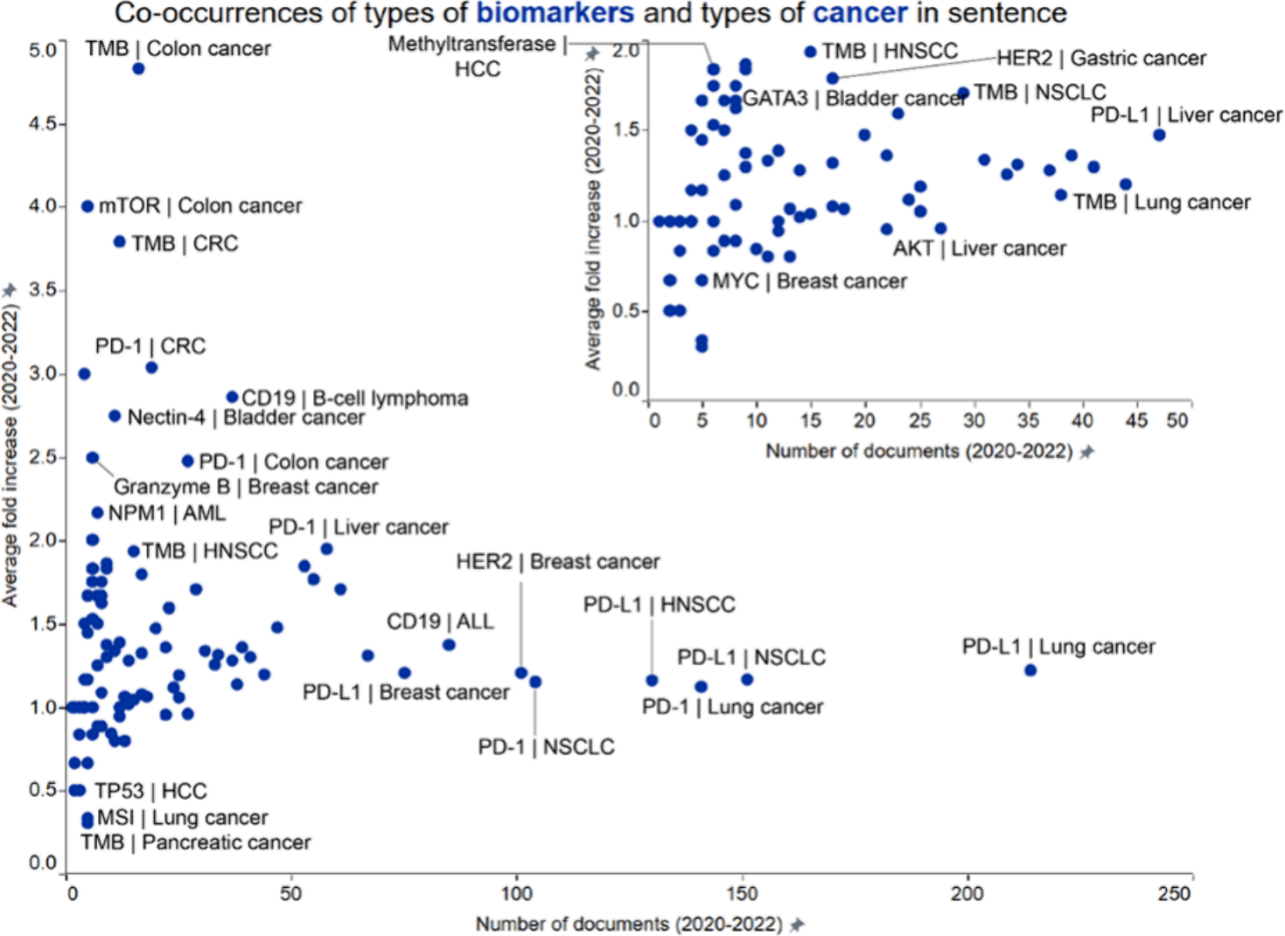
Co-occurrence analysis of emerging concepts in immuno-oncology focused on biomarkers and cancer types. Concepts pairs co-occurred within a sentence in the title or abstract of publications. Labels outline certain noteworthy concept pairs exhibiting either a high number of documents (right part of the graph) or a high growth rate (upper left). The inset is a graph focused on data clustered in the lower left corner of the bigger graph.

#### TMB | Colon Cancer; TMB | CRC

Tumor mutational burden (TMB) is a biomarker that quantifies the number of mutations within a tumor genome. It is gaining importance in immuno-oncology as a predictor of response to ICIs. High TMB is associated with a higher likelihood of producing neoantigens, which can make tumors more recognizable and targetable by the immune system.^156–158^

High TMB leads to the production of neoantigens, which are novel protein sequences arising from tumor-specific mutations. These neoantigens can be recognized as foreign by the immune system, prompting an immune response.^159,160^

ICIs, such as PD-1/PD-L1 inhibitors, work by unleashing the immune system to attack cancer cells. High TMB can enhance the effectiveness of ICIs by increasing the number of targets (neoantigens) available for immune recognition.^161,162^

A subset of CRCs exhibits high microsatellite instability, which often correlates with high TMB. High microsatellite instability CRCs are more responsive to ICIs. Colorectal cancers with mismatch repair deficiency (dMMR), leading to high microsatellite instability (MSI-H), tend to have a high TMB. These tumors are more likely to respond to ICIs such as pembrolizumab and nivolumab.^163–165^

TMB is an important biomarker in the immuno-oncology landscape for colorectal cancer. It helps identify patients who are more likely to respond to ICIs, particularly in the context of high microsatellite instability or mismatch repair deficiency tumors. Ongoing research is focused on refining the use of TMB, exploring its potential in combination therapies, and integrating it with other biomarkers to enhance the efficacy of immunotherapy in colorectal cancer.^156,158,166–168^

#### TMB | Head and Neck Squamous Cell Carcinoma (HNSCC)

TMB serves as a predictive biomarker for the response to ICIs in head and neck squamous cell carcinoma (HNSCC). Higher TMB in HNSCC is associated with better responses to therapies like pembrolizumab (Keytruda) and nivolumab (Opdivo),^169^ as well as durvalumab and/or tremelimumab.^170–172^

Pembrolizumab is approved for the treatment of recurrent or metastatic HNSCC with PD-L1 expression (CPS ≥ 1). Research indicates that high TMB may also be predictive of response to ICIs in these settings.^173^

#### TMB | NSCLC

High TMB has been shown to predict better responses to ICIs in NSCLC.^174–177^

The US FDA has approved pembrolizumab (Keytruda) for use in patients with high TMB (≥10 mutations per megabase) in various cancers, including NSCLC, regardless of PD-L1 expression. Pembrolizumab has been approved for the treatment of NSCLC with high TMB, particularly in patients who have progressed in other therapies. It has shown efficacy in this patient population due to its ability to enhance the immune response against tumors with high TMB.^171,178–180^ Nivolumab (Opdivo) and ipilimumab (Yervoy): This combination has been studied in patients with high TMB, showing improved outcomes in first-line treatment for NSCLC. The combination leverages the benefits of PD-1 and CTLA-4 inhibition to boost antitumor immunity.^181–183^

Overall, the concept pairs consisting of TMB with colon cancer, HNSCC and NSCLC have fewer publications associated with them (<30) but exhibit a high average fold increase, indicating growing interest (Figure 3 and Figure S1).

#### Nectin-4 | Bladder Cancer

Nectin-4 is a cell adhesion molecule and part of the Nectin family that plays a role in the formation of cell–cell junctions. It is overexpressed in several cancers, including bladder cancer, and has emerged as a significant target in immuno-oncology. Nectin-4 is highly expressed in urothelial carcinoma, which is the most common type of bladder cancer. Its overexpression is associated with tumor progression and a poor prognosis. Nectin-4 contributes to tumor cell proliferation, survival, and metastasis by mediating cell–cell adhesion and interacting with other signaling pathways.^184–187^

High levels of nectin-4 expression can be used as a diagnostic marker for bladder cancer, helping to identify patients who may benefit from targeted therapies. Overexpression of nectin-4 is correlated with more aggressive disease and poorer outcomes, making it a potential prognostic marker.^185,188^

Enfortumab vedotin (Padcev) is an ADC targeting nectin-4, approved for the treatment of locally advanced or metastatic urothelial carcinoma. Enfortumab vedotin consists of a monoclonal antibody specific for nectin-4 linked to a cytotoxic agent (monomethyl auristatin E, MMAE).^189–191^

The nectin-4 | bladder cancer concept pair shows an upward trend with a 6-fold increase in publications between 2020 and 2022 (Figure S1, Supporting Information).

#### PD-1 | Colon Cancer

As previously discussed, PD-1 is an immune checkpoint receptor that is expressed on the surface of T cells. The interaction between PD-1 and its ligands (PD-L1 and PD-L2) inhibits T cell activity, allowing cancer cells to evade immune detection.^192–195^

Colon cancer cells can express PD-L1, which binds to PD-1 on T cells, leading to the inhibition of T cell activity and allowing tumor cells to escape immune surveillance. ICIs targeting PD-1 or PD-L1 block this interaction, reactivating T cells and enhancing their ability to attack cancer cells.^74,87,94^

While expression levels of PD-L1 alone may not be sufficient as predictive biomarkers, there are nonetheless reports studying levels of PD-L1 expression in the context of colon and other cancer types.^196–198^ Instances of these publications appear to be on the rise over the past few years (Figure S1 and Supporting Information).

Besides as biomarkers, therapies involving PD-1/PD-L1 have been approved for colon cancer:

Pembrolizumab (Keytruda) is approved for the treatment of MSI-H/dMMR colorectal cancer (CRC) that is unresectable or metastatic. Pembrolizumab is a monoclonal antibody that binds to PD-1, preventing its interaction with PD-L1 and PD-L2, thus reactivating T cells to attack tumor cells.^199^

Nivolumab (Opdivo) is approved for the treatment of MSI-H/dMMR metastatic CRC. Nivolumab functions similarly to pembrolizumab, blocking the PD-1/PD-L1 interaction and enhancing the immune response against cancer cells.^200–202^

Nivolumab + ipilimumab (Yervoy) combination therapy is approved for MSI-H/dMMR metastatic CRC. Ipilimumab targets CTLA-4, another checkpoint inhibitor, and when combined with nivolumab, provides a synergistic effect that enhances T cell activation and antitumor activity.^201,203^

PD-1 and its ligands play critical roles in the immune evasion mechanisms of colon cancer. ICIs targeting the PD-1/PD-L1 pathway have shown significant efficacy in treating MSI-H/dMMR colorectal cancer. Ongoing research aims to optimize these therapies, overcome resistance, and expand their use to benefit a broader patient population. The integration of PD-1 inhibitors with other treatment modalities holds great promise for the future of colon cancer immunotherapy.^74,204,205^

#### PD-L1 | Liver Cancer

In liver cancer, particularly HCC, PD-1/PD-L1 inhibitors have shown promise in enhancing antitumor immune responses. Liver cancer cells can express PD-L1, which binds to PD-1 on T cells, leading to the inhibition of T cell activation and allowing tumor cells to escape immune detection and destruction. Blocking the interaction between PD-1 and PD-L1 with ICIs reactivates T cells, enabling them to recognize and kill cancer cells.^206–208^

The unique immune environment of the liver, rich in immunosuppressive cells, creates a challenge for effective immune responses. PD-1/PD-L1 inhibitors help to overcome this immunosuppressive microenvironment. High PD-L1 expression in liver cancer is associated with poor prognosis, making it a potential prognostic marker.^209,210^ The PD-L1 | liver cancer biomarker-cancer type concept pair appears to be growing at a moderate pace (the average fold increase being 2-fold) with an upward trend (Figure 3 and Figure S1).

Approved therapies targeting PD-1/PD-L1 and approved for treatment of HCC include the following:

Nivolumab (Opdivo) is an anti-PD-1 antibody approved for the treatment of HCC in patients who have been previously treated with sorafenib. Nivolumab blocks the interaction between PD-1 and its ligands, reactivating T cells to target and destroy cancer cells.^211,212^

Pembrolizumab (Keytruda) is another anti-PD-1 antibody approved for HCC patients who have been treated with sorafenib. Pembrolizumab functions similarly to nivolumab, enhancing the immune system’s ability to fight cancer by blocking the PD-1/PD-L1 interaction.^213,214^

Combination Therapies

- Atezolizumab (Tecentriq) and Bevacizumab (Avastin): This combination has been approved for the first-line treatment of patients with unresectable or metastatic HCC. Atezolizumab is an anti-PD-L1 antibody that blocks PD-L1 from binding to PD-1, while bevacizumab is an anti-VEGF antibody that inhibits angiogenesis. The combination works synergistically to enhance immune responses and inhibit tumor blood supply.^215–217^

PD-1 and its ligands play a significant role in immune evasion mechanisms of liver cancer. ICIs targeting the PD-1/PD-L1 pathway have shown promising results in treating advanced HCC, particularly in patients who have failed prior therapies. Ongoing research aims to optimize these therapies, manage associated toxicities, overcome resistance mechanisms, and expand their use to benefit a broader patient population. The integration of PD-1 inhibitors with other treatment modalities holds great promise for the future of liver cancer immunotherapy.^87,218,219^

#### GATA3 | Bladder Cancer

GATA binding protein 3 (GATA3) is a transcription factor that plays a crucial role in the development and differentiation of various tissues, including the urothelium. In the context of bladder cancer, GATA3 has emerged as a significant biomarker with implications for diagnosis, prognosis, and potential therapeutic targeting. GATA3 regulates the expression of genes involved in cell differentiation and proliferation. Its expression is crucial for maintaining the normal function of the urothelium. Depending on the context, GATA3 can act as either a tumor suppressor or an oncogene. In bladder cancer, its role is more complex and is associated with specific molecular subtypes.^220,221^

GATA3 is highly expressed in urothelial carcinoma, making it a useful diagnostic marker for bladder cancer.^222,223^ It is often used in immunohistochemistry (IHC) to confirm the diagnosis of urothelial carcinoma. The expression level of GATA3 can also provide prognostic information. Higher levels of GATA3 are often associated with better differentiation and a less aggressive phenotype of bladder cancer. GATA3 expression is a characteristic of the luminal subtype of bladder cancer, which generally has a better prognosis and distinct therapeutic responses compared to other subtypes.^221,224^

GATA3 is widely used in IHC panels to diagnose urothelial carcinoma. It helps distinguish bladder cancer from other malignancies, particularly in metastatic settings where the primary origin of the tumor might be unclear.^222,225^

Ongoing research aims to further elucidate the role of GATA3 in bladder cancer biology and to explore its potential as a target for therapeutic intervention. Integrating GATA3 with other biomarkers and clinical parameters will enhance its utility in the management of bladder cancer.^226,227^

#### MSI | Gastric Cancer

Microsatellite instability (MSI) is a condition of genetic hypermutability that results from impaired DNA mismatch repair (MMR).^228,229^ MSI is characterized by alterations in the length of microsatellites, which are short, repetitive sequences of DNA. MSI is a significant biomarker in various cancers, including gastric cancer, and has implications for diagnosis, prognosis, and therapeutic strategies, particularly in immuno-oncology. MSI occurs due to defects in the DNA MMR system, leading to the accumulation of mutations throughout the genome. Key MMR genes include MLH1, MSH2, MSH6, and PMS2. The accumulation of mutations results in a high mutation burden, which can produce neoantigens that are recognized by the immune system, making MSI-high (MSI-H) tumors potentially more immunogenic.^230–232^

MSI-H is found in approximately 10–20% of gastric cancer cases.^233^ It is more commonly observed in early-stage tumors and specific histological subtypes. MSI-H status in gastric cancer is generally associated with a better prognosis compared to microsatellite stable (MSS) tumors. MSI-H gastric cancers are more likely to respond to ICIs due to their high mutation burden and resultant neoantigen load.^234,235^

Diagnostic Use: MSI status can be determined using polymerase chain reaction (PCR) to analyze microsatellite markers or immunohistochemistry (IHC) to assess the expression of MMR proteins. Next-generation sequencing (NGS) is also increasingly used for MSI detection.^236–238^

##### Approved Therapies

- Pembrolizumab (Keytruda) is approved for the treatment of MSI-H or MMR-deficient solid tumors, including gastric cancer, that are unresectable or metastatic and have progressed following prior treatment. Pembrolizumab is an anti-PD-1 antibody that blocks the interaction between PD-1 on T cells and its ligands (PD-L1/PD-L2) on tumor cells, reactivating T cells to attack the tumor.^239^
- Trials are evaluating the combination of ICIs with chemotherapy, targeted therapies, and other immunotherapies to enhance antitumor responses in MSI-H gastric cancer.^240,241^

- Research is focused on identifying additional biomarkers that can predict response to immunotherapy in gastric cancer, as well as understanding the mechanisms underlying resistance to ICIs.^242,243^

In summary, the success of immunotherapy depends on identifying predictive biomarkers that stratify patients’ likelihood to respond. Biomarker-cancer co-occurrence patterns reveal critical insights into tumor-immune interactions and therapeutic vulnerabilities. The co-occurrence of biomarkers and types of cancer highlights the importance of cancer-type-specific immunotherapy targeting. Future success depends on precision biomarker development, rational combinations, and mechanistic insights into target-cancer interactions. Table 2 summarizes certain representative co-occurring biomarker/cancer type combinations with their clinical prospects.

**Table 2:** Summary of Representative Biomarker/Cancer Type Combinations along with Their Clinical Significances

**biomarker**	**cancer type**	**clinical significance**
TMB	colon cancer (MSI-H)	high TMB predicts response to immune checkpoint inhibitors (ICIs)
TMB	colorectal cancer (MSS)	low TMB correlates with poor ICI response; combinations (MEK inhibitors) under investigation
TMB	head and neck squamous cell carcinoma (HNSCC)	HPV+ tumors have lower TMB but better ICI response due to immune-rich TME
HER2	gastric cancer	HER2+ gastric cancer benefits from trastuzumab + chemo; ADCs (T-DXd) show promise
nectin-4	bladder cancer (urothelial carcinoma)	highly expressed in advanced disease; target for enfortumab vedotin (ADC)
GATA3	bladder cancer	luminal subtype marker; may predict response to FGFR inhibitors (erdafitinib)
TMB	nonsmall cell lung cancer (NSCLC)	high TMB (≥10 mut/Mb) linked to improved progression-free survival (PFS) with nivolumab + ipilimumab
CD19	B-cell lymphoma (DLBCL, ALL)	target for CAR-T (tisagenlecleucel, axicabtagene ciloleucel) and bispecific antibodies
PD-L1	liver cancer (HCC)	heterogeneous expression; atezolizumab + bevacizumab benefits PD-L1+ patients

### In-Sentence Co-Occurrences: Types of Therapy and Types of Cancer

In immuno-oncology, the co-occurrence of therapy concepts and cancer types provides valuable insights into the interconnectedness of various therapeutic strategies and their applications across different malignancies. Analyzing these co-occurrences helps identify effective therapeutic strategies, research trends, and potential areas for future investigation. This understanding is crucial for developing more effective and personalized cancer immunotherapies, ultimately improving patient outcomes. Shown in Figure 5 are results from our co-occurring analysis focused on therapy and cancer types, with yearly trends of a few chosen pairs highlighted in Figure S2 (Supporting Information). All the pairs highlighted in Figure S2 show an upward trend, but none as distinctly as CAR-T | multiple myeloma (MM), showing an ∼7-fold increase in publications in a 2-year period starting 2019.

**Figure 5:**
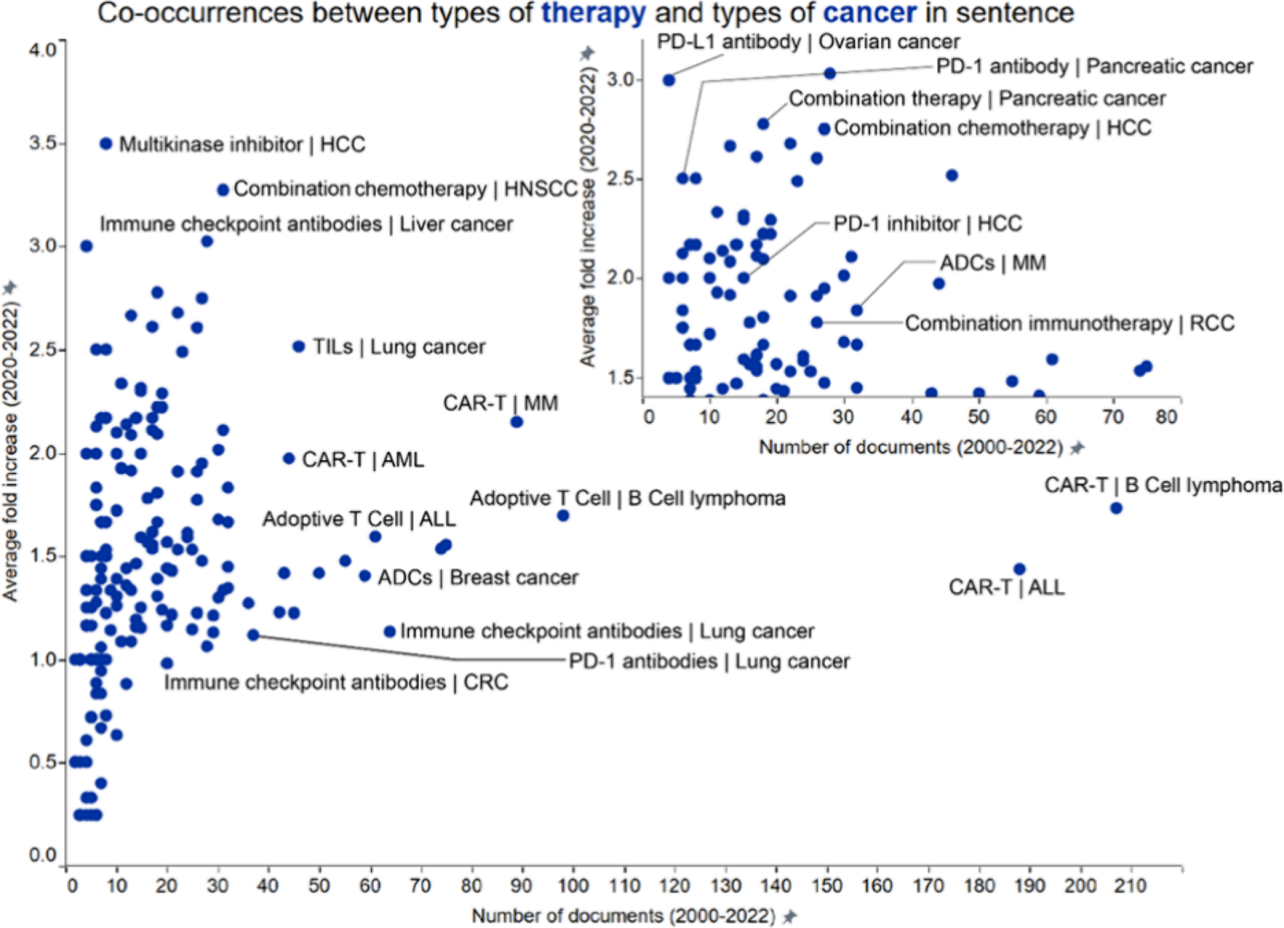
Co-occurrence analysis of emerging concepts in immuno-oncology focused on types of therapy and cancer types. Concepts pairs co-occurred within a sentence in the title or abstract of publications. Labels outline certain noteworthy combinations exhibiting either high number of documents (right part of the graph) or high growth rate (upper left). Shown inset is a graph focused on data clustered in the lower left corner of the bigger graph.

#### Immune Checkpoint Antibodies | Liver Cancer

Traditionally, liver cancer has been challenging to treat with limited options for advanced stages. The introduction of ICIs has provided a new hope for these patients. In liver cancer, ICIs work by blocking the interaction between PD-1 on T cells and PD-L1 on tumor cells, effectively removing the ″brake″ on the immune system and allowing T cells to attack and destroy cancer cells.^244–246^

The most common ICIs target PD-1, PD-L1 (Programmed Death-Ligand 1), and CTLA-4. PD-1 inhibitors include nivolumab and pembrolizumab; PD-L1 inhibitors: atezolizumab and durvalumab; CTLA-4 inhibitors: ipilimumab. These therapies have revolutionized the treatment of various cancers, including melanoma, lung cancer, and more recently, liver cancer. Nivolumab and pembrolizumab have shown promise in treating advanced HCC, especially in patients who have progressed or are intolerant of sorafenib (a standard therapy for HCC). The combination of a PD-L1 inhibitor, atezolizumab, and a VEGF inhibitor, bevacizumab, has demonstrated significant improvement in survival rates compared to sorafenib and has become a new standard of care for unresectable HCC.^94,205,247,248^

#### TILs | Lung Cancer

Tumor-infiltrating lymphocytes (TILs) are a subset of immune cells that have migrated from the bloodstream into the TME.^249,250^ These cells, primarily T cells, recognize and attack tumor cells, playing a critical role in the body’s natural immune response to cancer. The presence, density, and activity of TILs within tumors have significant prognostic and therapeutic implications in various cancers, including lung cancer.^65,251^

The presence and characteristics of TILs in lung cancer have been found to correlate with patient outcomes—a high density of TILs, particularly CD8+ cytotoxic T cells, is generally associated with a better prognosis in NSCLC. However, TILs are not just passive indicators of the immune response; they can be actively harnessed for cancer treatment, particularly through immunotherapy.^252,253^

The success of ICIs in lung cancer, such as PD-1/PD-L1 inhibitors (e.g., nivolumab, pembrolizumab, and atezolizumab), is partly due to their ability to reinvigorate exhausted TILs, enabling them to mount a more effective antitumor response. The density and activation status of TILs can predict the response to these therapies. Patients with higher levels of TILs, particularly PD-1-expressing T cells, are more likely to respond to ICIs.^94,254^

Although less developed in lung cancer compared to melanoma, adoptive TIL therapy involves extracting TILs from a patient’s tumor, expanding them in the lab, and reinfusing them into the patient. This strategy has shown promise in other cancers and is being explored in lung cancer as well.^255–258^

The analysis of TILs can serve as a biomarker for predicting response to immunotherapy in lung cancer. Patients with ″hot″ tumors, characterized by high TIL infiltration and PD-L1 expression, tend to respond better to ICIs than those with ″cold″ tumors, which lack significant immune cell infiltration.^259,260^

Combining TIL-based approaches with other treatments such as chemotherapy, radiation, or other forms of immunotherapy is an area of active research. These combinations may enhance the infiltration, activation, and persistence of TILs in the TME.^261,262^

Advances in single-cell sequencing, spatial transcriptomics, and other high-resolution techniques provide deeper insights into the composition and function of TILs within lung tumors. These technologies may lead to the development of more personalized immunotherapies based on the specific TIL profile of a patient’s tumor.^263–265^

#### CAR-T | Multiple Myeloma (MM)

Chimeric antigen receptor T-cell (CAR-T) therapy is a form of immunotherapy where patient T cells are genetically engineered to express receptors specific to cancer cells. These engineered T cells are then expanded and reinfused into the patient, where they seek out and destroy cancer cells. CAR-T therapy has shown remarkable success in treating certain blood cancers, particularly B-cell lymphomas and ALL.^140,266–268^

The success of CAR-T therapy depends on identifying suitable target antigens that are abundantly expressed on cancer cells but not on healthy cells. For MM, the most prominent targets are (i) B-cell maturation antigen (BCMA), a protein highly expressed on MM cells, which is critical for their survival. It is the most widely studied and targeted antigen in CAR-T therapy for MM; (ii) while BCMA is the primary target, other antigens such as SLAMF7, CD19, CD138, and GPRC5D are also being explored for CAR-T therapy, particularly in patients who relapse after BCMA-targeted therapy.^269–275^

Approved and investigational CAR-T therapies include the following:

Idecabtagene vicleucel (Abecma) is a BCMA-targeting CAR-T therapy approved in 2021 as the first US FDA-approved CAR-T therapy for MM. It is indicated for patients who have received at least four prior therapies, including an immunomodulatory agent, a proteasome inhibitor, and an anti-CD38 antibody.^276,277^

Ciltacabtagene autoleucel (Carvykti), another BCMA-targeting CAR-T therapy approved in 2022, shows deep and durable responses in heavily pretreated MM patients.^278,279^

Numerous other BCMA-targeting CAR-T products are under investigation, along with CAR-T therapies targeting alternative antigens. These therapies are being evaluated in clinical trials for their efficacy, safety, and ability to provide long-term remission.^273,280^

#### CAR-T | Acute Myeloid Leukemia (AML)

CAR-T cell therapy application in AML has been challenging due to several factors: (i) AML lacks a universally expressed, leukemia-specific antigen; (ii) AML is highly heterogeneous, with different patients exhibiting varied expression of potential target antigens; (iii) the bone marrow microenvironment in AML is often immunosuppressive, which can inhibit the function and persistence of CAR-T cells. AML cells and their microenvironment can produce factors that suppress T cell activity or promote T cell exhaustion.^281,282^

Despite these challenges, several antigens have been investigated as potential CAR-T cell targets in AML:

- CD33 is expressed on the majority of AML cells, making it a promising target. However, CD33 is also found on normal myeloid cells, including progenitor cells, leading to concerns about myeloablation and prolonged cytopenias if CAR-T cells target healthy cells.^283,284^

- CD123 (IL-3 receptor alpha chain) is another common target in AML. It is expressed on AML blasts and leukemic stem cells. However, like CD33, CD123 is also present on normal hematopoietic stem and progenitor cells, posing similar toxicity risks.^285,286^

- FLT3 is a receptor tyrosine kinase often mutated in AML, leading to its overexpression in AML cells. CAR-T cells targeting FLT3 are being explored, but concerns remain regarding undesirable effects on normal hematopoietic cells.^287,288^

- CLL-1 (CLEC12A) is expressed on AML cells and leukemic stem cells, but not on normal hematopoietic stem cells, making it a potentially safer target. However, its expression is not universal across all AML cases.^289,290^

- To address the issue of antigen heterogeneity, dual-targeting CAR-T cells that recognize two different antigens (e.g., CD33 and CD123) are being developed. These approaches aim to reduce the likelihood of AML escape via antigen loss.^291,292^

Combining CAR-T therapy with other treatments like ICIs, hypomethylating agents, or kinase inhibitors is being explored to enhance CAR-T cell efficacy and persistence while reducing toxicities.^293,294^

#### CAR-T | Colon Cancer

CAR-T cell therapy application to solid tumors like colon cancer poses significant challenges: (i) a major hurdle is identifying antigens that are highly expressed on tumor cells but not on normal tissues, to avoid on-target, off-tumor toxicity; (ii) TME in colon cancer is highly immunosuppressive; (iii) colon cancer is characterized by heterogeneity in antigen expression; (iv) colon cancer cells can downregulate antigen presentation and secrete immunosuppressive factors, making it difficult for CAR-T cells to recognize and attack them.^295–297^

Despite these challenges, several antigens and strategies are being investigated for CAR-T cell therapy in colon cancer:

- CEA (carcinoembryonic antigen) is a well-known tumor-associated antigen overexpressed in many colon cancers but also present at lower levels in normal tissues, particularly in the gastrointestinal tract. CAR-T cells targeting CEA have been developed and tested in early-phase clinical trials. However, on-target, off-tumor toxicity, such as gastrointestinal inflammation, remains a significant concern.^298–300^

- HER2 is another potential target for CAR-T therapy in colon cancer, especially in cases where HER2 is overexpressed. HER2-targeted CAR-T cells have been tested, but again, the risk of damaging normal tissues that express HER2 limits their application.^301–303^

- EGFR is overexpressed in some colon cancers, making it a target for CAR-T therapy. EGFR-targeted CAR-T cells have been explored, but their use is complicated by the widespread expression of EGFR in normal epithelial tissues, leading to potential toxicities.^303–305^

- GUCY2C (guanylate cyclase C) is a more specific antigen for gastrointestinal malignancies, including colon cancer, and is not significantly expressed in extraintestinal tissues. CAR-T cells targeting GUCY2C have shown promise in preclinical models, with ongoing research aimed at optimizing their safety and efficacy.^306–308^

- Mesothelin is another antigen that has been explored as a target for CAR-T therapy in various solid tumors, including colon cancer. While it is not as widely expressed in normal tissues, its role in colon cancer remains under investigation.^309–311^

The continued co-occurrence of CAR-T with hematological malignancies such as MM and AML, exhibiting upward trends in terms of the number of publications, is indicative of the persistent pursuit/development and potential of CAR-T for the treatment of liquid tumors. Their moderate average fold increase and relatively high number of publications indicate a high degree of establishment as compared to the emergence of CAR-T in the context of colon cancer, a type of solid tumor, which perhaps represents the increasing efforts to develop CAR-T for solid cancers.

#### PD-1 Antibody | Pancreatic Cancer

PD-1 antibody therapy has had limited success in pancreatic cancer due to the tumor’s unique biology and immunosuppressive microenvironment.^312,313^ However, ongoing research into combination therapies, stromal modulation, and biomarker-driven approaches holds promise for improving outcomes in this difficult-to-treat cancer.^314,315^

Despite these challenges, there has been ongoing research into the use of PD-1 and PD-L1 inhibitors in pancreatic cancer, often in combination with other therapies. PD-1 inhibitors like pembrolizumab (Keytruda) and nivolumab (Opdivo) have been tested as monotherapies in pancreatic cancer with disappointing results. Most clinical trials have reported low response rates and minimal impact on survival when these agents are used alone.^316–318^

There is ongoing research into the use of PD-1 and PD-L1 inhibitors in pancreatic cancer, in combination with other therapies: chemotherapy, radiation therapy, targeted therapies, other immunotherapies, and stromal modulation:

- Pembrolizumab with chemotherapy^319,320^

- Nivolumab with ipilimumab^321–323^

- Targeting the microbiome^324–326^

#### PD-L1 Antibody | Ovarian Cancer

Challenges in PD-L1 antibody therapy for ovarian cancer include: (i) PD-L1 expression in ovarian cancer is variable and often heterogeneous; (ii) the TME in ovarian cancer is typically immunosuppressive, containing regulatory T cells (Tregs), myeloid-derived suppressor cells, and TAMs that inhibit immune responses; (iii) ovarian cancer is highly heterogeneous, with multiple histological subtypes; (iv) ovarian cancer generally has a lower TMB.^327,328^

Several PD-L1 inhibitors have been tested in ovarian cancer: (i) monotherapy with PD-L1 inhibitors: atezolizumab, durvalumab, and avelumab; (ii) combination therapies: chemotherapy, anti-VEGF therapy, poly (ADP-ribose) polymerase (PARP) inhibitors, vaccines, and adoptive cell therapy, and checkpoint blockade combinations.^329–331^

#### ADCs | MM

Several surface antigens are expressed on multiple myeloma cells that can be targeted by ADCs: BCMA (B-cell maturation antigen); CD38; CD138 (syndecan-1); CD56; SLAMF7 (signaling lymphocytic activation molecule family member 7).^332–334^

Key ADCs in development and clinical use include (i) belantamab mafodotin (Blenrep), the first ADC approved for treatment of multiple myeloma; (ii) other ADCs in development include MEDI2228, CC-99712, and STRO-001.^335–337^

In summary, the selective effectiveness of specific immunotherapies in particular malignancies reflects fundamental differences in tumor immunobiology, microenvironment composition, and immune evasion mechanisms. Table 3 summarizes representative combinations of cancer types/therapies with their clinical prospects.

**Table 3:** Summary of Representative Cancer Type/Therapy Combinations along with Their Clinical Prospects

**therapy**	**cancer type**	**clinical prospects**
immune checkpoint antibodies (e.g., atezolizumab + bevacizumab)	liver cancer (HCC)	improved overall survival vs sorafenib; combinations with tyrosine kinase inhibitors (TKIs) under study
tumor-infiltrating lymphocytes (TILs)	lung cancer (NSCLC)	ORR ∼25% in metastatic NSCLC; enhanced with PD-1 blockade
CAR-T (BCMA-targeted)	multiple myeloma	high ORR (73–98%) in refractory MM; dual-targeting CARs in development
CAR-T (CD123/FLT3-targeted)	acute myeloid leukemia (AML)	early promise but limited by antigen heterogeneity/toxicities
CAR-T (CLDN18.2-targeted)	colon cancer	emerging target for gastrointestinal cancers; challenges with TME suppression
PD-1 antibody (e.g., pembrolizumab)	pancreatic cancer	limited efficacy alone (∼5% ORR); combinations with CD40 agonists or CCR2 inhibitors
PD-L1 antibody (e.g., avelumab)	ovarian cancer	modest activity alone; better in combination with PARP inhibitors or CTLA-4
antibody-drug conjugates (ADCs) (e.g., belantamab mafodotin)	multiple myeloma	BCMA-targeted ADCs show ∼31% ORR; next-gen ADCs with STING payloads in development

### In-Sentence Co-Occurrences: Types of Targets and Types of Therapy

The co-occurrence of targets and therapy concepts in immuno-oncology publications reveals important relationships and insights into how different therapeutic strategies are developed and applied. This involves studying specific molecular targets, immune pathways, and types of therapies that are being investigated and used in the treatment of cancer. Results from our co-occurrence analysis focused on therapeutic targets and therapy types are shown in Figure 6, with yearly trends of a few chosen pairs highlighted in Figure S3 (Supporting Information). All of the pairs highlighted in Figure S3 show an upward trend, especially evident for NK cells | TILS, ROS | PTT, and nectin-4 | ADCs.

**Figure 6:**
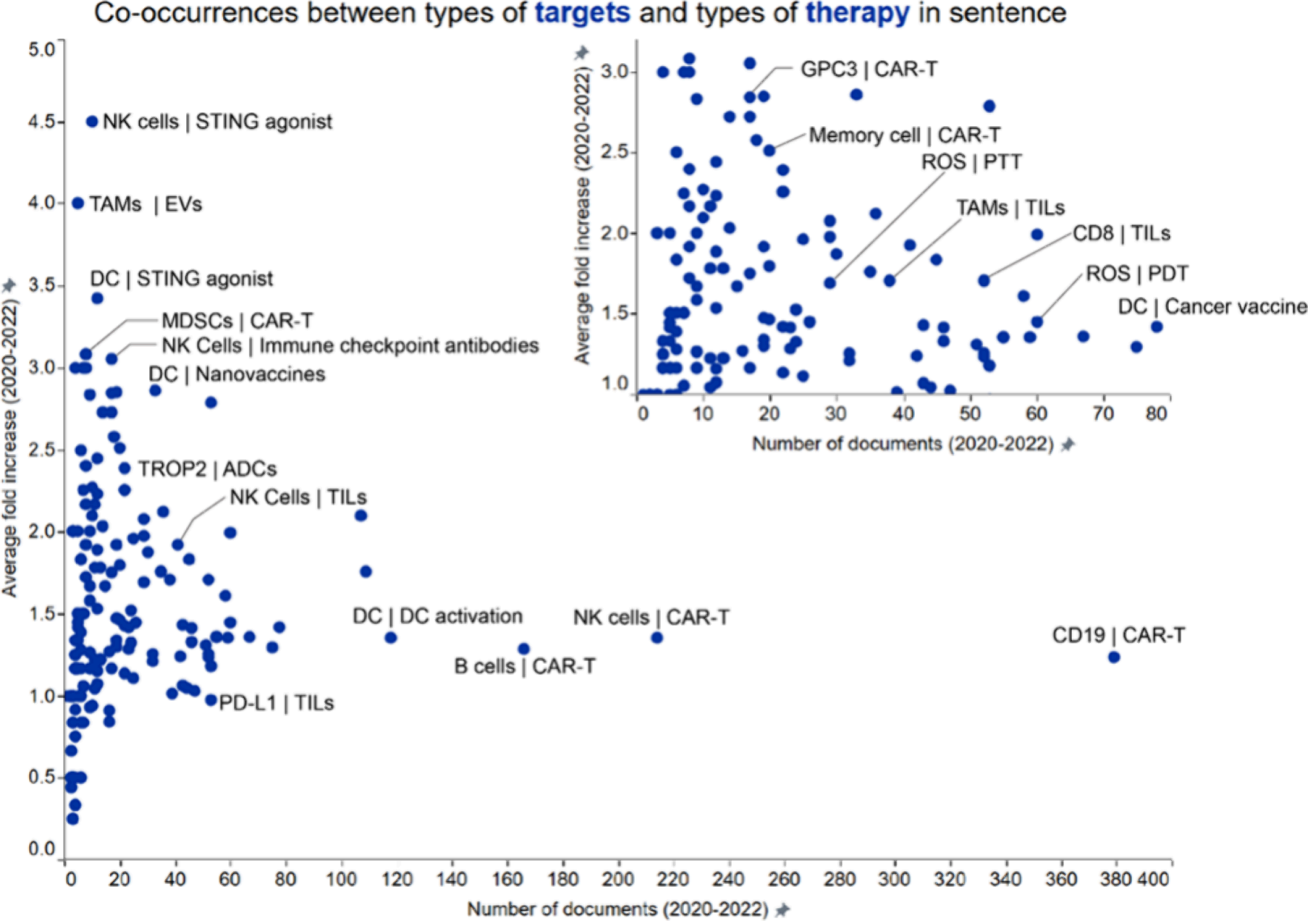
Co-occurrence analysis of emerging concepts in immuno-oncology focused on therapeutic targets and therapy types. Concepts pairs co-occurred within a sentence in the title or abstract of publications. Labels outline certain noteworthy combinations exhibiting either high number of documents (right part of the graph) or high growth rate (upper left). Shown inset is a graph focused on data clustered in the lower left corner of the bigger graph.

#### NK Cells | STING Agonist

Natural Killer (NK) cells are a type of lymphocyte that plays a crucial role in the innate immune response, particularly in recognizing and killing virally infected cells and tumor cells. In the context of immuno-oncology, NK cells are recognized for their ability to target and eliminate cancer cells without the need for prior sensitization to tumor antigens, unlike T cells. Their function is primarily regulated by a balance between activating and inhibitory signals received through various receptors on their surface.^338–341^

NK cells identify stressed cells, such as tumor cells, through the downregulation of major histocompatibility complex (MHC) class I molecules and the expression of stress-induced ligands. Upon recognition, NK cells release cytotoxic granules containing perforin and granzymes, leading to lysis of the target cells. NK cells can also mediate ADCC through the Fc receptor CD16, which binds to antibodies coating tumor cells, leading to targeted cell lysis. NK cells secrete cytokines like IFN-γ, which can modulate the TME, enhancing the adaptive immune response and inhibiting tumor growth.^338,342,343^

The stimulator of interferon genes (STING) pathway is a critical component of the innate immune response, particularly in the detection of cytosolic DNA from pathogens or damaged cells, which can occur in cancer. Activation of STING leads to the production of type I interferons and other cytokines, which can enhance the antitumor immune response by promoting the activation of dendritic cells, T cells, and NK cells.^344–346^

STING agonists activate the STING pathway, leading to the production of type I interferons, which enhance the activation and recruitment of various immune cells, including T cells and NK cells, to the tumor site. By activation of dendritic cells, STING agonists can enhance the presentation of tumor antigens to T cells, thereby promoting an adaptive immune response against the tumor. In addition to their immune-modulating effects, STING agonists can directly induce apoptosis in tumor cells.^346–350^

STING agonists are often studied in combination with other immunotherapies, such as ICIs (e.g., anti-PD-1/PD-L1), to enhance their efficacy by overcoming the immunosuppressive TME. The combination of therapies holds promise as they target different aspects of the immune system, potentially leading to synergistic effects in eliminating tumors.^351–353^

The high average fold increase (>4.5-fold) in the NK cell | STING agonist concept pair indicates growing interest from the scientific community in recent years (Figure 6 and Figure S3).

#### NK Cells | TILs

Natural killer (NK) cells are innate immune cells known for their ability to recognize and destroy tumor cells without prior sensitization.^354^ NK cells are distinguished from other immune cells by their ability to target cells that lack MHC class I molecules, which are often down-regulated in tumor cells as a mechanism to evade T cell-mediated immunity. NK cells express a range of activating and inhibitory receptors that regulate their function, making them crucial in the immune surveillance against cancer.^340^

TILs, particularly CD8+ cytotoxic T cells, recognize tumor-associated antigens presented by MHC class I molecules on the surface of cancer cells. This recognition leads to the activation and killing of the tumor cells. TILs secrete various cytokines, such as IFN-γ and TNF-α, which help in orchestrating a broader antitumor immune response and modifying the TME to make it less conducive to tumor growth. TILs can develop into memory T cells, providing long-term surveillance against tumor recurrence.^355–357^

Both NK cells and TILs play crucial roles in antitumor immunity, and combining these approaches could potentially synergistically enhance therapeutic outcomes. For example, NK cells can modulate the TME and facilitate T cell infiltration, while TILs can provide targeted adaptive immune responses. The combination of NK cell-based therapies with TIL therapies or checkpoint inhibitors represents a promising avenue for developing more effective cancer treatments.^358–360^

#### GPC3 | CAR-T

Glypican-3 (GPC3) is a cell surface proteoglycan that is part of the glypican family and involved in cell growth, differentiation, and migration. GPC3 is highly expressed in several cancers, most notably HCC, but is absent or minimally expressed in normal adult tissues, making it an attractive target for cancer immunotherapy.^361–364^

GPC3 is involved in the regulation of cell signaling pathways, such as Wnt/β-catenin, which are critical for tumor growth and development. Overexpression of GPC3 is associated with increased tumor proliferation and poor prognosis in cancers like HCC. The restricted expression of GPC3 to tumor cells, particularly in HCC, but not in most normal tissues, makes it an ideal target for immunotherapies that aim to minimize off-target effects.^361,362,365^

For GPC3, CAR-T cells are engineered to target GPC3-expressing tumor cells, especially in cancers like hepatocellular carcinoma.^366–368^ GPC3-targeted CAR-T therapies are being evaluated in clinical trials, particularly for the treatment of HCC. Innovations such as dual-targeting CAR-T cells (which target GPC3 and another tumor antigen), armored CAR-T cells (engineered to secrete cytokines or express checkpoint inhibitors), and combination therapies are being explored to enhance the therapeutic potential of GPC3-targeted CAR-T cells.^367,369^

GPC3 is a promising target in immuno-oncology due to its tumor-specific expression and role in tumorigenesis. CAR-T cell therapy targeting GPC3 offers a powerful approach to treating cancers like HCC. While challenges remain, ongoing research and clinical trials continue to refine these therapies, with the goal of improving outcomes for patients with GPC3-positive cancers.^364,370^

#### ROS | Photothermal Therapy (PTT)

Reactive oxygen species (ROS) are reactive molecules containing oxygen, such as superoxide (O_2_^•–^), hydrogen peroxide (H_2_O_2_), and hydroxyl radicals (•OH). These molecules are typically produced as byproducts of cellular metabolism, particularly within mitochondria. While ROS are essential for normal cellular functions like signaling and defense against pathogens, their dysregulation can contribute to cancer development and progression.^371–374^

At high levels, ROS can induce oxidative stress, leading to apoptosis (programmed cell death) or necrosis in cancer cells. This property makes ROS a target for therapies aimed at increasing ROS levels in tumors to induce cancer cell death. ROS can modulate the immune response, influencing the activity of various immune cells, including T cells and macrophages, which can either promote or inhibit tumor growth depending on the context.^375–377^

Photothermal therapy (PTT) is a minimally invasive treatment that involves the use of photosensitizers, such as nanoparticles, that can absorb near-infrared (NIR) light and convert it into heat. This localized heat can destroy cancer cells directly and also stimulate antitumor immune responses, making PTT a promising approach in immuno-oncology.^378,379^

When photosensitizers are exposed to NIR light, they generate heat, leading to hyperthermia (elevated temperature) in tumor tissue. This can cause direct thermal damage to cancer cells, leading to their destruction. The heat generated during PTT can cause the release of tumor antigens and danger-associated molecular patterns (DAMPs) from dying cancer cells.^380^ These molecules can enhance the presentation of tumor antigens to the immune system, promoting an antitumor immune response. PTT can alter the TME, making it more conducive to immune cell infiltration. This can enhance the effectiveness of other immunotherapies, such as ICIs, by making tumors more ″visible″ to the immune system.^381–384^

ROS and PTT are both emerging areas in immuno-oncology that offer unique mechanisms for targeting and destroying cancer cells while potentially enhancing the immune response against tumors. While challenges remain, these therapies represent promising avenues for developing more effective and less invasive cancer treatments, particularly when combined with other immunotherapeutic approaches.^385,386^

#### ROS | Sonodynamic Therapy (SDT)

Sonodynamic therapy (SDT) is a noninvasive treatment modality that uses low-intensity ultrasound in combination with sonosensitizers to generate ROS and induce selective cytotoxic effects in tumor cells. SDT has emerged as a promising approach in cancer therapy due to its ability to penetrate deep tissues and its potential to be combined with immunotherapies.

Sonosensitizers are compounds that preferentially accumulate in tumor tissues. When exposed to ultrasound, these sensitizers undergo a physical or chemical transformation, leading to the production of ROS, such as singlet oxygen (^1^O_2_), hydroxyl radicals (•OH), and superoxide anions (O_2_^•–^). The application of low-intensity ultrasound to the tumor site activates the sonosensitizers, leading to the localized generation of ROS within the TME. The resulting oxidative stress can cause direct damage to tumor cells, leading to cell death.

SDT is highly selective because the ROS generation and cytotoxic effects are confined to the area where ultrasound is applied and where the sonosensitizers are localized. This minimizes damage to the surrounding healthy tissues.

ROS and SDT are both important areas of focus in immuno-oncology. ROS play dual roles in cancer, contributing to both tumor progression and therapy-induced tumor cell death. Harnessing ROS through therapies such as SDT offers a promising avenue for selectively targeting and destroying cancer cells, especially when combined with other immunotherapeutic approaches. SDT, with its noninvasive nature and potential for deep tissue targeting, is an emerging modality that holds significant promise in the treatment of various cancers, particularly when combined with strategies that enhance the immune response.

#### Nectin-4 | ADCs

One of the most advanced ADCs targeting nectin-4 is enfortumab vedotin (EV), which is approved for the treatment of urothelial carcinoma, particularly in patients who have previously been treated with platinum-based chemotherapy and ICIs.^191,387–389^

Enfortumab vedotin consists of a monoclonal antibody targeting nectin-4, linked to the cytotoxic agent monomethyl auristatin E (MMAE), a potent microtubule-disrupting agent. Upon binding to nectin-4 on the surface of tumor cells, the ADC is internalized, and MMAE is released, leading to the disruption of the microtubule network and subsequent cell death.^189,390,391^

Enfortumab vedotin has shown significant clinical efficacy in patients with advanced urothelial carcinoma, with response rates and survival benefits leading to its approval by regulatory agencies. It is currently being investigated in other cancers where nectin-4 is overexpressed.^389,392^

Combining nectin-4-targeting ADCs with other therapies, such as ICIs or other targeted agents, is an area of active research. The goal is to enhance efficacy, overcome resistance, and improve patient outcomes.^393,394^

#### TROP2 | ADCs

Trophoblast cell-surface antigen 2 (TROP2), also known as tumor-associated calcium signal transducer 2 (TACSTD2), is a cell surface glycoprotein that plays a role in cell signaling, proliferation, and survival. TROP2 is expressed at low levels in normal tissues but is overexpressed in a variety of cancers including breast, lung, colorectal, pancreatic, and prostate cancers. This overexpression makes TROP2 a valuable target in cancer therapy, particularly for ADCs.^395–397^

TROP2 is commonly overexpressed in many epithelial cancers, where it is associated with aggressive tumor behavior, including enhanced proliferation, invasion, and metastasis. High TROP2 expression often correlates with a poor prognosis. TROP2 can activate signaling pathways that promote cancer cell growth and survival, such as the PI3K/AKT and ERK/MAPK pathways. It is also involved in modulating cell adhesion, which can affect cancer metastasis.^398–400^

Sacituzumab govitecan is a well-known ADC that targets TROP2.^401–403^ It is approved for the treatment of triple-negative breast cancer (TNBC) and urothelial carcinoma. The ADC consists of a monoclonal antibody against TROP2 linked to SN-38, a potent topoisomerase I inhibitor that induces DNA damage and cell death.^404,405^ After binding to TROP2 on the surface of cancer cells, sacituzumab govitecan is internalized and SN-38 is released inside the cell. SN-38 inhibits topoisomerase I, leading to DNA strand breaks, which ultimately trigger apoptosis in cancer cells. Sacituzumab govitecan has shown significant clinical activity in patients with heavily pretreated TNBC and metastatic urothelial carcinoma, demonstrating substantial improvements in progression-free survival (PFS) and overall survival (OS) compared to standard therapies.^406–408^

In summary, the rapid evolution of immuno-oncology has revealed intricate relationships between molecular targets and therapeutic modalities, with specific pairings demonstrating synergistic potential. Table 4 summarizes representative combinations of targets and therapies along with their synergistic mechanisms and clinical prospects.

**Table 4:** Summary of Representative Types of Targets and Types of Therapy Combinations with Their Synergistic Mechanisms and Clinical Prospects[Table-fn t4fn2]

**target**	**therapy**	**synergistic mechanism**	**clinical prospects**
NK cells	STING agonists	STING activation enhances NK cytotoxicity via IFN-β; promotes DC cross-presentation	phase I trials in melanoma/lymphoma (e.g., NCT04144140)
NK cells	TILs	NKs secrete IL-15/IFN-γ to boost TIL expansion; TILs provide antigen-specific killing	phase I in NSCLC (e.g., NCT04211675)
GPC3	CAR-T	GPC3 overexpression in HCC enables targeted killing; armored CARs resist TME suppression	phase I/II in HCC (e.g., NCT03980288, 28% ORR)
ROS	photothermal therapy (PTT)	PTT generates localized ROS, inducing immunogenic cell death (calreticulin/HMGB1 exposure)	phase I in breast cancer with anti-PD-1
ROS	sonodynamic therapy (SDT)	ultrasound-triggered ROS activates DCs and enhances tumor antigen presentation	preclinical success in glioblastoma; human trials pending
nectin-4	ADCs (enfortumab vedotin)	ADC internalization releases payload, killing tumor cells and inducing immunogenic death	FDA-approved for bladder cancer; combo trials with pembrolizumab (EV-103)
TROP2	ADCs (sacituzumab govitecan)	TROP2-directed payload delivery upregulates MHC-I, sensitizing tumors to T cells	FDA-approved in TNBC; Phase III in NSCLC (NCT04656652)

a ORR, overall response rate.

Besides the intergroup co-occurrences discussed above, we also identified concept pairs co-occurring within a group (therapeutic targets, cancer types, biomarkers, therapy types) shown in Figures S4–S7 (Supporting Information).

## Conclusions

Since its inception, the continued development of the field of immuno-oncology remains unmistakable, exemplified by the 2018 Nobel Prize in Physiology or Medicine being awarded to James Allison and Tasuku Honjo for their work related to CTLA-4.^409^ Being able to analyze a dynamic, fast-growing field such as immuno-oncology in order to identify emerging areas of interest remains a challenge. Utilizing an NLP-based approach, we have previously reported more than 300 emerging concepts in immuno-oncology, ranging from therapeutic targets, biomarkers, and cancer types to therapy types, among others.^1^ In this report, we extend our analysis to provide a greater context for the previously identified emerging concepts. In doing so, we have identified concept pairs that tend to co-occur with each other, uncovering possible new connections between merging concepts. This contextual information may be useful in directing future research efforts in this valuable direction.

Co-occurrence methods^410–419^ represent a fundamental approach in text data analysis, focusing on the statistical relationships between words that appear together within a defined context, and have become increasingly important in natural language processing (NLP), information retrieval, and computational linguistics. Co-occurrence analysis is grounded in the distributional hypothesis, articulated by Firth’s famous statement that ″you shall know a word by the company it keeps”. This principle suggests that words appearing in similar contexts tend to have related meanings. Building on this foundation, co-occurrence methods quantify the relationships between lexical items based on their patterns of appearance in textual data.

Our analysis in the context of immuno-oncology indicates that co-occurring concept pairs tend to be spread across two extremes—those with high growth in publications over the past few years but an overall low number of publications, and those with relatively slower growth in publications but an overall higher number of publications. The former represent potentially emerging concept pairs, while the latter represent concept pairs that are perhaps better studied and/or reported, and identifying both types of emerging concept pairs has merit. Our focus was limited to the following four concept pairs: biomarkers and cancer type, therapeutic targets and cancer type, therapeutic targets and therapy types, and therapy and cancer types. While we performed co-occurrence analysis for both criteria, co-occurrence of terms in sentences and in abstracts, we chose to prioritize the in-sentence co-occurrence as it was much less likely to result from coincidence and implied a stronger relationship between the terms.

While newer deep learning approaches have expanded the toolkit for text analysis, co-occurrence statistics continue to offer computational efficiency, interpretability, and solid theoretical grounding. Compared to traditional statistical analysis and machine learning methods, NLP and co-occurrence approaches offer better handling of text’s inherent complexity but may require more specialized implementation and careful interpretation of results. The future of co-occurrence analysis likely lies in hybrid approaches that combine traditional statistical methods with neural techniques, leveraging the strengths of both paradigms to address increasingly complex text analysis challenges. Co-occurrence methods remain a cornerstone of text data analysis, providing robust frameworks for understanding semantic relationships in a language.

Continued refinement and development of analytical methods such as these are important to identify emerging concepts in various fields. We hope our results from this study in conjunction with our previously published results^1^ can provide insights into the highly interconnected and complex landscape of immuno-oncology.

## Supplementary Material



## Notes

Detailed description of co-occurrence methodology, review of traditional statistical approaches, brief description of co-occurrence analysis and their applications, Figures S1–S3 showing yearly trends for various emerging pairs, and Figures S4–S7 showing in sentence co-occurrences between emerging topics in various categories: types of therapeutic targets, therapy type, biomarker, and types of cancer (PDF)

## References

[1] Iyer K. A., Ivanov J., Tenchov R., Ralhan K., Rodriguez Y., Sasso J. M., Scott S., Zhou Q. A. (2024). Emerging targets
and therapeutics in immuno-oncology: Insights from landscape analysis. J. Med. Chem..

[2] Sanmamed M. F., Chen L. (2018). A paradigm shift in
cancer immunotherapy: From enhancement to normalization. Cell.

[3] Zhang Y., Zhang Z. (2020). The history and advances in cancer
immunotherapy: Understanding the
characteristics of tumor-infiltrating immune cells and their therapeutic
implications. Cellular & Molecular Immunology.

[4] Lin M. J., Svensson-Arvelund J., Lubitz G. S., Marabelle A., Melero I., Brown B. D., Brody J. D. (2022). Cancer vaccines:
The next immunotherapy frontier. Nature Cancer.

[5] Tan S., Day D., Nicholls S. J., Segelov E. (2022). Immune checkpoint inhibitor therapy
in oncology: Current uses and future directions: JACC: CardioOncology
state-of-the-art review. JACC: CardioOncology.

[6] Cappell K. M., Kochenderfer J. N. (2023). Long-term outcomes following CAR T cell therapy: what
we know so far. Nature Reviews Clinical Oncology.

[7] Miliotou A. N., Papadopoulou L. C. (2018). CAR T-cell therapy: A new era in cancer immunotherapy. Curr. Pharm. Biotechnol..

[8] CAS Content Collection. https://www.cas.org/about/cas-content (accessed 2024 Mar 31, 2024).

[9] Ivanov, J. ; Lipkus, A. ; Chen, H. ; Aultman, C. ; Iyer, K. ; Tenchov, R. ; Zhou, Q. [Pre-print] Emerging topics – bibliometric-based methodology. ChemRxiv 2023. DOI: 10.26434/chemrxiv-2023-7d37m.

[10] Liu Z., Gao L., Cheng L., Lv G., Sun B., Wang G., Tang Q. (2023). The roles of N6-methyladenosine
and its target regulatory noncoding
RNAs in tumors: classification, mechanisms, and potential therapeutic
implications. Experimental & Molecular Medicine.

[11] Gu C., Shi X., Dai C., Shen F., Rocco G., Chen J., Huang Z., Chen C., He C., Huang T., Chen C. (2020). RNA m^6^A modification in cancers: Molecular mechanisms
and potential clinical applications. Innovation.

[12] Zheng S., Han H., Lin S. (2022). N­(6)-methyladenosine
(m­(6)­A) RNA modification in tumor
immunity. Cancer Biology & Medicine.

[13] Diao M.-N., Zhang X.-J., Zhang Y.-F. (2023). The critical roles of m6A RNA methylation
in lung cancer: from mechanism to prognosis and therapy. Br. J. Cancer.

[14] Wu Y., Li H., Huang Y., Chen Q. (2023). Silencing of m^6^A methyltransferase
KIAA1429 suppresses the progression of non-small cell lung cancer
by promoting the p53 signaling pathway and ferroptosis. Am. J. Cancer Res..

[15] Yu M., Ji W., Yang X., Tian K., Ma X., Yu S., Chen L., Zhao X. (2023). The role of m6A demethylases in lung
cancer: diagnostic and therapeutic implications. Front. Immunol..

[16] Zhang Q., Xu K. (2023). The role of regulators of RNA m6A
methylation in lung cancer. Genes & Diseases.

[17] Qiu F.-S., He J.-Q., Zhong Y.-S., Guo M.-Y., Yu C.-H. (2022). Implications
of m6A methylation and microbiota interaction in non-small cell lung
cancer: From basics to therapeutics. Front.
Cell. Infect. Microbiol..

[18] Dong B., Wu C., Li S.-H., Huang L., Zhang C., Wu B., Sheng Y., Liu Y., Ye G., Qi Y. (2021). Correlation
of m6A methylation with immune infiltrates and poor prognosis in non-small
cell lung cancer via a comprehensive analysis of RNA expression profiles. Annals of Translational Medicine.

[19] Ye W., Huang T. (2022). Correlation analysis
of m6A-modified regulators with immune microenvironment
infiltrating cells in lung adenocarcinoma. PLoS
One.

[20] Zhuang H., Yu B., Tao D., Xu X., Xu Y., Wang J., Jiao Y., Wang L. (2023). The role of m6A methylation
in therapy
resistance in cancer. Molecular Cancer.

[21] Xue H., Ma Y., Guan K., Zhou Y., Liu Y., Cao F., Kang X. (2024). The role of
m6A methylation in targeted therapy resistance in lung
cancer. American Journal of Cancer Research.

[22] Zhang Z., Liu F., Chen W., Liao Z., Zhang W., Zhang B., Liang H., Chu L., Zhang Z. (2022). The importance of N6-methyladenosine
modification in tumor immunity and immunotherapy. Experimental Hematology & Oncology.

[23] Gan L., Zhao Y., Fu Y., Chen Q. (2023). The potential role
of m6A modifications on immune cells and immunotherapy. Biomedicine & Pharmacotherapy.

[24] Li X., Ma S., Deng Y., Yi P., Yu J. (2022). Targeting the RNA m6A
modification for cancer immunotherapy. Molecular
Cancer.

[25] Wang Z., Zhou J., Zhang H., Ge L., Li J., Wang H. (2023). RNA m(6) A methylation in cancer. Molecular
Oncology.

[26] Xu L., Zhou L., Yan C., Li L. (2022). Emerging role of N6-methyladenosine
RNA methylation in lung diseases. Experimental
biology and medicine (Maywood, NJ).

[27] Chen M., Wong C. M. (2020). The emerging roles
of N6-methyladenosine (m6A) deregulation
in liver carcinogenesis. Molecular Cancer.

[28] Ma H., Hong Y., Xu Z., Weng Z., Yang Y., Jin D., Chen Z., Yue J., Zhou X., Xu Z. (2024). N6-methyladenosine (m6A)
modification in hepatocellular carcinoma. Biomedicine
& Pharmacotherapy.

[29] Wang S., Gao S., Ye W., Li Y., Luan J., Lv X. (2023). The emerging
importance role of m6A modification in liver disease. Biomedicine & Pharmacotherapy.

[30] Lin Z., Niu Y., Wan A., Chen D., Liang H., Chen X., Sun L., Zhan S., Chen L., Cheng C. (2020). RNA m^6^A methylation regulates sorafenib resistance in liver cancer
through FOXO3-mediated autophagy. EMBO Journal.

[31] Sivasudhan E., Blake N., Lu Z.-L., Meng J., Rong R. (2021). Dynamics of
m6A RNA methylome on the hallmarks of hepatocellular carcinoma. Front. Cell Dev. Biol..

[32] Qu N., Bo X., Li B., Ma L., Wang F., Zheng Q., Xiao X., Huang F., Shi Y., Zhang X. (2021). Role of N6-methyladenosine
(m6A) methylation regulators in hepatocellular carcinoma. Front. Oncol..

[33] Ma W., Wu T. (2022). RNA m6A modification in liver biology and its implication
in hepatic
diseases and carcinogenesis. American Journal
of Physiology Cell Physiology.

[34] Fang Z., Mei W., Qu C., Lu J., Shang L., Cao F., Li F. (2022). Role of m6A writers,
erasers and readers in cancer. Experimental
Hematology & Oncology.

[35] Petri B. J., Klinge C. M. (2023). m6A readers, writers, erasers, and
the m6A epitranscriptome
in breast cancer. J. Mol. Endocrinol..

[36] Wen T., Li T., Xu Y., Zhang Y., Pan H., Wang Y. (2023). The role of
m6A epigenetic modifications in tumor coding and non-coding RNA processing. Cell Communication and Signaling.

[37] Suphakhong K., Terashima M., Wanna-udom S., Takatsuka R., Ishimura A., Takino T., Suzuki T. (2022). m6A RNA methylation
regulates the transcription factors JUN and JUNB in TGF-β-induced
epithelial-mesenchymal transition of lung cancer cells. J. Biol. Chem..

[38] Wang C., Danli M., Yu H., Zhuo Z., Ye Z. (2023). N6-methyladenosine
(m6A) as a regulator of carcinogenesis and drug resistance by targeting
epithelial-mesenchymal transition and cancer stem cells. Heliyon.

[39] Clevers H. (2016). Cancer therapy:
Defining stemness. Nature.

[40] Qin S., Mao Y., Wang H., Duan Y., Zhao L. (2021). The interplay between
m6A modification and non-coding RNA in cancer stemness modulation:
mechanisms, signaling pathways, and clinical implications. International Journal of Biological Sciences.

[41] Jiang X., Liu B., Nie Z., Duan L., Xiong Q., Jin Z., Yang C., Chen Y. (2021). The role of m6A modification in the
biological functions and diseases. Signal Transduction
and Targeted Therapy.

[42] Shen S., Yan J., Zhang Y., Dong Z., Xing J., He Y. (2021). N6-methyladenosine
(m6A)-mediated messenger RNA signatures and the tumor immune microenvironment
can predict the prognosis of hepatocellular carcinoma. Annals of Translational Medicine.

[43] Li C., Zhu M., Wang J., Wu H., Liu Y., Huang D. (2023). Role of m6A
modification in immune microenvironment of digestive system tumors. Biomedicine & Pharmacotherapy.

[44] Yin M., Shen J., Yu S., Fei J., Zhu X., Zhao J., Zhai L., Sadhukhan A., Zhou J. (2019). Tumor-associated macrophages (TAMs): A critical activator In ovarian
cancer metastasis. OncoTargets and Therapy.

[45] Schweer D., McAtee A., Neupane K., Richards C., Ueland F., Kolesar J. (2022). Tumor-associated macrophages
and ovarian cancer: Implications
for therapy. Cancers.

[46] Truxova I., Cibula D., Spisek R., Fucikova J. (2023). Targeting tumor-associated
macrophages for successful immunotherapy of ovarian carcinoma. Journal for ImmunoTherapy of Cancer.

[47] Glass E. B., Hoover A. A., Bullock K. K., Madden M. Z., Reinfeld B. I., Harris W., Parker D., Hufnagel D. H., Crispens M. A., Khabele D. (2022). Stimulating
TAM-mediated anti-tumor immunity with mannose-decorated
nanoparticles in ovarian cancer. BMC Cancer.

[48] Brauneck F., Oliveira-Ferrer L., Muschhammer J., Sturmheit T., Ackermann C., Haag F., Schulze zur Wiesch J., Ding Y., Qi M., Hell L. (2023). Immunosuppressive
M2 TAMs represent a promising target population to enhance phagocytosis
of ovarian cancer cells in vitro. Front. Immunol..

[49] Steitz A. M., Steffes A., Finkernagel F., Unger A., Sommerfeld L., Jansen J. M., Wagner U., Graumann J., Müller R., Reinartz S. (2020). Tumor-associated macrophages
promote ovarian cancer
cell migration by secreting transforming growth factor beta induced
(TGFBI) and tenascin C. Cell Death & Disease.

[50] Pan Y., Yu Y., Wang X., Zhang T. (2020). Tumor-associated macrophages in tumor
immunity. Front. Immunol..

[51] Boutilier A. J., Elsawa S. F. (2021). Macrophage polarization
states in the tumor microenvironment. Int. J.
Mol. Sci..

[52] Hagemann T., Wilson J., Burke F., Kulbe H., Li N. F., Plüddemann A., Charles K., Gordon S., Balkwill F. R. (2006). Ovarian
cancer cells polarize macrophages toward a tumor-associated phenotype1. J. Immunol..

[53] Li M., He L., Zhu J., Zhang P., Liang S. (2022). Targeting tumor-associated
macrophages for cancer treatment. Cell Biosci..

[54] Huang R., Kang T., Chen S. (2024). The role of
tumor-associated macrophages
in tumor immune evasion. Journal of Cancer Research
and Clinical Oncology.

[55] Bied M., Ho W. W., Ginhoux F., Blériot C. (2023). Roles of macrophages
in tumor development: A spatiotemporal perspective. Cellular & Molecular Immunology.

[56] Osińska I., Popko K., Demkow U. (2014). Perforin:
An important player in
immune response. Cent. Eur. J. Immunol..

[57] Cao X., Cai S. F., Fehniger T. A., Song J., Collins L. I., Piwnica-Worms D. R., Ley T. J. (2007). Granzyme B and perforin are important
for regulatory T cell-mediated suppression of tumor clearance. Immunity.

[58] Governa V., Trella E., Mele V., Tornillo L., Amicarella F., Cremonesi E., Muraro M. G., Xu H., Droeser R., Däster S. R. (2017). The interplay between
neutrophils and
CD8+ T cells improves survival in human colorectal cancer. Clin. Cancer Res..

[59] Glaire M. A., Domingo E., Sveen A., Bruun J., Nesbakken A., Nicholson G., Novelli M., Lawson K., Oukrif D., Kildal W. (2019). Tumour-infiltrating CD8+ lymphocytes and colorectal
cancer recurrence by tumour and nodal stage. Br. J. Cancer.

[60] Kasurinen J., Hagström J., Kaprio T., Beilmann-Lehtonen I., Haglund C., Böckelman C. (2022). Tumor-associated CD3- and CD8-positive
immune cells in colorectal cancer: The additional prognostic value
of CD8 + -to-CD3 + ratio remains debatable. Tumor Biology.

[61] Saleh R., Sasidharan Nair V., Toor S. M., Taha R. Z., Murshed K., Al-Dhaheri M., Khawar M., Petkar M. A., Abu Nada M., Al-Ejeh F., Elkord E. (2020). Differential gene expression of tumor-infiltrating
CD8^+^ T cells in advanced versus early-stage colorectal
cancer and identification of a gene signature of poor prognosis. Journal for ImmunoTherapy of Cancer.

[62] Xie Q., Ding J., Chen Y. (2021). Role of CD8­(+)
T lymphocyte cells:
Interplay with stromal cells in tumor microenvironment. Acta Pharm. Sin. B.

[63] Zheng Z., Wieder T., Mauerer B., Schäfer L., Kesselring R., Braumüller H. (2023). T cells in colorectal cancer: Unravelling
the function of different T cell subsets in the tumor microenvironment. Int. J. Mol. Sci..

[64] Piroozkhah M., Gholinezhad Y., Piroozkhah M., Shams E., Nazemalhosseini-Mojarad E. (2023). The molecular
mechanism of actions and clinical utilities of tumor infiltrating
lymphocytes in gastrointestinal cancers: a comprehensive review and
future prospects toward personalized medicine. Front. Immunol..

[65] Brummel K., Eerkens A. L., de Bruyn M., Nijman H. W. (2023). Tumour-infiltrating
lymphocytes: From prognosis to treatment selection. Br. J. Cancer.

[66] Raskov H., Orhan A., Christensen J. P., Gögenur I. (2021). Cytotoxic
CD8­(+) T cells in cancer and cancer immunotherapy. Br. J. Cancer.

[67] Emens L. A., Romero P. J., Anderson A. C., Bruno T. C., Capitini C. M., Collyar D., Gulley J. L., Hwu P., Posey A. D., Silk A. W., Wargo J. A. (2024). Challenges and opportunities
in cancer
immunotherapy: A society for immunotherapy of cancer (SITC) strategic
vision. Journal for ImmunoTherapy of Cancer.

[68] Gordon B., Gadi V. K. (2020). The Role of the Tumor Microenvironment
in Developing
Successful Therapeutic and Secondary Prophylactic Breast Cancer Vaccines. Vaccines.

[69] Zhang X., Yang Z., An Y., Liu Y., Wei Q., Xu F., Yao H., Zhang Z. (2022). Clinical benefits of PD-1/PD-L1 inhibitors
in patients with metastatic colorectal cancer: A systematic review
and meta-analysis. World Journal of Surgical
Oncology.

[70] Yang Z., Wu G., Zhang X., Gao J., Meng C., Liu Y., Wei Q., Sun L., Wei P., Bai Z. (2022). Current
progress and future perspectives of neoadjuvant anti-PD-1/PD-L1 therapy
for colorectal cancer. Front. Immunol..

[71] Cercek A., Lumish M., Sinopoli J., Weiss J., Shia J., Lamendola-Essel M., El Dika I. H., Segal N., Shcherba M., Sugarman R. (2022). PD-1 blockade in mismatch repair–deficient,
locally advanced rectal cancer. New England
Journal of Medicine.

[72] Li Y., Du Y., Xue C., Wu P., Du N., Zhu G., Xu H., Zhu Z. (2022). Efficacy and
safety of anti-PD-1/PD-L1 therapy in the
treatment of advanced colorectal cancer: a meta-analysis. BMC Gastroenterology.

[73] Lin K. X., Istl A. C., Quan D., Skaro A., Tang E., Zheng X. (2023). PD-1 and PD-L1 inhibitors in cold
colorectal cancer: challenges and
strategies. Cancer Immunology, Immunotherapy.

[74] Chen X., Chen L.-J., Peng X.-F., Deng L., Wang Y., Li J.-J., Guo D.-L., Niu X.-H. (2024). Anti-PD-1/PD-L1
therapy for colorectal cancer: Clinical implications and future considerations. Translational Oncology.

[75] Ghosh C., Luong G., Sun Y. (2021). A snapshot of the PD-1/PD-L1
pathway. J. Cancer.

[76] Han Y., Liu D., Li L. (2020). PD-1/PD-L1 pathway: Current researches in cancer. Am. J. Cancer Res..

[77] Wang L., Guo W., Guo Z., Yu J., Tan J., Simons D. L., Hu K., Liu X., Zhou Q., Zheng Y. (2024). PD-L1-expressing
tumor-associated macrophages are immunostimulatory and associate with
good clinical outcome in human breast cancer. Cell Rep. Med..

[78] Petty A. J., Dai R., Lapalombella R., Baiocchi R. A., Benson D. M., Li Z., Huang X., Yang Y. (2021). Hedgehog-induced PD-L1 on tumor-associated
macrophages is critical for suppression of tumor-infiltrating CD8+
T cell function. JCI Insight.

[79] Jiang Y., Chen M., Nie H., Yuan Y. (2019). PD-1 and PD-L1 in cancer
immunotherapy: Clinical implications and future considerations. Human Vaccines & Immunotherapies.

[80] Pu Y., Ji Q. (2022). Tumor-associated macrophages
regulate PD-1/PD-L1 immunosuppression. Front.
Immunol..

[81] Sahin I. H., Akce M., Alese O., Shaib W., Lesinski G. B., El-Rayes B., Wu C. (2019). Immune checkpoint inhibitors for
the treatment of MSI-H/MMR-D colorectal cancer and a perspective on
resistance mechanisms. Br. J. Cancer.

[82] Yan S., Wang W., Feng Z., Xue J., Liang W., Wu X., Tan Z., Zhang X., Zhang S., Li X., Zhang C. (2024). Immune checkpoint
inhibitors in colorectal cancer: Limitation and
challenges. Front. Immunol..

[83] Sharma S., Singh N., Turk A. A., Wan I., Guttikonda A., Dong J. L., Zhang X., Opyrchal M. (2024). Molecular
insights
into clinical trials for immune checkpoint inhibitors in colorectal
cancer: Unravelling challenges and future directions. World Journal of Gastroenterology.

[84] Gaynor N., Crown J., Collins D. M. (2022). Immune
checkpoint inhibitors: Key
trials and an emerging role in breast cancer. Seminars in Cancer Biology.

[85] Shojaie L., Ali M., Iorga A., Dara L. (2021). Mechanisms of immune checkpoint inhibitor-mediated
liver injury. Acta Pharmaceutica Sinica B.

[86] Keir M. E., Butte M. J., Freeman G. J., Sharpe A. H. (2008). PD-1 and its ligands
in tolerance and immunity. Annu. Rev. Immunol..

[87] Parvez A., Choudhary F., Mudgal P., Khan R., Qureshi K. A., Farooqi H., Aspatwar A. (2023). PD-1 and PD-L1: Architects of immune
symphony and immunotherapy breakthroughs in cancer treatment. Front. Immunol..

[88] Lin X., Kang K., Chen P., Zeng Z., Li G., Xiong W., Yi M., Xiang B. (2024). Regulatory mechanisms
of PD-1/PD-L1 in cancers. Molecular Cancer.

[89] D’Alessio A., Rimassa L., Cortellini A., Pinato D. J. (2021). PD-1 blockade for
hepatocellular carcinoma: Current research and future prospects. Journal of Hepatocellular Carcinoma.

[90] Uhlig J., Stein S., Kim H. S. (2022). PD-1 targeted
immunotherapy for advanced
hepatocellular carcinoma: current utilization and outcomes in the
USA. Future Oncology.

[91] Yin X., Wu T., Lan Y., Yang W. (2022). Current progress of immune checkpoint
inhibitors in the treatment of advanced hepatocellular carcinoma. Biosci. Rep..

[92] Li Q., Han J., Yang Y., Chen Y. (2022). PD-1/PD-L1 checkpoint inhibitors
in advanced hepatocellular carcinoma immunotherapy. Front. Immunol..

[93] Chen C., Li Z., Xiong X., Yao A., Wang S., Liu X., Liu X., Wang J. (2024). Intraperitoneal
PD-1 monoclonal antibody for the treatment
of advanced primary liver cancer with malignant ascites: a single-arm,
single-center, phase Ib trial. ESMO Open.

[94] Shiravand Y., Khodadadi F., Kashani S. M. A., Hosseini-Fard S. R., Hosseini S., Sadeghirad H., Ladwa R., O’Byrne K., Kulasinghe A. (2022). Immune checkpoint
inhibitors in cancer therapy. Current Oncology.

[95] Sung H., Ferlay J., Siegel R. L., Laversanne M., Soerjomataram I., Jemal A., Bray F. (2021). Global Cancer Statistics
2020: GLOBOCAN estimates of incidence and mortality worldwide for
36 cancers in 185 countries. CA: A Cancer Journal
for Clinicians.

[96] Morgan E., Arnold M., Camargo M. C., Gini A., Kunzmann A. T., Matsuda T., Meheus F., Verhoeven R. H. A., Vignat J., Laversanne M. (2022). The current and future
incidence and mortality of gastric cancer in 185 countries, 2020–40:
A population-based modelling study. eClinicalMedicine.

[97] Huo G., Liu W., Chen P. (2023). Efficacy of
PD-1/PD-L1 inhibitors in gastric or gastro-oesophageal
junction cancer based on clinical characteristics: a meta-analysis. BMC Cancer.

[98] Mastracci L., Grillo F., Parente P., Gullo I., Campora M., Angerilli V., Rossi C., Sacramento M. L., Pennelli G., Vanoli A., Fassan M. (2022). PD-L1 evaluation in
the gastrointestinal tract: from biological rationale to its clinical
application. Pathologica.

[99] Fiocco, C. ; Spencer, K. Defining PD-L1 expression in gastric cancer: How positive is CPS for finding and treating the right patients? 2023. https://dailynews.ascopubs.org/do/defining-pd-l1-expression-gastric-cancer-positive-cps-finding-and-treating-right (accessed Aug 14, 2024).

[100] Chen X. J., Wei C. Z., Lin J., Zhang R. P., Chen G. M., Li Y. F., Nie R. C., Chen Y. M. (2023). Prognostic
significance of PD-L1 expression in gastric cancer patients with peritoneal
metastasis. Biomedicines.

[101] Liu X., Choi M. G., Kim K., Kim K.-M., Kim S. T., Park S. H., Cristescu R., Peter S., Lee J. (2020). High PD-L1
expression in gastric cancer (GC) patients and correlation with molecular
features. Pathology - Research and Practice.

[102] Xie T., Zhang Z., Zhang X., Qi C., Shen L., Peng Z. (2021). Appropriate PD-L1 cutoff value for gastric cancer immunotherapy:
A systematic review and meta-analysis. Front.
Oncol..

[103] Shin K., Kim J., Park S. J., Lee M. A., Park J. M., Choi M.-G., Kang D., Song K. Y., Lee H. H., Seo H. S. (2023). Prognostic
value of
soluble PD-L1 and exosomal PD-L1 in advanced gastric cancer patients
receiving systemic chemotherapy. Sci. Rep..

[104] Zhao J., Xia Y. (2020). Targeting HER2 alterations
in non–small-cell
lung cancer: A comprehensive review. JCO Precision
Oncology.

[105] Uy N. F., Merkhofer C. M., Baik C. S. (2022). HER2 in non-small
cell lung cancer: A review of emerging therapies. Cancers.

[106] Yu X., Ji X., Su C. (2022). HER2-altered non-small cell lung
cancer: Biology, clinicopathologic features, and emerging therapies. Front. Oncol..

[107] Vathiotis I. A., Bafaloukos D., Syrigos K. N., Samonis G. (2023). Evolving treatment
landscape of HER2-mutant non-small cell lung cancer: Trastuzumab deruxtecan
and beyond. Cancers.

[108] Yu Y., Yang Y., Li H., Fan Y. (2023). Targeting HER2 alterations
in non-small cell lung cancer: Therapeutic breakthrough and challenges. Cancer Treatment Reviews.

[109] Lee Y., Lee B., Choi Y.-L., Kang D.-W., Han J. (2024). Clinicopathologic
and molecular characteristics of HER2 (ERBB2)-altered non-small cell
lung cancer: Implications for precision medicine. Mod. Pathol..

[110] Ferrari G., Del Rio B., Novello S., Passiglia F. (2024). HER2-altered
non-small cell lung cancer: A journey from current approaches to emerging
strategies. Cancers.

[111] Kato Y., Udagawa H., Matsumoto S., Izumi H., Ohe Y., Kato T., Nishino K., Miyamoto S., Kawashima Y., Chikamori K. (2024). Efficacy of immunotherapy in first-line treatment for non-small cell
lung cancer with HER2 mutation: Results from LC-SCRUM-Asia. J. Clin. Oncol..

[112] Zhang J., Han W., Guo J., Zhang C., Cao L., Peng L., Han X., Wang Z. (2024). Efficacy of immunotherapy
in HER2-mutated non-small cell lung cancer: A single-arm meta-analysis. Journal of Cancer Research and Clinical Oncology.

[113] DeMatteo R., Goldman D. A., Lin S. T., Buonocore D. J., Gao J., Chang J. C., Rekhtman N., Offin M., Yu H. A., Isbell J. M. (2022). Clinical
outcomes of immune checkpoint inhibitors
in HER2-amplified non-small cell lung cancers. J. Clin. Oncol..

[114] First drug targeting HER2-mutant non-small cell lung cancer approved by FDA. 2022. https://www.mskcc.org/news/first-drug-targeting-her2-mutant-non-small-cell-lung-cancer-approved-by-fda (accessed Aug 15, 2024).

[115] Wire, Why Business Enhertu approved in the US for patients with HER2-positive metastatic breast cancer treated with a prior anti-HER2-based regimen. 2022. https://www.astrazeneca.com/media-centre/press-releases/2022/enhertu-approved-in-us-for-2l-her2-positive-breast-cancer.html# (accessed Aug 15, 2024).

[116] Targeting HER2 in cancer: The story of zongertinib. https://www.boehringer-ingelheim.com/science-innovation/human-health-innovation/fostering-science/targeting-her2-cancer-story (accessed Aug 15, 2024).

[117] Haffner I., Schierle K., Raimúndez E., Geier B., Maier D., Hasenauer J., Luber B., Walch A., Kolbe K., Knorrenschild J. R. (2021). HER2 expression, test deviations, and their impact on survival in
metastatic gastric cancer: Results from the prospective multicenter
VARIANZ study. J. Clin. Oncol..

[118] Kaito A., Kuwata T., Tokunaga M., Shitara K., Sato R., Akimoto T., Kinoshita T. (2019). HER2 heterogeneity
is a poor prognosticator for HER2-positive gastric cancer. World Journal of Clinical Cases.

[119] Iqbal N., Iqbal N. (2014). Human epidermal growth
factor receptor
2 (HER2) in cancers: Overexpression and therapeutic implications. Mol. Biol. Int..

[120] Rubin I., Yarden Y. (2001). The basic biology of
HER2. Annals of Oncology.

[121] Ma C., Wang X., Guo J., Yang B., Li Y. (2023). Challenges
and future of HER2-positive gastric cancer therapy. Frontiers in Oncology.

[122] Li W., Zhang X., Du Y., Zhang Y., Lu J., Hu W., Zhao J. (2022). HER2-targeted
advanced metastatic gastric/gastroesophageal
junction adenocarcinoma: treatment landscape and future perspectives. Biomarker Research.

[123] Shayeb A. M., Kurzrock R., Adashek J. J., Kato S. (2023). Comprehensive
analysis of human epidermal growth factor receptor 2 through DNA,
mRNA, and protein in diverse malignancies. JCO
Precision Oncology.

[124] Swain S. M., Shastry M., Hamilton E. (2023). Targeting HER2-positive
breast cancer: Advances and future directions. Nat. Rev. Drug Discovery.

[125] Lambert J. M., Chari R. V. J. (2014). Ado-trastuzumab
emtansine (T-DM1):
An antibody–drug conjugate (ADC) for HER2-positive breast cancer. J. Med. Chem..

[126] Ado-trastuzumab emtansine. https://www.cancer.gov/publications/dictionaries/cancer-terms/def/ado-trastuzumab-emtansine (accessed Aug 20, 2024).

[127] Hubalek M., Brantner C., Marth C. (2012). Role of pertuzumab
in the treatment of HER2-positive breast cancer. Breast Cancer (Dove Med. Press).

[128] Baselga J., Gelmon K. A., Verma S., Wardley A., Conte P., Miles D., Bianchi G., Cortes J., McNally V. A., Ross G. A. (2010). Phase II trial of pertuzumab
and trastuzumab in patients with human epidermal growth factor receptor
2-positive metastatic breast cancer that progressed during prior trastuzumab
therapy. J. Clin Oncol.

[129] Tevaarwerk A. J., Kolesar J. M. (2009). Lapatinib: A small-molecule
inhibitor
of epidermal growth factor receptor and human epidermal growth factor
receptor-2 tyrosine kinases used in the treatment of breast cancer. Clin. Ther..

[130] Kim H. P., Yoon Y. K., Kim J. W., Han S. W., Hur H. S., Park J., Lee J. H., Oh D. Y., Im S. A., Bang Y. J., Kim T. Y. (2009). Lapatinib,
a dual
EGFR and HER2 tyrosine kinase inhibitor, downregulates thymidylate
synthase by inhibiting the nuclear translocation of EGFR and HER2. PLoS One.

[131] Lee J. B., Kim H. R., Ha S. J. (2022). Immune checkpoint
inhibitors in 10 years: Contribution of basic research and clinical
application in cancer immunotherapy. Immune
Network.

[132] Stark M. C., Joubert A. M., Visagie M. H. (2023). Molecular farming
of pembrolizumab and nivolumab. Int. J. Mol.
Sci..

[133] Salifu I., Singh N., Berraondo M., Remon J., Salifu S., Severson E., Quintana A., Peiró S., Ramkissoon S., Vidal L. (2023). Antibody-drug
conjugates, immune-checkpoint inhibitors, and their combination in
advanced non-small cell lung cancer. Cancer
Treatment and Research Communications.

[134] Wang K., Wei G., Liu D. (2012). CD19: A biomarker
for
B cell development, lymphoma diagnosis and therapy. Experimental Hematology & Oncology.

[135] Bailly S., Cartron G., Chaganti S., Córdoba R., Corradini P., Düll J., Ferrarini I., Osborne W., Rosenwald A., Sancho J. M. (2022). Targeting
CD19 in diffuse large B-cell lymphoma: An expert opinion paper. Hematol Oncol.

[136] Horna P., Nowakowski G., Endell J., Boxhammer R. (2019). Comparative
assessment of surface CD19 and CD20 expression on B-cell lymphomas
from clinical biopsies: Implications for targeted rherapies. Blood.

[137] Susa, K. J. ; Bradshaw, G. A. ; Eisert, R. J. ; Schilling, C. M. ; Kalocsay, M. ; Blacklow, S. C. ; Kruse, A. C. A spatiotemporal map of co-receptor signaling networks underlying B cell activation. bioRxiv 2023. DOI: 10.1101/2023.03.17.533227 PMC11256977 38850533

[138] Modulation of CD19 surface expression in B cell acute lymphoblastic leukemia. Nat. Immunol. 2022, 23 (10), 1410–1411. DOI: 10.1038/s41590-022-01316-w. 36151397 PMC9510525

[139] Elsallab M., Ellithi M., Hempel S., Abdel-Azim H., Abou-el-Enein M. (2023). Long-term response to autologous anti-CD19 chimeric
antigen receptor T cells in relapsed or refractory B cell acute lymphoblastic
leukemia: a systematic review and meta-analysis. Cancer Gene Ther..

[140] National Institutes of Health CAR T cells: Engineering patients’ immune cells to treat their cancers. https://www.cancer.gov/about-cancer/treatment/research/car-t-cells#:~:text=Need%20help%20finding%20cancer%20treatment,therapy%2C%20is%20more%20than%20$450%2C000. (accessed Aug 20, 2024).

[141] Awasthi R., Maier H. J., Zhang J., Lim S. (2023). Kymriah®
(tisagenlecleucel) - An overview of the clinical development journey
of the first approved CAR-T therapy. Hum Vaccin
Immunother.

[142] Leahy A. B., Elgarten C. W., Grupp S. A., Maude S. L., Teachey D. T. (2018). Tisagenlecleucel
for the treatment of B-cell acute
lymphoblastic leukemia. Expert Review of Anticancer
Therapy.

[143] FDA FDA approves axicabtagene ciloleucel for second-line treatment of large B-cell lymphoma. 2022. https://www.fda.gov/drugs/resources-information-approved-drugs/fda-approves-axicabtagene-ciloleucel-second-line-treatment-large-b-cell-lymphoma (accessed Aug 20, 2024).

[144] Sharma P., Kasamon Y. L., Lin X., Xu Z., Theoret M. R., Purohit-Sheth T. (2023). FDA approval summary: Axicabtagene
ciloleucel for second-line treatment of large B-cell lymphoma. Clin. Cancer Res..

[145] Bristol Myers Squibb U.S. Food and Drug Administration approves Bristol Myers Squibb’s breyanzi as a new CAR T cell therapy for relapsed or refractory mantle cell lymphoma. 2024. https://news.bms.com/news/corporate-financial/2024/U.S.-Food-and-Drug-Administration-Approves-Bristol-Myers-Squibbs-Breyanzi-as-a-New-CAR-T-Cell-Therapy-for-Relapsed-or-Refractory-Mantle-Cell-Lymphoma/default.aspx#:~:text=Breyanzi%20is%20approved%20in%20the,at%20least%20two%20prior%20lines (accessed Aug 20, 2024).

[146] In brief: Lisocabtagene maraleucel (Breyanzi) for large B-cell lymphoma. 2023. https://secure.medicalletter.org/TML-article-1679f (accessed Aug 20, 2024). 10.58347/tml.2023.1679f 37339093

[147] Zhu M., Wu B., Brandl C., Johnson J., Wolf A., Chow A., Doshi S. (2016). Blinatumomab,
a bispecific T-cell
engager (BiTE(®)) for CD-19 targeted cancer immunotherapy: Clinical
pharmacology and its implications. Clinical
Pharmacokinetics.

[148] Goebeler M. E., Bargou R. (2016). Blinatumomab: A CD19/CD3
bispecific
T cell engager (BiTE) with unique anti-tumor efficacy. Leukemia & Lymphoma.

[149] Calabretta E., Hamadani M., Zinzani P. L., Caimi P., Carlo-Stella C. (2022). The antibody-drug conjugate loncastuximab
tesirine
for the treatment of diffuse large B-cell lymphoma. Blood.

[150] St. Martin Y., Franz J. K., Agha M. E., Lazarus H. M. (2023). Failure
of CAR-T cell therapy in relapsed and refractory large cell lymphoma
and multiple myeloma: An urgent unmet need. Blood Rev..

[151] Fu Z., Li S., Han S., Shi C., Zhang Y. (2022). Antibody drug
conjugate: The “biological missile” for targeted cancer
therapy. Signal Transduction and Targeted Therapy.

[152] Sankar K., Ye J. C., Li Z., Zheng L., Song W., Hu-Lieskovan S. (2022). The role of
biomarkers in personalized
immunotherapy. Biomarker Research.

[153] Xu H., Jia Z., Liu F., Li J., Huang Y., Jiang Y., Pu P., Shang T., Tang P., Zhou Y. (2023). Biomarkers and experimental
models for cancer immunology
investigation. MedComm (2020).

[154] Passaro A., Al Bakir M., Hamilton E. G., Diehn M., André F., Roy-Chowdhuri S., Mountzios G., Wistuba I. I., Swanton C., Peters S. (2024). Cancer biomarkers:
Emerging trends and clinical implications for personalized treatment. Cell.

[155] Zhou Y., Tao L., Qiu J., Xu J., Yang X., Zhang Y., Tian X., Guan X., Cen X., Zhao Y. (2024). Tumor biomarkers for diagnosis, prognosis and targeted
therapy. Signal Transduction and Targeted Therapy.

[156] Li Y., Ma Y., Wu Z., Zeng F., Song B., Zhang Y., Li J., Lui S., Wu M. (2021). Tumor mutational
burden predicting the efficacy of immune checkpoint inhibitors in
colorectal cancer: A systematic review and meta-analysis. Front. Immunol..

[157] Wang J., Song J., Liu Z., Zhang T., Liu Y. (2022). High tumor mutation burden indicates better prognosis in colorectal
cancer patients with KRAS mutations. Front.
Oncol..

[158] Marques A., Cavaco P., Torre C., Sepodes B., Rocha J. (2024). Tumor mutational burden in colorectal
cancer: Implications for treatment. Critical
Reviews in Oncology/Hematology.

[159] Sholl L. M., Hirsch F. R., Hwang D., Botling J., Lopez-Rios F., Bubendorf L., Mino-Kenudson M., Roden A. C., Beasley M. B., Borczuk A. (2020). The promises
and challenges of tumor mutation burden as an immunotherapy biomarker:
A perspective from the international association for the study of
lung cancer pathology committee. Journal of
Thoracic Oncology.

[160] Zhang T., Kurban E., Wang Z. (2023). Neoantigens:
The novel
precision cancer immunotherapy. Biologics.

[161] Evaluating immune checkpoint inhibition biomarkers. https://www.jax.org/education-and-learning/clinical-and-continuing-education/clinical-topics/tumor-testing/pd-l1# (accessed Aug 15, 2024).

[162] Jung J., Heo Y. J., Park S. (2023). High tumor
mutational
burden predicts favorable response to anti-PD-(L)­1 therapy in patients
with solid tumor: a real-world pan-tumor analysis. J. Immunother. Cancer.

[163] Vilar E., Gruber S. B. (2010). Microsatellite instability in colorectal
cancer-the stable evidence. Nature Reviews Clinical
Oncology.

[164] Quintanilha J. C. F., Graf R. P., Fisher V. A., Oxnard G. R., Ellis H., Panarelli N., Lin D. I., Li G., Huang R. S. P., Ross J. S. (2023). Comparative effectiveness
of immune checkpoint inhibitors vs chemotherapy in patients with metastatic
colorectal cancer with measures of microsatellite instability, mismatch
repair, or tumor mutational burden. JAMA Network
Open.

[165] Roth M. T., Das S. (2021). Pembrolizumab in unresectable
or
metastatic MSI-high colorectal cancer: Safety and efficacy. Expert Review of Anticancer Therapy.

[166] Cao J., Yang X., Chen S., Wang J., Fan X., Fu S., Yang L. (2022). The predictive
efficacy of tumor mutation burden in
immunotherapy across multiple cancer types: A meta-analysis and bioinformatics
analysis. Transl Oncol.

[167] Schrock A. B., Ouyang C., Sandhu J., Sokol E., Jin D., Ross J. S., Miller V. A., Lim D., Amanam I., Chao J. (2019). Tumor mutational burden
is predictive of response to
immune checkpoint inhibitors in MSI-high metastatic colorectal cancer. Annals of Oncology.

[168] Fakih M., Sandhu J. S., Ouyang C., Sokol E., Ross J. S., Miller V. A., Lim D., Chao J., Catenacci D. V. T., Cho M. T. (2019). Tumor
mutational burden
(TMB) may be a promising predictive biomarker of response to PD-1/PD-L1
targeting in MSI-H colorectal cancer. J. Clin.
Oncol..

[169] Gavrielatou N., Doumas S., Economopoulou P., Foukas P. G., Psyrri A. (2020). Biomarkers for immunotherapy response
in head and neck cancer. Cancer Treatment Reviews.

[170] Rodrigo J. P., Sánchez-Canteli M., Otero-Rosales M., Martínez-Camblor P., Hermida-Prado F., García-Pedrero J. M. (2024). Tumor mutational burden predictability
in head and neck squamous cell carcinoma patients treated with immunotherapy:
Systematic review and meta-analysis. Journal
of Translational Medicine.

[171] Marcus L., Fashoyin-Aje L. A., Donoghue M., Yuan M., Rodriguez L., Gallagher P. S., Philip R., Ghosh S., Theoret M. R., Beaver J. A., Pazdur R., Lemery S. J. (2021). FDA approval
summary: pembrolizumab for the treatment of tumor mutational burden–high
solid tumors. Clin. Cancer Res..

[172] Wildsmith S., Li W., Wu S., Stewart R., Morsli N., Raja R., Zhang Q., Ye J., He P., Shetty J. (2023). Tumor mutational burden
as a predictor of survival
with durvalumab and/or tremelimumab treatment in recurrent or metastatic
head and neck squamous cell carcinoma. Clin.
Cancer Res..

[173] FDA approves pembrolizumab for first-line treatment of head and neck squamous cell carcinoma. 2019. https://www.fda.gov/drugs/resources-information-approved-drugs/fda-approves-pembrolizumab-first-line-treatment-head-and-neck-squamous-cell-carcinoma#:~:text=Pembrolizumab%20was%20approved%20for%20use,pembrolizumab%20as%20a%20single%20agent. (accessed Aug 15, 2024).

[174] Wang Z., Duan J., Cai S., Han M., Dong H., Zhao J., Zhu B., Wang S., Zhuo M., Sun J. (2019). Assessment of blood
tumor mutational burden as a potential biomarker for immunotherapy
in patients with non–small cell lung cancer with use of a next-generation
sequencing cancer gene panel. JAMA Oncology.

[175] Si H., Kuziora M., Quinn K. J., Helman E., Ye J., Liu F., Scheuring U., Peters S., Rizvi N. A., Brohawn P. Z. (2021). A blood-based assay for assessment of tumor mutational burden in
first-line metastatic NSCLC treatment: Results from the MYSTIC study. Clin. Cancer Res..

[176] Galvano A., Gristina V., Malapelle U., Pisapia P., Pepe F., Barraco N., Castiglia M., Perez A., Rolfo C., Troncone G. (2021). The prognostic
impact of tumor mutational burden (TMB) in the first-line management
of advanced non-oncogene addicted non-small-cell lung cancer (NSCLC):
A systematic review and meta-analysis of randomized controlled trials. ESMO Open.

[177] Ricciuti B., Wang X., Alessi J. V., Rizvi H., Mahadevan N. R., Li Y. Y., Polio A., Lindsay J., Umeton R., Sinha R. (2022). Association of high
tumor mutation burden in non–small cell lung cancers with increased
immune infiltration and improved clinical outcomes of PD-L1 blockade
across PD-L1 expression levels. JAMA Oncology.

[178] KEYTRUDA is approved for the treatment of adult and pediatric patients with unresectable or metastatic MSI-H or dMMR solid tumors, as determined by an FDA-approved test, that have progressed following prior treatment and who have no satisfactory alternative treatment options. 2023. https://www.merck.com/news/fda-converts-to-full-approval-indication-for-keytruda-pembrolizumab-for-certain-adult-and-pediatric-patients-with-advanced-microsatellite-instability-high-msi-h-or-mismatch-repair-deficient/#:~:text=KEYTRUDA%20is%20indicated%20for%20the%20treatment%20of%20adult%20and%20pediatric,have%20no%20satisfactory%20alternative%20treatment (accessed Aug 16, 2024).

[179] FDA approves pembrolizumab for adults and children with TMB-H solid tumors. 2020. https://www.fda.gov/drugs/drug-approvals-and-databases/fda-approves-pembrolizumab-adults-and-children-tmb-h-solid-tumors (accessed Aug 16, 2024).

[180] Subbiah V., Solit D. B., Chan T. A., Kurzrock R. (2020). The FDA approval
of pembrolizumab for adult and pediatric patients with tumor mutational
burden (TMB) ≥ 10: A decision centered on empowering patients
and their physicians. Annals of Oncology.

[181] Six-year outcomes from Phase 3 CheckMate −227 trial show durable, long-term survival with opdivo (nivolumab) plus yervoy (ipilimumab) in the first-line treatment of patients with metastatic non-small cell lung cancer. 2023. https://news.bms.com/news/corporate-financial/2023/Six-Year-Outcomes-from-Phase-3-CheckMate--227-Trial-Show-Durable-Long-Term-Survival-with-Opdivo-nivolumab-Plus-Yervoy-ipilimumab-in-the-First-Line-Treatment-of-Patients-with-Metastatic-Non-Small-Cell-Lung-Cancer/default.aspx (accessed Aug 16, 2024).

[182] Clinical trial results for advanced non-small cell lung cancer (NSCLC). https://www.opdivo.com/nsclc/clinical-trial-results/advanced-immunotherapy-combination-chemo (accessed Aug 16, 2024).

[183] Hellmann M. D., Ciuleanu T.-E., Pluzanski A., Lee J. S., Otterson G. A., Audigier-Valette C., Minenza E., Linardou H., Burgers S., Salman P. (2018). Nivolumab plus ipilimumab in lung cancer with a high tumor mutational
burden. New England Journal of Medicine.

[184] Hoffman-Censits J. H., Choi W., Lombardo K., Hahn N. M., McConkey D. J., McGuire B., Parimi V., Matoso A. (2020). Expression
of nectin-4 in bladder cancer with variant histology. J. Clin. Oncol..

[185] Hoffman-Censits J. H., Lombardo K. A., Parimi V., Kamanda S., Choi W., Hahn N. M., McConkey D. J., McGuire B. M., Bivalacqua T. J., Kates M., Matoso A. (2021). Expression of nectin-4
in bladder urothelial carcinoma, in morphologic variants, and nonurothelial
histotypes. Applied Immunohistochemistry &
Molecular Morphology.

[186] Rodler S., Eismann L., Schlenker B., Casuscelli J., Brinkmann I., Sendelhofert A., Waidelich R., Buchner A., Stief C., Schulz G. B., Ledderose S. (2022). Expression of nectin-4 in variant histologies of bladder
cancer and its prognostic value—Need for biomarker testing
in high-risk patients?. Cancers.

[187] Heath E. I., Rosenberg J. E. (2021). The biology
and rationale of targeting
nectin-4 in urothelial carcinoma. Nature Reviews
Urology.

[188] Chu C. E., Sjöström M., Egusa E. A., Gibb E. A., Badura M. L., Zhu J., Koshkin V. S., Stohr B. A., Meng M. V., Pruthi R. S. (2021). Heterogeneity
in NECTIN4 expression across molecular subtypes of urothelial cancer
mediates sensitivity to enfortumab vedotin. Clin. Cancer Res..

[189] Challita-Eid P. M., Satpayev D., Yang P., An Z., Morrison K., Shostak Y., Raitano A., Nadell R., Liu W., Lortie D. R., Capo L., Verlinsky A., Leavitt M., Malik F., Aviña H., Guevara C. I., Dinh N., Karki S., Anand B. S., Pereira D. S., Joseph I. B. J., Doñate F., Morrison K., Stover D. R. (2016). Enfortumab Vedotin Antibody–Drug
Conjugate Targeting Nectin-4 Is a Highly Potent Therapeutic Agent
in Multiple Preclinical Cancer Models. Cancer
Res..

[190] Nectin-4: New antibody-drug conjugate (ADC) target. 2023. https://www.biochempeg.com/article/353.html (accessed Aug 20, 2024).

[191] Halford Z., Anderson M. K., Clark M. D. (2021). Enfortumab
vedotin-ejfv:
A first-in-class anti-nectin-4 antibody-drug conjugate for the management
of urothelial carcinoma. Annals of Pharmacotherapy.

[192] Ahtiainen M., Wirta E.-V., Kuopio T., Seppälä T., Rantala J., Mecklin J.-P., Böhm J. (2019). Combined prognostic
value of CD274 (PD-L1)/PDCDI (PD-1) expression and immune cell infiltration
in colorectal cancer as per mismatch repair status. Modern Pathology.

[193] Li Y., He M., Zhou Y., Yang C., Wei S., Bian X., Christopher O., Xie L. (2019). The prognostic and
clinicopathological roles of PD-L1 expression in colorectal cancer:
A systematic review and meta-analysis. Front.
Pharmacol..

[194] Wyss J., Dislich B., Koelzer V. H., Galván J. A., Dawson H., Hädrich M., Inderbitzin D., Lugli A., Zlobec I., Berger M. D. (2019). Stromal
PD-1/PD-L1
expression predicts outcome in colon cancer patients. Clinical Colorectal Cancer.

[195] Kuai W., Xu X., Yan J., Zhao W., Li Y., Wang B., Yuan N., Li Z., Jia Y. (2020). Prognostic
impact of PD-1 and Tim-3 expression in tumor tissue in stage I-III
colorectal cancer. BioMed. Research International.

[196] Lee L. H., Cavalcanti M. S., Segal N. H., Hechtman J. F., Weiser M. R., Smith J. J., Garcia-Aguilar J., Sadot E., Ntiamoah P., Markowitz A. J. (2016). Patterns and prognostic relevance of PD-1 and PD-L1 expression in
colorectal carcinoma. Modern Pathology.

[197] Ntomi V., Foukas P., Papaconstantinou D., Antonopoulou I., Pikoulis A., Panagiotides I., Pikoulis E., Syrigos K. (2021). The clinical
significance of PD-L1
in colorectal cancer (Review). Oncol. Rep..

[198] Hou W., Yi C., Zhu H. (2022). Predictive
biomarkers of colon cancer
immunotherapy: Present and future. Front. Immunol..

[199] André T., Shiu K.-K., Kim T. W., Jensen B. V., Jensen L. H., Punt C., Smith D., Garcia-Carbonero R., Benavides M., Gibbs P. (2020). Pembrolizumab
in microsatellite-instability–high
advanced colorectal cancer. New England Journal
of Medicine.

[200] NCI Staff FDA approves nivolumab for some metastatic colorectal cancers. 2017. https://www.cancer.gov/news-events/cancer-currents-blog/2017/nivolumab-fda-colorectal (accessed Aug 20, 2024).

[201] Lenz H.-J., Van Cutsem E., Limon M. L., Wong K. Y. M., Hendlisz A., Aglietta M., García-Alfonso P., Neyns B., Luppi G., Cardin D. B. (2022). First-line
nivolumab plus low-dose ipilimumab for microsatellite instability-high/mismatch
repair-deficient metastatic colorectal cancer: The phase II CheckMate
142 study. J. Clin. Oncol..

[202] Nivolumab (Opdivo). https://www.cancerresearchuk.org/about-cancer/treatment/drugs/nivolumab (accessed Aug 20, 2024).

[203] Kooshkaki O., Derakhshani A., Hosseinkhani N., Torabi M., Safaei S., Brunetti O., Racanelli V., Silvestris N., Baradaran B. (2020). Combination
of ipilimumab and nivolumab
in cancers: From clinical practice to ongoing clinical trials. Int. J. Mol. Sci..

[204] Dong Y., Sun Q., Zhang X. (2017). PD-1 and its ligands
are important immune checkpoints in cancer. Oncotarget.

[205] Xiao Y., Li Z. Z., Zhong N. N., Cao L. M., Liu B., Bu L. L. (2023). Charting new frontiers: Co-inhibitory immune checkpoint
proteins in therapeutics, biomarkers, and drug delivery systems in
cancer care. Translational Oncology.

[206] Mocan T., Sparchez Z., Craciun R., Bora C. N., Leucuta D. C. (2019). Programmed cell death protein-1 (PD-1)/programmed
death-ligand-1
(PD-L1) axis in hepatocellular carcinoma: Prognostic and therapeutic
perspectives. Clinical and Translational Oncology.

[207] Guo M., Yuan F., Qi F., Sun J., Rao Q., Zhao Z., Huang P., Fang T., Yang B., Xia J. (2020). Expression and clinical significance of LAG-3, FGL1, PD-L1 and CD8+T
cells in hepatocellular carcinoma using multiplex quantitative analysis. Journal of Translational Medicine.

[208] Li X.-S., Li J.-W., Li H., Jiang T. (2020). Prognostic
value of programmed cell death ligand 1 (PD-L1) for hepatocellular
carcinoma: a meta-analysis. Biosci. Rep..

[209] Sideras K., de Man R. A., Harrington S. M., Polak W. G., Zhou G., Schutz H. M., Pedroza-Gonzalez A., Biermann K., Mancham S., Hansen B. E. (2019). Circulating
levels of PD-L1 and Galectin-9 are associated with patient survival
in surgically treated hepatocellular carcinoma independent of their
intra-tumoral expression levels. Sci. Rep..

[210] Krafft U., Olah C., Reis H., Kesch C., Darr C., Grünwald V., Tschirdewahn S., Hadaschik B., Horvath O., Kenessey I. (2021). High serum
PD-L1 levels are associated with poor survival in urothelial cancer
patients treated with chemotherapy and immune checkpoint inhibitor
therapy. Cancers.

[211] Rajan A., Kim C., Heery C. R., Guha U., Gulley J. L. (2016). Nivolumab, anti-programmed death-1
(PD-1) monoclonal
antibody immunotherapy: Role in advanced cancers. Human Vaccines & Immunotherapeutics.

[212] Twomey J. D., Zhang B. (2021). Cancer immunotherapy
update: FDA-approved
checkpoint inhibitors and companion diagnostics. AAPS Journal.

[213] Merck’s KEYTRUDA® (pembrolizumab) significantly improved overall survival (OS) versus placebo in certain patients with advanced hepatocellular carcinoma (HCC) previously treated with sorafenib. 2022. https://www.merck.com/news/mercks-keytruda-pembrolizumab-significantly-improved-overall-survival-os-versus-placebo-in-certain-patients-with-advanced-hepatocellular-carcinoma-hcc-previously-treated-with-sora/ (accessed Aug 20, 2024).

[214] Psilopatis I., Damaskos C., Garmpi A., Sarantis P., Koustas E., Antoniou E. A., Dimitroulis D., Kouraklis G., Karamouzis M. V., Vrettou K. (2023). FDA-approved
monoclonal antibodies for unresectable hepatocellular carcinoma: What
do we know so far?. Int. J. Mol. Sci..

[215] TECENTRIQ® (atezolizumab) + Avastin® (bevacizumab) offers a same-day dosing schedule. https://www.tecentriq-hcp.com/hcc/dosing-and-administration/dosing.html (accessed Aug 20, 2024).

[216] El-Khoueiry A. (2020). Atezolizumab and bevacizumab
combination therapy for
hepatocellular carcinoma. Gastroenterol. Hepatol..

[217] Finn R. S., Qin S., Ikeda M., Galle P. R., Ducreux M., Kim T.-Y., Kudo M., Breder V., Merle P., Kaseb A. O. (2020). Atezolizumab
plus bevacizumab
in unresectable hepatocellular carcinoma. New
England Journal of Medicine.

[218] Yu J., Li M., Ren B., Cheng L., Wang X., Ma Z., Yong W. P., Chen X., Wang L., Goh B. C. (2023). Unleashing
the efficacy of immune checkpoint inhibitors for advanced hepatocellular
carcinoma: factors, strategies, and ongoing trials. Frontiers in Pharmacology.

[219] Sharma P., Goswami S., Raychaudhuri D., Siddiqui B. A., Singh P., Nagarajan A., Liu J., Subudhi S. K., Poon C., Gant K. L. (2023). Immune
checkpoint therapy—Current perspectives and future directions. Cell.

[220] Plage H., Samtleben H., Hofbauer S., Kornienko K., Weinberger S., Bruch P. G., Elezkurtaj S., Roßner F., Schallenberg S., Kluth M. (2022). GATA3
expression loss is linked to stage progression but is unrelated to
prognosis in muscle-invasive urothelial carcinoma of the bladder. Human Pathology.

[221] Zhang Q., Qi T., Long Y., Li X., Yao Y., Wu Q., Zou A., Qthmane B., Liu P. (2022). GATA3 predicts
the tumor microenvironment phenotypes and molecular subtypes for bladder
carcinoma. Front. Surg..

[222] Rana C., Babu S., Agarwal H., Singhai A., Kumar M., Singh V., Sinha R. J., Shankhwar S. N. (2021). Diagnostic
relevance of GATA 3 expression in urinary bladder carcinoma of divergent
differentiation and other histological variants. Indian Journal of Surgical Oncology.

[223] Naik M., Rao B. V., Fonseca D., Murthy S. S., Giridhar A., Sharma R., Raju K., Rao T. S., Challa S. (2021). GATA-3 expression in all grades and
different variants
of primary and metastatic urothelial carcinoma. Indian J. Surg. Oncol..

[224] Jackson C. L., Chen L., Hardy C. S., Ren K. Y., Visram K., Bratti V. F., Johnstone J., Sjödahl G., Siemens D. R., Gooding R. J., Berman D. M. (2022). Diagnostic
and prognostic implications of a three-antibody molecular subtyping
algorithm for non-muscle invasive bladder cancer. Journal of Pathology: Clinical Research.

[225] Yoo D., Min K. W., Pyo J. S., Kim N. Y. (2023). Diagnostic and prognostic
roles of GATA3 immunohistochemistry in urothelial carcinoma. Medicina.

[226] Wang C., Yang S., Jin L., Dai G., Yao Q., Xiang H., Zhang Y., Liu X., Xue B. (2020). Biological
and clinical significance of GATA3 detected from TCGA database and
FFPE sample in bladder cancer patients. OncoTargets
and Therapy.

[227] Ye F., Wang L., Castillo-Martin M., McBride R., Galsky M. D., Zhu J., Boffetta P., Zhang D. Y., Cordon-Cardo C. (2014). Biomarkers
for bladder cancer management: Present and future. Am. J. Clin. Exp. Urol..

[228] Eso Y., Shimizu T., Takeda H., Takai A., Marusawa H. (2020). Microsatellite
instability and immune checkpoint inhibitors: toward precision medicine
against gastrointestinal and hepatobiliary cancers. Journal of Gastroenterology.

[229] Bonneville R., Krook M. A., Kautto E. A., Miya J., Wing M. R., Chen H.-Z., Reeser J. W., Yu L., Roychowdhury S. (2017). Landscape of microsatellite instability across 39 cancer
types. JCO Precis. Oncol..

[230] Frankiw L., Baltimore D., Li G. (2019). Alternative mRNA splicing
in cancer immunotherapy. Nature Reviews Immunology.

[231] Park J., Chung Y.-J. (2019). Identification of neoantigens derived
from alternative splicing and RNA modification. Genomics Inf..

[232] Greco L., Rubbino F., Dal Buono A., Laghi L. (2023). Microsatellite instability
and immune response: From microenvironment
features to therapeutic actionability-lessons from colorectal cancer. Genes.

[233] Vos E. L., Maron S. B., Krell R. W., Nakauchi M., Fiasconaro M., Capanu M., Walch H. S., Chatila W. K., Schultz N., Ilson D. H. (2023). Survival of locally
advanced MSI-high gastric cancer patients treated with perioperative
chemotherapy: A retrospective cohort study. Annals of Surgery.

[234] Kang B. W., Chau I. (2020). Current status and future potential
of predictive biomarkers for immune checkpoint inhibitors in gastric
cancer. ESMO Open.

[235] Ozer M., Vegivinti C. T. R., Syed M., Ferrell M. E., Gonzalez Gomez C., Cheng S., Holder-Murray J., Bruno T., Saeed A., Sahin I. H. (2023). Neoadjuvant immunotherapy
for patients with dMMR/MSI-high gastrointestinal cancers: A changing
paradigm. Cancers.

[236] Nádorvári M. L., Lotz G., Kulka J., Kiss A. (2024). Tímár, J. Microsatellite
instability and mismatch repair
protein deficiency: Equal predictive markers?. Pathol. Oncol. Res..

[237] Li K., Luo H., Huang L., Luo H., Zhu X. (2020). Microsatellite
instability: A review of what the oncologist should know. Cancer Cell International.

[238] Zito Marino F., Amato M., Ronchi A., Panarese I., Ferraraccio F., De Vita F., Tirino G., Martinelli E., Troiani T., Facchini G. (2022). Microsatellite status
detection in gastrointestinal cancers: PCR/NGS is mandatory in negative/patchy
MMR immunohistochemistry. Cancers.

[239] Rosa, K. FDA grants full approval to pembrolizumab for select patients with MSI-H or dMMR solid tumors. 2023. https://www.onclive.com/view/fda-grants-full-approval-to-pembrolizumab-for-select-patients-with-msi-h-or-dmmr-solid-tumors (accessed Aug 20, 2024).

[240] He P., Ma L., Xu B., Wang Y., Li X., Chen H., Li Y. (2024). Research progress
and future directions
of immune checkpoint inhibitor combination therapy in advanced gastric
cancer. Ther. Adv. Med. Oncol..

[241] Li Y. N., Xie B., Zhang Y., He M. H., Xing Y., Mu D. M., Wang H., Guo R. (2023). Advances and
key focus areas in gastric cancer immunotherapy: A comprehensive scientometric
and clinical trial review (1999–2023). World Journal of Gastroenterology.

[242] Baxter M. A., Middleton F., Cagney H. P., Petty R. D. (2021). Resistance
to immune checkpoint inhibitors in advanced gastro-oesophageal cancers. Br. J. Cancer.

[243] Liu Y., Hu P., Xu L., Zhang X., Li Z., Li Y., Qiu H. (2023). Current progress
on predictive biomarkers for response
to immune checkpoint inhibitors in gastric cancer: How to maximize
the immunotherapeutic benefit?. Cancers.

[244] Anwanwan D., Singh S. K., Singh S., Saikam V., Singh R. (2020). Challenges in liver cancer and possible
treatment approaches. Biochim Biophys Acta Rev.
Cancer.

[245] Luo Y. Z., Zhu H. (2023). Immunotherapy for advanced or recurrent
hepatocellular carcinoma. World Journal of Gastrointestinal
Oncology.

[246] Chen Y., Hu H., Yuan X., Fan X., Zhang C. (2022). Advances in immune checkpoint inhibitors for advanced hepatocellular
carcinoma. Front. Immunol..

[247] Wojtukiewicz M. Z., Rek M. M., Karpowicz K., Górska M., Polityńska B., Wojtukiewicz A. M., Moniuszko M., Radziwon P., Tucker S. C., Honn K. V. (2021). Inhibitors
of immune checkpoints-PD-1, PD-L1, CTLA-4-new opportunities for cancer
patients and a new challenge for internists and general practitioners. Cancer and Metastasis Reviews.

[248] Qin S., Xu L., Yi M., Yu S., Wu K., Luo S. (2019). Novel immune checkpoint targets:
Moving beyond PD-1 and CTLA-4. Molecular Cancer.

[249] Whiteside T. L. (2022). Tumor-infiltrating lymphocytes and
their role in solid
tumor progression. Experientia Supplementum.

[250] McCarthy P. M., Valdera F. A., Smolinsky T. R., Adams A. M., O’Shea A. E., Thomas K. K., Van Decar S., Carpenter E. L., Tiwari A., Myers J. W. (2023). Tumor
infiltrating lymphocytes as an endpoint in cancer vaccine trials. Frontiers in Immunology.

[251] Gataa I., Mezquita L., Rossoni C., Auclin E., Kossai M., Aboubakar F., Le Moulec S., Massé J., Masson M., Radosevic-Robin N. (2021). Tumour-infiltrating
lymphocyte density is associated with favourable
outcome in patients with advanced non–small cell lung cancer
treated with immunotherapy. Eur. J. Cancer.

[252] Ruffini E., Asioli S., Filosso P. L., Lyberis P., Bruna M. C., Macrì L., Daniele L., Oliaro A. (2009). Clinical significance
of tumor-infiltrating lymphocytes in lung neoplasms. Annals of Thoracic Surgery.

[253] Corredor G., Wang X., Zhou Y., Lu C., Fu P., Syrigos K., Rimm D. L., Yang M., Romero E., Schalper K. A. (2019). Spatial architecture
and arrangement of tumor-infiltrating
lymphocytes for predicting likelihood of recurrence in early-stage
non-small cell lung cancer. Clin. Cancer Res..

[254] Wang L., Yang Z., Guo F., Chen Y., Wei J., Dai X., Zhang X. (2023). Research progress of biomarkers in
the prediction of anti-PD-1/PD-L1 immunotherapeutic efficiency in
lung cancer. Frontiers in Immunology.

[255] What are tumor-infiltrating lymphocytes (TILs)? https://www.dana-farber.org/cancer-care/treatment/cellular-therapies/til-therapy#:~:text=New%20Patient%20Appointments&text=Tumor%2Dinfiltrating%20lymphocyte%20(TIL)%20therapy%20is%20a%20type%20of,for%20other%20types%20of%20cancer. (accessed Aug 20, 2024).

[256] Donia, M. TIL therapy – An opportunity for a few centres? 2023. https://dailyreporter.esmo.org/esmo-congress-2023/cellular-therapies/til-therapy-an-opportunity-for-a-few-centres#:~:text=TIL%20therapy%20involves%20extracting%20TILs,challenges%20(Expert%20Opin%20Biol%20Ther. (accessed Aug 20, 2024).

[257] Zhao Y., Deng J., Rao S., Guo S., Shen J., Du F., Wu X., Chen Y., Li M., Chen M. (2022). Tumor infiltrating lymphocyte (TIL) therapy
for solid tumor treatment: Progressions and challenges. Cancers.

[258] Kirtane K., Elmariah H., Chung C. H., Abate-Daga D. (2021). Adoptive cellular
therapy in solid tumor malignancies: review of the literature and
challenges ahead. J. Immunother. Cancer.

[259] Li H., van der Merwe P. A., Sivakumar S. (2022). Biomarkers
of response to PD-1 pathway blockade. Br. J.
Cancer.

[260] Liu Y. T., Sun Z. J. (2021). Turning cold tumors into hot tumors
by improving T-cell infiltration. Theranostics.

[261] Morrissey K. M., Yuraszeck T. M., Li C. C., Zhang Y., Kasichayanula S. (2016). Immunotherapy
and novel combinations in oncology: current
landscape, challenges, and opportunities. Clinical
and Translational Science.

[262] Yu S., Wang Y., He P., Shao B., Liu F., Xiang Z., Yang T., Zeng Y., He T., Ma J. (2022). Effective
combinations of immunotherapy and radiotherapy
for cancer treatment. Frontiers in Oncology.

[263] Jin Y., Zuo Y., Li G., Liu W., Pan Y., Fan T., Fu X., Yao X., Peng Y. (2024). Advances in spatial
transcriptomics and its applications in cancer research. Molecular Cancer.

[264] Liao P., Huang Q., Zhang J., Su Y., Xiao R., Luo S., Wu Z., Zhu L., Li J., Hu Q. (2023). How single-cell techniques help us look into lung cancer
heterogeneity and immunotherapy. Frontiers in
Immunology.

[265] Lim J., Park C., Kim M., Kim H., Kim J., Lee D.-S. (2024). Advances in single-cell omics and
multiomics for high-resolution
molecular profiling. Experimental & Molecular
Medicine.

[266] Uscanga-Palomeque A. C., Chávez-Escamilla A. K., Alvizo-Báez C. A., Saavedra-Alonso S., Terrazas-Armendáriz L. D., Tamez-Guerra R. S., Rodríguez-Padilla C., Alcocer-González J. M. (2023). CAR-T cell
therapy: From the shop to cancer therapy. Int.
J. Mol. Sci..

[267] Jogalekar M. P., Rajendran R. L., Khan F., Dmello C., Gangadaran P., Ahn B. C. (2022). CAR T-cell-based gene therapy for
cancers: new perspectives, challenges, and clinical developments. Frontiers in Immunology.

[268] Li D., Li X., Zhou W.-L., Huang Y., Liang X., Jiang L., Yang X., Sun J., Li Z., Han W.-D., Wang W. (2019). Genetically engineered T cells for
cancer immunotherapy. Signal Transduction and
Targeted Therapy.

[269] Shah N., Chari A., Scott E., Mezzi K., Usmani S. Z. (2020). B-cell maturation antigen (BCMA) in multiple myeloma:
Rationale for targeting and current therapeutic approaches. Leukemia.

[270] Tai Y. T., Anderson K. C. (2015). Targeting B-cell
maturation antigen
in multiple myeloma. Immunotherapy.

[271] Kleber M., Ntanasis-Stathopoulos I., Terpos E. (2021). BCMA in multiple
myeloma-A promising key to therapy. J. Clin.
Med..

[272] Yu B., Jiang T., Liu D. (2020). BCMA-targeted immunotherapy for multiple
myeloma. Journal of Hematology & Oncology.

[273] Mishra A. K., Gupta A., Dagar G., Das D., Chakraborty A., Haque S., Prasad C. P., Singh A., Bhat A. A., Macha M. A. (2023). CAR-T-cell therapy in
multiple myeloma: B-cell maturation antigen (BCMA) and beyond. Vaccines.

[274] Sheykhhasan M., Ahmadieh-Yazdi A., Vicidomini R., Poondla N., Tanzadehpanah H., Dirbaziyan A., Mahaki H., Manoochehri H., Kalhor N., Dama P. (2024). CAR T therapies
in multiple myeloma: Unleashing the future. Cancer Gene Ther..

[275] Zhang X., Zhang H., Lan H., Wu J., Xiao Y. (2023). CAR-T cell therapy in multiple myeloma: Current limitations and potential
strategies. Frontiers in Immunology.

[276] Anderson L. D. (2022). Idecabtagene
vicleucel (ide-cel)
CAR T-cell therapy for relapsed and refractory multiple myeloma. Future Oncol.

[277] ABECMA (idecabtagene vicleucel). https://www.myeloma.org/abecma-idecabtagene-vicleucel-ide-cel (accessed Aug 20, 2024).

[278] Chekol Abebe E., Yibeltal
Shiferaw M., Tadele Admasu F., Asmamaw Dejenie T. (2022). Ciltacabtagene
autoleucel: The second anti-BCMA CAR
T-cell therapeutic armamentarium of relapsed or refractory multiple
myeloma. Front Immunol.

[279] CARVYKTI® is an infusion of your own T cells, genetically modified to fight multiple myeloma. https://www.carvykti.com/?utm_source=google&utm_medium=cpc&utm_campaign=GO-USA-ENG-PS-Carvykti-N/A-BC-EX-RN-DTC_Brand&utm_content=Carvykti&utm_term=carvykti+ciltacabtagene+autoleucel&gclid=CjwKCAjwoJa2BhBPEiwA0l0ImF5rj8zEdvvMM3Re7T45N96r8ChsEbE6_L_TAsuIAJjZUIPGLSBsAxoC8NQQAvD_BwE&gclsrc=aw.ds (accessed Aug 20, 2024).

[280] Miller K., Hashmi H., Rajeeve S. (2024). Beyond BCMA:
The next
wave of CAR T cell therapy in multiple myeloma. Frontiers in Oncology.

[281] Liu Z., Lei W., Wang H., Liu X., Fu R. (2024). Challenges
and strategies associated with CAR-T cell therapy in blood malignancies. Experimental Hematology & Oncology.

[282] Vanhooren J., Dobbelaere R., Derpoorter C., Deneweth L., Van Camp L., Uyttebroeck A., De Moerloose B., Lammens T. (2023). CAR-T in the treatment of acute myeloid
leukemia: Barriers and how to overcome them. Hemasphere.

[283] Kenderian S. S., Ruella M., Shestova O., Klichinsky M., Aikawa V., Morrissette J. J., Scholler J., Song D., Porter D. L., Carroll M. (2015). CD33-specific chimeric
antigen receptor T cells exhibit potent preclinical activity against
human acute myeloid leukemia. Leukemia.

[284] Teppert K., Yonezawa Ogusuku I. E., Brandes C., Herbel V., Winter N., Werchau N., Khorkova S., Wöhle C., Jelveh N., Bisdorf K. (2024). CAR’TCR-T cells
co-expressing CD33-CAR and dNPM1-TCR as superior dual-targeting approach
for AML treatment. Mol. Ther.: Oncol..

[285] Marvin-Peek J., Savani B. N., Olalekan O. O., Dholaria B. (2022). Challenges
and advances in chimeric antigen receptor therapy for acute myeloid
leukemia. Cancers.

[286] Aldoss I., Clark M., Song J. Y., Pullarkat V. (2020). Targeting
the alpha subunit of IL-3 receptor (CD123) in patients with acute
leukemia. Hum Vaccin Immunother.

[287] Kiyoi H., Kawashima N., Ishikawa Y. (2020). FLT3 mutations in acute
myeloid leukemia: Therapeutic paradigm beyond inhibitor development. Cancer Science.

[288] Kennedy V. E., Smith C. C. (2020). FLT3 mutations in
acute myeloid leukemia:
Key concepts and emerging controversies. Frontiers
in Oncology.

[289] Wang J., Wang W., Chen H., Li W., Huang T., Zhang W., Ling W., Lai P., Wang Y., Geng S. (2021). C-Type lectin-like molecule-1
as a biomarker for diagnosis and prognosis in acute myeloid leukemia:
A preliminary study. BioMed. Research International.

[290] Darwish N. H. E., Sudha T., Godugu K., Elbaz O., Abdelghaffar H. A., Hassan E. E. A., Mousa S. A. (2016). Acute myeloid
leukemia
stem cell markers in prognosis and targeted therapy: Potential impact
of BMI-1, TIM-3 and CLL-1. Oncotarget.

[291] Boucher J. C., Shrestha B., Vishwasrao P., Leick M., Cervantes E. V., Ghafoor T., Reid K., Spitler K., Yu B., Betts B. C. (2023). Bispecific
CD33/CD123 targeted chimeric antigen receptor T cells for the treatment
of acute myeloid leukemia. Mol. Ther.: Oncol..

[292] Zhou Z, Tao C., Li J., Tang J. C.-O., Chan A. S.-C., Zhou Y. (2022). Chimeric antigen receptor
T cells applied to solid
tumors. Front. Immunol..

[293] Grosser R., Cherkassky L., Chintala N., Adusumilli P. S. (2019). Combination
immunotherapy with CAR T cells and checkpoint blockade for the treatment
of solid tumors. Cancer Cell.

[294] Al-Haideri M., Tondok S. B., Safa S. H., Maleki A. H., Rostami S., Jalil A. T., Al-Gazally M. E., Alsaikhan F., Rizaev J. A., Mohammad T. A. M., Tahmasebi S. (2022). CAR-T cell
combination therapy: The next revolution in cancer treatment. Cancer Cell Int..

[295] Luo J., Zhang X. (2024). Challenges and innovations in CAR-T
cell therapy: A
comprehensive analysis. Front. Oncol..

[296] Li W., Wu L., Huang C., Liu R., Li Z., Liu L., Shan B. (2020). Challenges and strategies
of clinical application of
CAR-T therapy in the treatment of tumors—a narrative review. Annals of Translational Medicine.

[297] Posey A. D., Young R. M., June C. H. (2024). Future
perspectives on engineered T cells for cancer. Trends in Cancer.

[298] Zhang Y., Qin D., Shou A. C., Liu Y., Wang Y., Zhou L. (2023). Exploring CAR-T cell therapy side
effects: Mechanisms and management strategies. J. Clin. Med..

[299] Zhang C., Wang Z., Yang Z., Wang M., Li S., Li Y., Zhang R., Xiong Z., Wei Z., Shen J. (2017). Phase I escalating-dose trial of CAR-T therapy targeting
CEA­(+) metastatic colorectal cancers. Molecular
Therapy.

[300] Dagar G., Gupta A., Masoodi T., Nisar S., Merhi M., Hashem S., Chauhan R., Dagar M., Mirza S., Bagga P. (2023). Harnessing the potential
of CAR-T cell therapy: Progress, challenges, and future directions
in hematological and solid tumor treatments. Journal of Translational Medicine.

[301] Djaballah S. A., Daniel F., Milani A., Ricagno G., Lonardi S. (2022). HER2 in colorectal cancer: The long and winding road
from negative predictive factor to positive actionable target. Am. Soc. Clin. Oncol. Educ. Book.

[302] Xu J., Meng Q., Sun H., Zhang X., Yun J., Li B., Wu S.-Y., Li X., Yang H., Zhu H. (2021). HER2-specific chimeric antigen receptor-T cells for targeted therapy
of metastatic colorectal cancer. Cell Death
& Disease.

[303] Qin X., Wu F., Chen C., Li Q. (2022). Recent advances in
CAR-T cells therapy for colorectal cancer. Frontiers
in Immunology.

[304] Li H., Huang Y., Jiang D.-Q., Cui L.-Z., He Z., Wang C., Zhang Z.-W., Zhu H.-L., Ding Y.-M., Li L.-F. (2018). Antitumor activity of EGFR-specific CAR T cells against
non-small-cell lung cancer cells in vitro and in mice. Cell Death & Disease.

[305] Janani B., Vijayakumar M., Priya K., Kim J. H., Prabakaran D. S., Shahid M., Al-Ghamdi S., Alsaidan M., Othman Bahakim N., Hassan Abdelzaher M., Ramesh T. (2022). EGFR-based targeted therapy for colorectal cancer-promises
and challenges. Vaccines.

[306] Staudt R. E., Carlson R. D., Snook A. E. (2022). Targeting
gastrointestinal
cancers with chimeric antigen receptor (CAR)-T cell therapy. Cancer Biology & Therapy.

[307] Lisby A. N., Flickinger J. C., Bashir B., Weindorfer M., Shelukar S., Crutcher M., Snook A. E., Waldman S. A. (2021). GUCY2C
as a biomarker to target precision therapies
for patients with colorectal cancer. Expert
Review on Precision Medicine and Drug Development.

[308] Entezari A. A., Snook A. E., Waldman S. A. (2021). Guanylyl
cyclase
2C (GUCY2C) in gastrointestinal cancers: Recent innovations and therapeutic
potential. Expert Opinion on Therapeutic Targets.

[309] Zhai X., Mao L., Wu M., Liu J., Yu S. (2023). Challenges of anti-mesothelin
CAR-T-cell therapy. Cancers.

[310] Zhang Q., Liu G., Liu J., Yang M., Fu J., Liu G., Li D., Gu Z., Zhang L., Pan Y. (2021). The antitumor capacity
of mesothelin-CAR-T cells in
targeting solid tumors in mice. Molecular Therapy
- Oncolytics.

[311] Malla M., Kumar Deshmukh S., Wu S., Samec T., Olevian D., Naili R., Bassel E. R., Xiu J., Farrell A., Lenz H.-J. (2023). Mesothelin expression
correlates with elevated inhibitory immune activity in patients with
colorectal cancer. Cancer Gene Ther..

[312] Hester R., Mazur P. K., McAllister F. (2021). Immunotherapy
in pancreatic adenocarcinoma: Beyond ″copy/paste″. Clin. Cancer Res..

[313] Ho W. J., Jaffee E. M., Zheng L. (2020). The tumour
microenvironment
in pancreatic cancer - clinical challenges and opportunities. Nature Reviews Clinical Oncology.

[314] Kumar A., Gautam V., Sandhu A., Rawat K., Sharma A., Saha L. (2023). Current and emerging
therapeutic
approaches for colorectal cancer: A comprehensive review. World Journal of Gastrointestinal Surgery.

[315] Heady, D. ASCO: Combination therapy significantly improves outcomes for patients with metastatic colorectal cancer. 2024. https://www.uclahealth.org/news/release/asco-combination-therapy-significantly-improves-outcomes (accessed Aug 20, 2024).

[316] Liu L., Huang X., Shi F., Song J., Guo C., Yang J., Liang T., Bai X. (2022). Combination therapy
for pancreatic cancer: Anti-PD-(L)­1-based strategy. Journal of Experimental & Clinical Cancer Research.

[317] Mucileanu A., Chira R., Mircea P. A. (2021). PD-1/PD-L1
expression
in pancreatic cancer and its implication in novel therapies. Medicine and Pharmacy Reports.

[318] Ye X., Yu Y., Zheng X., Ma H. (2024). Clinical immunotherapy
in pancreatic cancer. Cancer Immunology, Immunotherapy.

[319] Winstead, E. Pembrolizumab improves survival in advanced triple-negative breast cancer. 2022. https://www.cancer.gov/news-events/cancer-currents-blog/2022/pembrolizumab-triple-negative-breast-cancer-improves-survival#:~:text=After%20the%202020%20approval%20of,different%20trial%2C%20KEYNOTE%2D522. (accessed Aug 20, 2024).

[320] FDA FDA approves pembrolizumab combination for the first-line treatment of cervical cancer. 2021. https://www.fda.gov/drugs/resources-information-approved-drugs/fda-approves-pembrolizumab-combination-first-line-treatment-cervical-cancer#:~:text=Search,by%20an%20FDA%2Dapproved%20test. (accessed Aug 20, 2024).

[321] Consistent, durable OS across PD-L1 < 1% and PD-L1 ≥ 1% at 5 years with OPDIVO + YERVOY and 2 cycles of chemo vs chemo. https://www.opdivohcp.com/efficacy/nsclc/opdivo-yervoy-chemo?cid=sem_2245181&gclid=CjwKCAjwoJa2BhBPEiwA0l0ImIp97Rnagfdp75-Yt4XZWB5gpePBrqTcFanMtirMfTMXQN_UQgoGNhoCEbEQAvD_BwE&gclsrc=aw.ds (accessed Aug 20, 2024).

[322] Chen I. M., Johansen J. S., Theile S., Hjaltelin J. X., Novitski S. I., Brunak S., Hasselby J. P., Willemoe G. L., Lorentzen T., Madsen K. (2022). Randomized
phase II
study of nivolumab with or without ipilimumab combined with stereotactic
body radiotherapy for refractory metastatic pancreatic cancer (CheckPAC). Journal of Clinical Oncology.

[323] Stenger, M. Ipilimumab/Nivolumab in patients with metastatic pancreatic or biliary cancer and HRD pathogenic germline variants. 2022. https://ascopost.com/issues/june-10-2022/ipilimumabnivolumab-in-patients-with-metastatic-pancreatic-or-biliary-cancer-and-hrd-pathogenic-germline-variants/ (accessed Aug 20, 2024). 10.1001/jamaoncol.2022.0611 PMC9026238 35446342

[324] Miyabayashi K., Ijichi H., Fujishiro M. (2022). The role of
the microbiome in pancreatic cancer. Cancers.

[325] Pushalkar S., Hundeyin M., Daley D., Zambirinis C. P., Kurz E., Mishra A., Mohan N., Aykut B., Usyk M., Torres L. E. (2018). The
pancreatic cancer
microbiome promotes oncogenesis by induction of innate and adaptive
immune suppression. Cancer Discovery.

[326] Pourali G., Kazemi D., Chadeganipour A. S., Arastonejad M., Kashani S. N., Pourali R., Maftooh M., Akbarzade H., Fiuji H., Hassanian S. M. (2024). Microbiome as a biomarker
and therapeutic target in pancreatic cancer. BMC Microbiology.

[327] Dumitru A., Dobrica E. C., Croitoru A., Cretoiu S. M., Gaspar B. S. (2022). Focus on PD-1/PD-L1 as a therapeutic target in ovarian
cancer. Int. J. Mol. Sci..

[328] Song M., Chen X., Wang L., Zhang Y. (2018). Future of
anti-PD-1/PD-L1 applications: Combinations with other therapeutic
regimens. Chinese Journal of Cancer Research.

[329] Demircan N. C., Boussios S., Tasci T., Öztürk M. A. (2020). Current
and future immunotherapy approaches in ovarian cancer. Annals of Translational Medicine.

[330] Colombo I., Karakasis K., Suku S., Oza A. M. (2023). Chasing
immune checkpoint inhibitors in ovarian cancer: Novel combinations
and biomarker discovery. Cancers.

[331] Kooshkaki O., Derakhshani A., Safarpour H., Najafi S., Vahedi P., Brunetti O., Torabi M., Lotfinejad P., Paradiso A. V., Racanelli V. (2020). The latest findings of PD-1/PD-L1 inhibitor application in gynecologic
cancers. International Journal of Molecular
Sciences.

[332] TECVAYLI®, a bispecific antibody, is the first treatment of its kind designed to fight multiple myeloma. https://www.tecvayli.com/how-tecvayli-works?utm_source=google&utm_medium=cpc&utm_campaign=GO-USA-ENG-PS-Tecvayli-GP-PH-RN-DTC_BCMA&utm_content=BCMA&utm_term=bcma+myeloma&gclid=CjwKCAjwoJa2BhBPEiwA0l0ImLC_0k6TzXnxdC1vTuYq4UbFDOyIzmThJUltlhqjY8o5iIWkl2gF4xoCcd4QAvD_BwE&gclsrc=aw.ds (accessed Aug 20, 2024).

[333] Riccardi F., Tangredi C., Dal Bo M., Toffoli G. (2024). Targeted therapy
for multiple myeloma: An overview on CD138-based strategies. Frontiers in Oncology.

[334] Bruins W. S. C., Zweegman S., Mutis T., van de Donk N. W. C. J. (2020). Targeted
therapy with immunoconjugates for multiple myeloma. Front. Immunol..

[335] Hartley-Brown M., Richardson P. (2022). Antibody-drug conjugate therapies
in multiple myeloma-what’s next on the horizon?. Explor. Targeted Anti-Tumor Ther..

[336] Offidani M., Corvatta L., Morè S., Olivieri A. (2021). Belantamab mafodotin for the treatment of multiple
myeloma: An overview of the clinical efficacy and safety. Drug Design, Development and Therapy.

[337] Orlowski R. Z., Nagler A., Sonneveld P., Bladé J., Hajek R., Spencer A., San Miguel J., Robak T., Dmoszynska A., Horvath N. (2007). Randomized Phase III
study of pegylated liposomal doxorubicin plus bortezomib compared
with bortezomib alone in relapsed or refractory multiple myeloma:
Combination therapy improves time to. J. Clin.
Oncol..

[338] Paul S., Lal G. (2017). The molecular mechanism of natural
killer cells function and its importance in cancer immunotherapy. Front. Immunol..

[339] Cleveland Clinic Natural killer cells. https://my.clevelandclinic.org/health/body/24898-natural-killer-cells (accessed Aug 20, 2024).

[340] Liu S., Galat V., Galat Y., Lee Y. K. A., Wainwright D., Wu J. (2021). NK cell-based cancer
immunotherapy: From basic biology to clinical
development. J. Hematol. Oncol..

[341] Yu Y. (2023). The function of NK cells in tumor metastasis and NK
cell-based immunotherapy. Cancers.

[342] Eissmann, P. Natural killer cells https://www.immunology.org/public-information/bitesized-immunology/cells/natural-killer-cells#:~:text=Once%20the%20decision%20is%20made%20to%20kill%2C,leads%20to%20lysis%20of%20the%20target%20cell. (accessed Aug 20, 2024).

[343] Jiang H., Jiang J. (2023). Balancing act: The complex role of
NK cells in immune regulation. Front. Immunol..

[344] Liu Y., Lu X., Qin N., Qiao Y., Xing S., Liu W., Feng F., Liu Z., Sun H. (2021). STING, a promising
target for small molecular immune modulator: A review. Eur. J. Med. Chem..

[345] He L., Xiao X., Yang X., Zhang Z., Wu L., Liu Z. (2017). STING signaling in tumorigenesis and cancer therapy:
A friend or
foe?. Cancer Letters.

[346] Luo K., Li N., Ye W., Gao H., Luo X., Cheng B. (2022). Activation of stimulation of interferon
genes (STING) signal and
cancer immunotherapy. Molecules.

[347] Kuhl N., Linder A., Philipp N., Nixdorf D., Fischer H., Veth S., Kuut G., Xu T. T., Theurich S., Carell T. (2023). STING
agonism turns
human T cells into interferon-producing cells but impedes their functionality. EMBO Rep.

[348] Zhu Y., An X., Zhang X., Qiao Y., Zheng T., Li X. (2019). STING: A master regulator in the cancer-immunity cycle. Molecular Cancer.

[349] Richter F., Paget C., Apetoh L. (2023). STING-driven activation
of T cells: Relevance for the adoptive cell therapy of cancer. Cell Stress.

[350] Pan X., Zhang W., Guo H., Wang L., Wu H., Ding L., Yang B. (2023). Strategies involving STING pathway
activation for cancer immunotherapy: Mechanism and agonists. Biochem. Pharmacol..

[351] Huang C., Shao N., Huang Y., Chen J., Wang D., Hu G., Zhang H., Luo L., Xiao Z. (2023). Overcoming challenges in the delivery of STING agonists for cancer
immunotherapy: A comprehensive review of strategies and future perspectives. Materials Today Bio.

[352] Su T., Zhang Y., Valerie K., Wang X. Y., Lin S., Zhu G. (2019). STING activation in cancer immunotherapy. Theranostics.

[353] Wu Y.-T., Fang Y., Wei Q., Shi H., Tan H., Deng Y., Zeng Z., Qiu J., Chen C., Sun L., Chen Z. J. (2022). Tumor-targeted delivery
of a STING agonist improves
cancer immunotherapy. Proc. Natl. Acad. Sci.
U. S. A..

[354] Meza Guzman L. G., Keating N., Nicholson S. E. (2020). Natural
killer cells: Tumor surveillance and signaling. Cancers.

[355] Lerner E. C., Woroniecka K. I., D’Anniballe V.
M., Wilkinson D. S., Mohan A. A., Lorrey S. J., Waibl-Polania J., Wachsmuth L. P., Miggelbrink A. M., Jackson J. D. (2023). CD8­(+)
T cells maintain killing of MHC-I-negative tumor cells through the
NKG2D-NKG2DL axis. Nature Cancer.

[356] Zamora A. E., Crawford J. C., Thomas P. G. (2018). Hitting
the target:
How T cells detect and eliminate tumors. J.
Immunol..

[357] Waldman A. D., Fritz J. M., Lenardo M. J. (2020). A guide to cancer
immunotherapy: From T cell basic science to clinical practice. Nature Reviews Immunology.

[358] Habif G., Crinier A., André P., Vivier E., Narni-Mancinelli E. (2019). Targeting natural killer cells in
solid tumors. Cellular & Molecular Immunology.

[359] Toffoli E. C., Sheikhi A., Höppner Y. D., de Kok P., Yazdanpanah-Samani M., Spanholtz J., Verheul H. M. W., van der Vliet H. J., de Gruijl T. D. (2021). Natural
killer cells and anti-cancer therapies: Reciprocal effects on immune
function and therapeutic response. Cancers.

[360] Wu S.-Y., Fu T., Jiang Y.-Z., Shao Z.-M. (2020). Natural
killer cells in cancer biology and therapy. Molecular Cancer.

[361] Guo M., Zhang H., Zheng J., Liu Y. (2020). Glypican-3:
A new target
for diagnosis and treatment of hepatocellular carcinoma. Journal of Cancer.

[362] Kolluri A., Ho M. (2019). The role of glypican-3
in regulating
Wnt, YAP, and hedgehog in liver cancer. Front.
Oncol..

[363] Hayat, M. A. 1 - Liver Carcinoma. In Handbook of Immunohistochemistry and in Situ Hybridization of Human Carcinomas, Hayat, M. A. Ed.; Vol. 3; Academic Press, 2005; pp 131–151.

[364] Zheng X., Liu X., Lei Y., Wang G., Liu M. (2022). Glypican-3: A novel and promising
target for the treatment of hepatocellular
carcinoma. Front. Oncol..

[365] Liu J., Xiao Q., Xiao J., Niu C., Li Y., Zhang X., Zhou Z. i., Shu G., Yin G. (2022). Wnt/β-catenin
signalling: function, biological mechanisms, and therapeutic opportunities. Signal Transduction and Targeted Therapy.

[366] Fu Q., Zheng Y., Fang W., Zhao Q., Zhao P., Liu L., Zhai Y., Tong Z., Zhang H., Lin M. (2023). RUNX-3-expressing
CAR T cells targeting glypican-3 in patients with
heavily pretreated advanced hepatocellular carcinoma: a phase I trial. EClinicalMedicine.

[367] Xie C., Monge B. M. C., Mabry-Hrones D., Coffman K. L., Hicks S., Redd B., Wood B., Highfill S., Ho M., Greten T. F. (2023). A phase I study
of GPC3 targeted CAR-T cell therapy
in advanced GPC3-expressing hepatocellular carcinoma (HCC). J. Clin. Oncol..

[368] Li D., Qin J., Zhou T., Li Y., Cheng X., Chen Z., Chen J., Zheng W. V. (2023). Bispecific
GPC3/PD-1
CAR-T cells for the treatment of HCC. Int. J.
Oncol..

[369] GPC3 targeted CAR-T cell therapy in advanced GPC3 expressing hepatocellular carcinoma (HCC). https://www.cancer.gov/research/participate/clinical-trials-search/v?id=NCI-2021-09114&r=1 (accessed Aug 22, 2024).

[370] Shimizu Y., Suzuki T., Yoshikawa T., Endo I., Nakatsura T. (2019). Next-generation cancer immunotherapy
Targeting glypican-3. Front. Oncol..

[371] Gupta S. C., Hevia D., Patchva S., Park B., Koh W., Aggarwal B. B. (2012). Upsides and downsides
of reactive oxygen species for
cancer: The roles of reactive oxygen species in tumorigenesis, prevention,
and therapy. Antioxidants & Redox Signaling.

[372] Kumari S., Badana A. K., Murali Mohan G., Shailender G., Malla R. (2018). Reactive oxygen species: A key constituent
in cancer survival. Biomarker Insights.

[373] Singh R., Manna P. P. (2022). Reactive oxygen species in cancer
progression and its role in therapeutics. Exploration
of Medicine.

[374] Snezhkina A. V., Kudryavtseva A. V., Kardymon O. L., Savvateeva M. V., Melnikova N. V., Krasnov G. S., Dmitriev A. A. (2019). ROS generation and
antioxidant defense systems in normal and malignant cells. Oxid. Med. Cell. Longevity.

[375] Arfin S., Jha N. K., Jha S. K., Kesari K. K., Ruokolainen J., Roychoudhury S., Rathi B., Kumar D. (2021). Oxidative
stress in cancer cell metabolism. Antioxidants.

[376] Cai H., Meng Z., Yu F. (2024). The involvement
of ROS-regulated
programmed cell death in hepatocellular carcinoma. Critical Reviews in Oncology/Hematology.

[377] An X., Yu W., Liu J., Tang D., Yang L., Chen X. (2024). Oxidative cell death
in cancer: Mechanisms and therapeutic opportunities. Cell Death & Disease.

[378] Beniwal N., Verma A., Putta C. L., Rengan A. K. (2024). Recent
trends in bio-nanomaterials and non-invasive combinatorial approaches
of photothermal therapy against cancer. Nanotheranostics.

[379] Han H. S., Choi K. Y. (2021). Advances in nanomaterial-mediated
photothermal cancer therapies: Toward clinical applications. Biomedicines.

[380] Xu P., Liang F. (2020). Nanomaterial-based tumor photothermal
immunotherapy. Int. J. Nanomed..

[381] Huang X., Lu Y., Guo M., Du S., Han N. (2021). Recent strategies for nano-based PTT combined with
immunotherapy:
From a biomaterial point of view. Theranostics.

[382] Yu S. H., Yoon I., Kim Y.-J. (2024). Ex vivo photothermal
treatment-induced immunogenic cell death for anticancer vaccine development. International Immunopharmacology.

[383] Zeng W., Li Z., Chen H., Zeng X., Mei L. (2022). An optimal portfolio of photothermal
combined immunotherapy. Cell Reports Physical
Science.

[384] Noh I., Son Y., Jung W., Kim M., Kim D., Shin H., Kim Y.-C., Jon S. (2021). Targeting the tumor
microenvironment with amphiphilic near-infrared cyanine nanoparticles
for potentiated photothermal immunotherapy. Biomaterials.

[385] Chasara R. S., Ajayi T. O., Leshilo D. M., Poka M. S., Witika B. A. (2023). Exploring novel strategies to improve
anti-tumour efficiency:
The potential for targeting reactive oxygen species. Heliyon.

[386] He M., Wang M., Xu T., Zhang M., Dai H., Wang C., Ding D., Zhong Z. (2023). Reactive oxygen species-powered
cancer immunotherapy: Current status and challenges. J. Controlled Release.

[387] Clinical trials using enfortumab vedotin. https://www.cancer.gov/research/participate/clinical-trials/intervention/enfortumab-vedotin (accessed Aug 22, 2024).

[388] Klümper N., Tran N. K., Zschäbitz S., Hahn O., Büttner T., Roghmann F., Bolenz C., Zengerling F., Schwab C., Nagy D. (2024). Nectin4
amplification is frequent in solid tumors and predicts enfortumab
vedotin response in metastatic urothelial cancer. Journal of Clinical Oncology.

[389] Maiorano B. A., Catalano M., Maiello E., Roviello G. (2023). Enfortumab
vedotin in metastatic urothelial carcinoma: The solution EVentually?. Front. Oncol..

[390] Wong J. L., Rosenberg J. E. (2021). Targeting nectin-4 by antibody-drug
conjugates for the treatment of urothelial carcinoma. Expert Opinion on Biological Therapy.

[391] Enfortumab vedotin-ejfv. https://www.cancer.gov/publications/dictionaries/cancer-drug/def/enfortumab-vedotin (accessed Aug 22, 2024).

[392] Powles T., Rosenberg J. E., Sonpavde G. P., Loriot Y., Durán I., Lee J.-L., Matsubara N., Vulsteke C., Castellano D., Wu C. (2021). Enfortumab
vedotin in previously treated advanced urothelial carcinoma. New England Journal of Medicine.

[393] Yu P., Zhu C., You X., Gu W., Wang X., Wang Y., Bu R., Wang K. (2024). The combination
of
immune checkpoint inhibitors and antibody-drug conjugates in the treatment
of urogenital tumors: a review insights from phase 2 and 3 studies. Cell Death & Disease.

[394] Fuentes-Antrás J., Genta S., Vijenthira A., Siu L. L. (2023). Antibody-drug conjugates: In search of partners of
choice. Trends in Cancer.

[395] Shvartsur A., Bonavida B. (2015). Trop2 and its overexpression
in cancers:
Regulation and clinical/therapeutic implications. Genes Cancer.

[396] Sawanyawisuth K., Tantapotinan N., Kraiklang R., Puapairoj A., Wongkham C., Riggins G. J., Wongkham S. (2016). Suppression
of trophoblast cell surface antigen 2 enhances proliferation and migration
in liver fluke-associated cholangiocarcinoma. Annals of Hepatology.

[397] Lombardi P., Filetti M., Falcone R., Altamura V., Paroni Sterbini F., Bria E., Fabi A., Giannarelli D., Scambia G., Daniele G. (2023). Overview of Trop-2
in cancer: From
pre-clinical studies to future directions in clinical settings. Cancers.

[398] Goldenberg D. M., Stein R., Sharkey R. M. (2018). The emergence of
trophoblast cell-surface antigen 2 (TROP-2) as a novel cancer target. Oncotarget.

[399] Shen M., Liu S., Stoyanova T. (2021). The role of
Trop2 in prostate cancer: An oncogene, biomarker, and therapeutic
target. Am. J. Clin. Exp. Urol..

[400] Tang G., Tang Q., Jia L., Chen Y., Lin L., Kuai X., Gong A., Feng Z. (2019). TROP2 increases growth
and metastasis of human oral squamous cell carcinoma through activation
of the PI3K/Akt signaling pathway. Int. J. Mol.
Med..

[401] Zhou D.-D., Zhai X.-T., Zhang L.-W., Xie Z.-H., Wang Y., Zhen Y.-S., Gao R.-J., Miao Q.-F. (2024). A new TROP2-targeting
antibody-drug conjugate shows potent antitumor efficacy in breast
and lung cancers. npj Precision Oncology.

[402] Fenn K. M., Kalinsky K. (2019). Sacituzumab govitecan:
Antibody-drug
conjugate in triple-negative breast cancer and other solid tumors. Drugs Today (Barc).

[403] Perrone E., Manara P., Lopez S., Bellone S., Bonazzoli E., Manzano A., Zammataro L., Bianchi A., Zeybek B., Buza N. (2020). Sacituzumab
govitecan, an antibody-drug conjugate targeting trophoblast cell-surface
antigen 2, shows cytotoxic activity against poorly differentiated
endometrial adenocarcinomas in vitro and in vivo. Molecular Oncology.

[404] Shastry M., Jacob S., Rugo H. S., Hamilton E. (2022). Antibody-drug
conjugates targeting TROP-2: Clinical development in metastatic breast
cancer. Breast.

[405] Goldenberg D. M., Sharkey R. M. (2019). Antibody-drug conjugates
targeting
TROP-2 and incorporating SN-38: A case study of anti-TROP-2 sacituzumab
govitecan. mAbs.

[406] Pavone G., Motta L., Martorana F., Motta G., Vigneri P. (2021). A new kid on the block: Sacituzumab
govitecan for the treatment of breast cancer and other solid tumors. Molecules.

[407] Tagawa S. T., Balar A. V., Petrylak D. P., Kalebasty A. R., Loriot Y., Fléchon A., Jain R. K., Agarwal N., Bupathi M., Barthelemy P. (2021). TROPHY-U-01: A Phase
II open-label study of sacituzumab govitecan in patients with metastatic
urothelial carcinoma progressing after platinum-based chemotherapy
and checkpoint inhibitors. Journal of Clinical
Oncology.

[408] Bardia A., Mayer I. A., Vahdat L. T., Tolaney S. M., Isakoff S. J., Diamond J. R., O’Shaughnessy J., Moroose R. L., Santin A. D., Abramson V. G. (2019). Sacituzumab
govitecan-hziy in refractory metastatic triple-negative breast cancer. New England Journal of Medicine.

[409] The Nobel Prize in Physiology or Medicine 2018. 2018. https://www.nobelprize.org/prizes/medicine/2018/summary/ (accessed Dec 20, 2024).

[410] Kang, C. ; Choi, J. Impact of co-occurrence on factual knowledge of large language models. In Findings of the Association for Computational Linguistics: EMNLP 2023, pp 7721–7735. Association for Computational Linguistics: Singapore, 2023 DOI: 10.18653/v1/2023.findings-emnlp.518.

[411] Pennington, J. ; Socher, R. ; Manning, C. GloVe: Global vectors for word representation. Association for Computational Linguistics, October, 2014; Doha, Qatar: pp 1532–1543. DOI: 10.3115/v1/D14-1162.

[412] Lin W., Wu X., Wang Z., Wan X., Li H. (2022). Topic network
analysis based on co-occurrence time series clustering. Mathematics.

[413] Petit, Q. ; Li, C. ; Emad, N. An efficient and scalable approach to build co-occurrence matrix for DNN’s embedding layer. In ICS ‘24: Proceedings of the 38th ACM International Conference on Supercomputing Association for Computing Machinery 2024, 286–297. DOI: 10.1145/3650200.3656629.

[414] Saravanan, K. ; Choudhury, M. ; Udupa, R. ; Kumaran, A. An empirical study of the occurrence and co-occurrence of named entities in natural language corpora, May, 2012; European Language Resources Association (ELRA): Istanbul, Turkey, pp 3118–3125.

[415] Leydesdorff L., Vaughan L. (2006). Co-occurrence matrices and their
applications in information science: Extending ACA to the Web environment. Journal of the American Society for Information Science and
Technology.

[416] White H. D., McCain K. W. (1998). Visualizing a discipline: An author
co-citation analysis of information science, 1972–1995. J. Am. Soc. Inf. Sci..

[417] Bouma, G. Normalized (pointwise) mutual information in collocation extraction. Proceedings of the Biennial GSCL Conference 2009 2009.

[418] Rijcken, E. ; Zervanou, K. ; Spruit, M. ; Scheepers, F. ; Kaymak, U. Effect of calculating pointwise mutual information using a fuzzy sliding window in topic modeling. In IEEE International Conference on Fuzzy Systems (FUZZ), IEEE 2023; pp 1–6. DOI: 10.1109/FUZZ52849.2023.10309675.

[419] Mikolov, T. ; Sutskever, I. ; Chen, K. ; Corrado, G. ; Dean, J. Distributed representations of words and phrases and their compositionality. In NIPS’13: Proceedings of the 27th International Conference on Neural Information Processing Systems, 2013; Curran Associates Inc.: Vol. 2, pp 3111 - 3119.

