# Supplementary material for: Emerging Concepts in Immuno-Oncology: Insights from Natural Language Processing-Driven Co-Occurrence Analysis
Source: ACS Omega. 2025 Jun 27;10(27):28587–614. doi: 10.1021/acsomega.5c00693 (PMC12268748; doi:10.1021/acsomega.5c00693)
Supplement: Supplementary file 1 [file ao5c00693_si_001.pdf]

Supplementary Information for

Emerging concepts in immuno-oncology: Insights from natural language processing (NLP)-driven co-occurrence analysis

Kavita A Iyer†, Rumiana Tenchov†, Julian M Ivanov, and Qiongqiong Angela Zhou*

CAS, A Division of the American Chemical Society, Columbus, Ohio 43210, United States

†Authors contributed equally

*Corresponding author, email: qzhou@cas.org

- Co-occurrence analysis methodology
- Traditional statistical approaches
- Co-occurrence analysis
- Figures S1-S7
- References

Co-occurrence analysis methodology

Co-occurrence analysis in bibliometrics, aims on investigating counts of co-occurring terms within a collection of textual units. It is used to study the relationships between bibliometric items that appear in the same unit being that a single sentence or a document. Co-occurrence measures, on the other hand, provide the useful data for mapping and understanding the structures in the underlying document collections.

In our study for the co-occurrence analysis, in general, we consider two distinct cases: 1) Terms co-occurring in a single sentence ("InSentence"), and 2) Terms co-occurring in an abstract ("InAbstract"). For the purposes of the current investigation, we introduce the following co-occurrence metrics:

sScore – InSentence co-occurrence Score

sScore = $\sum_{i}^{N=number\ of\ sentences\ in\ common\ documents} count\ of\ (T1\&T2)\ \ in\ a\ Sentence$

sScoreAverage = sScore / N_{total}

aScore – InAbstract co-occurrence Score

aScore = $\sum_{i}^{N=number\ of\ common\ documents} count\ of\ (T1\&T2)\ \ in\ an\ Abstract$

aScoreAverage = aScore / ND_{total}

Dist = Starting position of T2 - Starting position of T1

SumTotalDistances (in a single abstract) = $\sum_{i}^{All\ possible\ pairs\ of\ T1\&T2\ in\ an\ abstract} Dist(i)$

averageDistance = SumTotalDistances / ND_{total}

MinDistance – Minimum distance between T1&T2 in a single abstract

SumMinDistances = $\sum_{i}^{All\ abstracts} MinDistance(i)$

averageMinDistance = SumMinDistances / ND_{total}

where:

T1 – Term #1

T2 – Term #2

N_{total} - The total number of sentences in all common publications

ND_{total} - The number of common publications

The data domain of the study is defined by a search constructed by CAS subject matter experts and consists of various type of documents including journal articles, patents, conference proceedings, dissertations, and preprints published within certain predetermined time frame. For each document in the dataset, we obtain: the year of publication, type of document, CAS section, CAS subsection, up to date citations, abstract, and title. For identifying candidate phrases, we consider all n-grams containing 1 to 6 words from the abstract and titles of the documents after

performing Natural Language ToolKit (NLTK) procedures lemmatization and removal of English stop words. After the NLTK procedures and removing the phrases that do not contain enough information to be considered as scientific concepts we apply the algorithm for emerging topics identification.[1, 2] Once all emerging topics are identified we compute the co-occurrence measures, outlined above, between all possible pairs in the list of scientific topics. In addition, we also consider every single pair of co-occurring topics as a new scientific topic and evaluate it for emergence by computing all criteria for emerging topics[1, 2] for both co-occurrence general cases, "InSentence" and "InAbstract".

Traditional statistical approaches

The primary statistical methods for text analysis represent some of the earliest and most enduring techniques in computational text processing. These methods rely on mathematical and statistical principles to extract patterns and insights from text data without requiring the complex linguistic modeling of NLP or the deep learning architectures of modern machine learning approaches.

Term Frequency-Inverse Document Frequency (**TF-IDF**)[3-7] remains one of the most enduring statistical measures for text analysis. By weighing terms based on their frequency in documents and rarity across the corpus, it effectively identifies discriminative terms. While conceptually straightforward, TF-IDF provides remarkable performance in document classification and information retrieval tasks, though it disregards term semantics and word order.

N-gram statistical models capture local word dependencies by analyzing sequences of n words. These models excel at language modeling tasks and have proven valuable for spelling correction, word prediction, and machine translation. However, they struggle with long-range dependencies and require significant memory for larger values of n.

Latent Dirichlet Allocation (**LDA**)[8] applies Bayesian statistical principles to uncover latent topics within document collections. By modeling documents as mixtures of topics and topics as distributions over words, LDA provides an interpretable framework for content analysis. Its probabilistic foundation allows for uncertainty quantification, though topic coherence can vary, and parameter selection remains challenging.

The **Hierarchical Dirichlet Process** (**HDP**)[9] is a sophisticated nonparametric Bayesian model used in text analysis, particularly for topic modeling. It extends the popular Latent Dirichlet Allocation (LDA) by addressing one of LDA's key limitations: the need to pre-specify the number of topics.

Non-negative Matrix Factorization (**NMF**)[10] decomposes the term-document matrix into non-negative factors, revealing latent semantic structures. Compared to LDA, NMF often produces more coherent topics and scales better to larger datasets. However, it typically requires pre-determined topic numbers and can be sensitive to initialization.

Document Embeddings (**Doc2Vec**)[11, 12] extend word embedding concepts to entire documents. Doc2Vec provides fixed-length feature representations for variable-length texts. These embeddings facilitate document comparison and classification while preserving semantic information better than bag-of-words approaches.

Bayesian Text Analysis methods[13-15] provide a rigorous framework for incorporating prior knowledge and quantifying uncertainty in text analysis. Hierarchical Bayesian [28] models have proven particularly valuable for author attribution, stylometry, and sentiment analysis, though computational intensity can be prohibitive.

Exponential Family Embedding[16-18] models extend traditional embedding approaches using exponential family distributions, allowing for more flexible modeling of text data with different statistical properties, including count data and binary observations.

Statistical methods for text analysis continue to evolve, with hybrid approaches combining traditional statistical techniques with deep learning increasingly common. The choice of method depends significantly on the specific application, data characteristics, and interpretability requirements. Despite advances in neural approaches, traditional statistical methods remain valuable for their interpretability, lower computational requirements, and theoretical foundations, particularly when training data is limited.

Machine learning methods

The field of text data analysis has been revolutionized by machine learning approaches that can automatically discover patterns, extract insights, and make predictions from unstructured text data.

Supervised learning for text classification

Supervised learning algorithms have demonstrated remarkable efficacy in text classification tasks. Support vector machines (SVMs)[19-21] with appropriate kernel functions excel in high-dimensional feature spaces typical of text data. Their mathematical foundation in statistical learning theory provides good generalization even with limited training data. However, feature engineering remains crucial for optimal performance.

Ensemble methods like Random Forests[22, 23] and Gradient Boosting[24, 25] offer robust performance across diverse text classification tasks, handling feature interactions implicitly while providing feature importance metrics. These methods show resilience to overfitting when properly tuned, though they typically require more extensive preprocessing than deep learning approaches.

Naïve Bayes classifiers,[26-28] despite their simplifying independence assumption, remain surprisingly effective for text classification. Their probabilistic foundation allows for transparent decision-making and efficient training with minimal computational resources, making them particularly valuable for resource-constrained applications and as baseline models.

Unsupervised learning for text exploration

Clustering algorithms like K-means,[29] hierarchical clustering,[30] and DBSCAN[31] enable document organization without labeled data. When applied to appropriate text representations, these methods effectively identify thematic structures within corpora. However, selecting appropriate distance metrics and handling high dimensionality remain significant challenges.

Dimensionality reduction techniques, particularly principal component analysis (PCA)[32] and t-SNE,[33] facilitate visualization and exploration of text datasets by projecting high-dimensional representations into lower-dimensional spaces. These methods reveal global (PCA) and local (t-

SNE) structures in the data, though interpretation of the dimensions often requires domain expertise.

Deep learning approaches

Recurrent neural networks (RNNs),[34-40] particularly LSTM[40] and GRU[41] variants, revolutionized sequential text processing by capturing long-range dependencies and preserving word order information. These architectures demonstrated breakthrough performance in tasks requiring contextual understanding, including sentiment analysis and named entity recognition. However, their sequential nature limits parallelization and training efficiency.

Convolutional neural networks (CNNs)[42-46] adapted from computer vision have proven effective for text analysis, extracting local patterns through filters operating on word sequences. Their ability to identify significant n-grams regardless of position makes them particularly suitable for tasks like text classification and feature extraction, while offering parallelization advantages over RNNs.

The transformer[47-49] architecture represents the most significant paradigm shift in text analysis. By replacing recurrence with attention mechanisms, transformers process entire sequences simultaneously, capturing complex relationships between words regardless of their distance. This parallelization enables efficient training on massive corpora, resulting in models with unprecedented language understanding capabilities.

Pre-trained transformer models like BERT,[50, 51] GPT,[52] and T5[53] employ self-supervised learning on enormous text corpora, creating contextual representations that capture nuanced semantic and syntactic features. Fine-tuning these models on specific downstream tasks has established new state-of-the-art results across virtually all text analysis benchmarks, though at the cost of substantial computational requirements.

Challenges and future directions

Despite remarkable progress, machine learning for text analysis faces ongoing challenges. Interpretability remains difficult, particularly for deep learning approaches, though attention visualization and post-hoc explanation methods offer partial solutions. Bias and fairness concerns have grown more prominent as models increasingly influence real-world decisions, necessitating careful evaluation and mitigation strategies.

The computational and environmental costs of training large language models present sustainability challenges, driving research into more efficient architectures and training methods. Meanwhile, multilinguality and cross-cultural capabilities remain underdeveloped compared to English-language processing.

Machine learning approaches have transformed text analysis from a primarily rule-based discipline to one dominated by data-driven methods. The field continues to evolve rapidly, with hybrid approaches combining the interpretability of traditional methods with the representational power of deep learning. As models grow in capability and accessibility, the focus increasingly shifts toward responsible deployment that considers ethical implications alongside technical performance. The continued integration of linguistic knowledge with statistical learning promises further advances in machines' ability to understand, generate, and reason with human language.

Co-occurrence analysis

Co-occurrence methods[54-64] represent a fundamental approach in text data analysis, focusing on the statistical relationships between words that appear together within a defined context. These methods have become increasingly important in natural language processing (NLP), information retrieval, and computational linguistics.

Co-occurrence analysis is grounded in the distributional hypothesis, articulated by J.R. Firth's famous statement that "you shall know a word by the company it keeps." This principle suggests that words appearing in similar contexts tend to have related meanings. Building on this foundation, co-occurrence methods quantify the relationships between lexical items based on their patterns of appearance in textual data.

Term-document matrices

The most basic form of co-occurrence analysis utilizes term-document matrices,[59, 60] where rows represent terms, columns represent documents, and cells contain frequency counts or weighted values. While conceptually simple, these matrices provide the foundation for more sophisticated analyses but suffer from high dimensionality and sparsity issues.

Word-context matrices

Word-context matrices[60] capture relationships between target words and their surrounding contexts (typically neighboring words within a specified window). These matrices enable more nuanced analysis of semantic relationships by considering the immediate linguistic environment of words rather than just document-level co-occurrence.

Pointwise mutual information (PMI)

PMI[61, 62] measures the strength of association between co-occurring words by comparing observed co-occurrence frequencies with what would be expected if the words were distributed independently. The formula:

$$PMI(x,y) = \log(P(x,y)/(P(x)P(y)))$$

PMI effectively identifies meaningful word associations but can overemphasize rare co-occurrences.

Vector space models

Word2Vec[63] and GloVe[55, 64] methods transform words into dense vector representations based on distributional statistics of word co-occurrences. Their ability to capture semantic relationships through vector arithmetic operations has revolutionized numerous NLP applications. However, these models struggle with polysemy and require large training corpora.

Applications

Co-occurrence methods excel at identifying semantically similar words based on shared contexts, enabling thesaurus construction and automated synonym detection. Techniques like latent dirichlet allocation (LDA)[8] utilize co-occurrence statistics to identify latent topics in document collections, revealing thematic structures in large corpora. Co-occurrence statistics enhance document retrieval by expanding queries with related terms and improving relevance rankings through semantic matching. Statistical measures of co-occurrence strength help identify meaningful multi-word expressions and collocations that function as lexical units. Word co-

occurrence networks represent terms as nodes connected by edges weighted by co-occurrence strength, enabling the application of graph theory to text analysis and visualization.

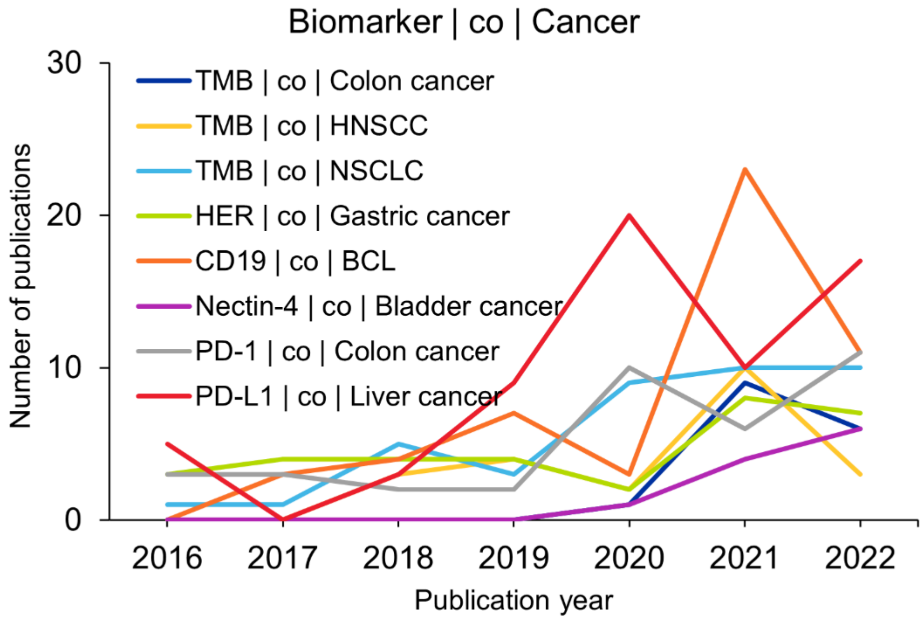

Figure S1. Time trends of a few chosen concept pairs across the category's biomarkers and cancer types. Data includes journal and patent publications related to immuno-oncology from the CAS Content Collection for the period 2016-2022.

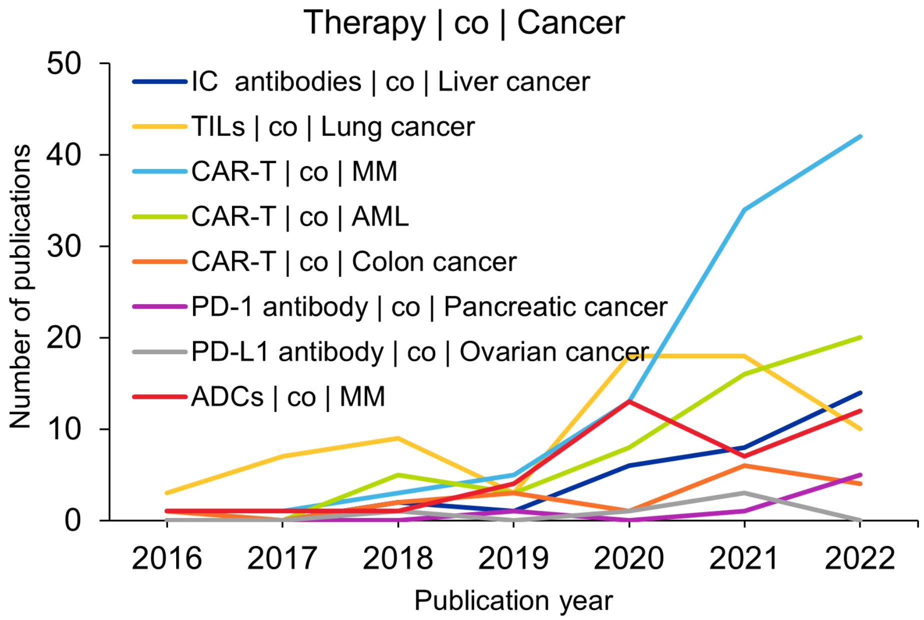

Figure S2. Time trends of a few chosen concept pairs across the category's therapy and cancer types. Data includes journal and patent publications related to immuno-oncology from the CAS Content Collection for the period 2016-2022.

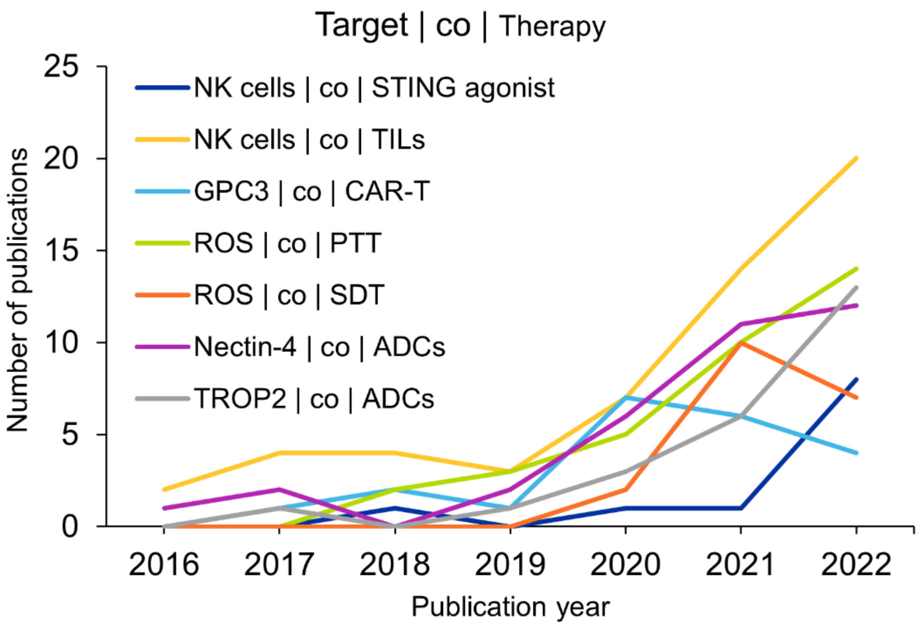

Figure S3. Time trends of a few chosen concept pairs across the category's therapeutic targets and therapy types. Data includes journal and patent publications related to immuno-oncology from the CAS Content Collection for the period 2016-2022.

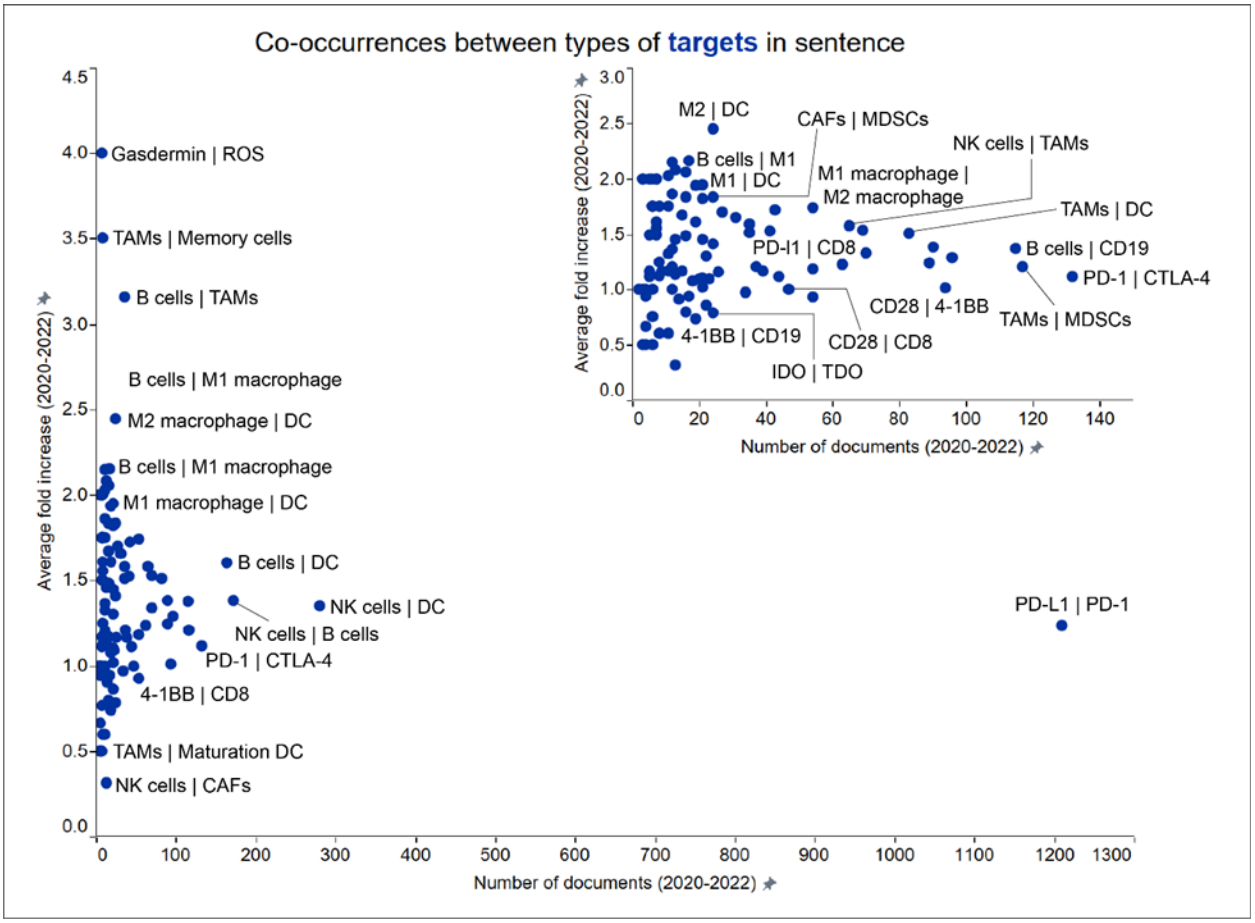

Figure S4. Co-occurrence analysis of emerging concepts in immuno-oncology focused on types of therapeutic targets. Concepts pairs co-occurred within a sentence in the title or abstract of publications. Labels outline certain noteworthy combinations exhibiting either high number of documents (right part of the graph) or high growth rate (upper left). Shown inset is a graph focused on data clustered in the lower left corner of the bigger graph.

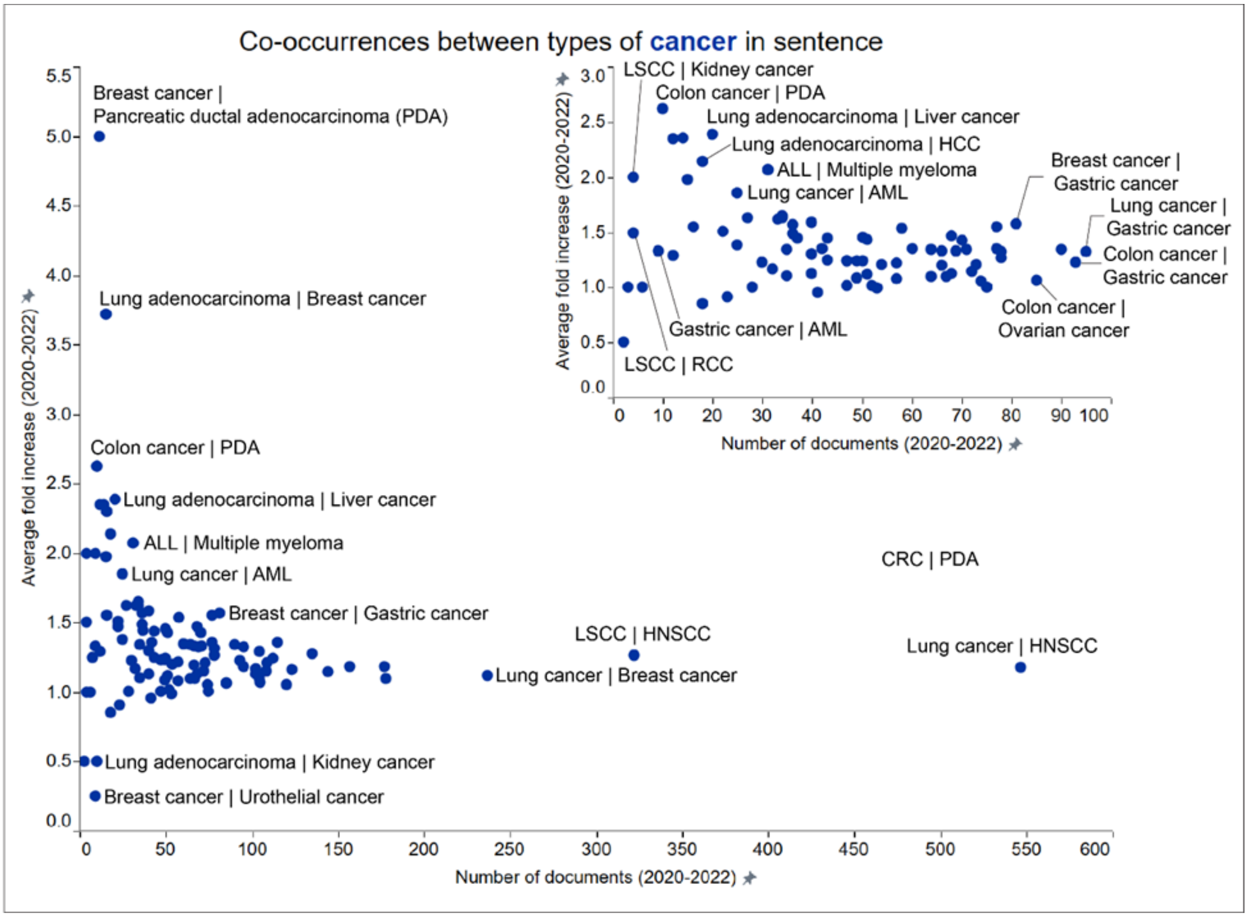

Figure S5. Co-occurrence analysis of emerging concepts in immuno-oncology focused on types of cancer. Concepts pairs co-occurred within a sentence in the title or abstract of publications. Labels outline certain noteworthy combinations exhibiting either high number of documents (right part of the graph) or high growth rate (upper left). Shown inset is a graph focused on data clustered in the lower left corner of the bigger graph.

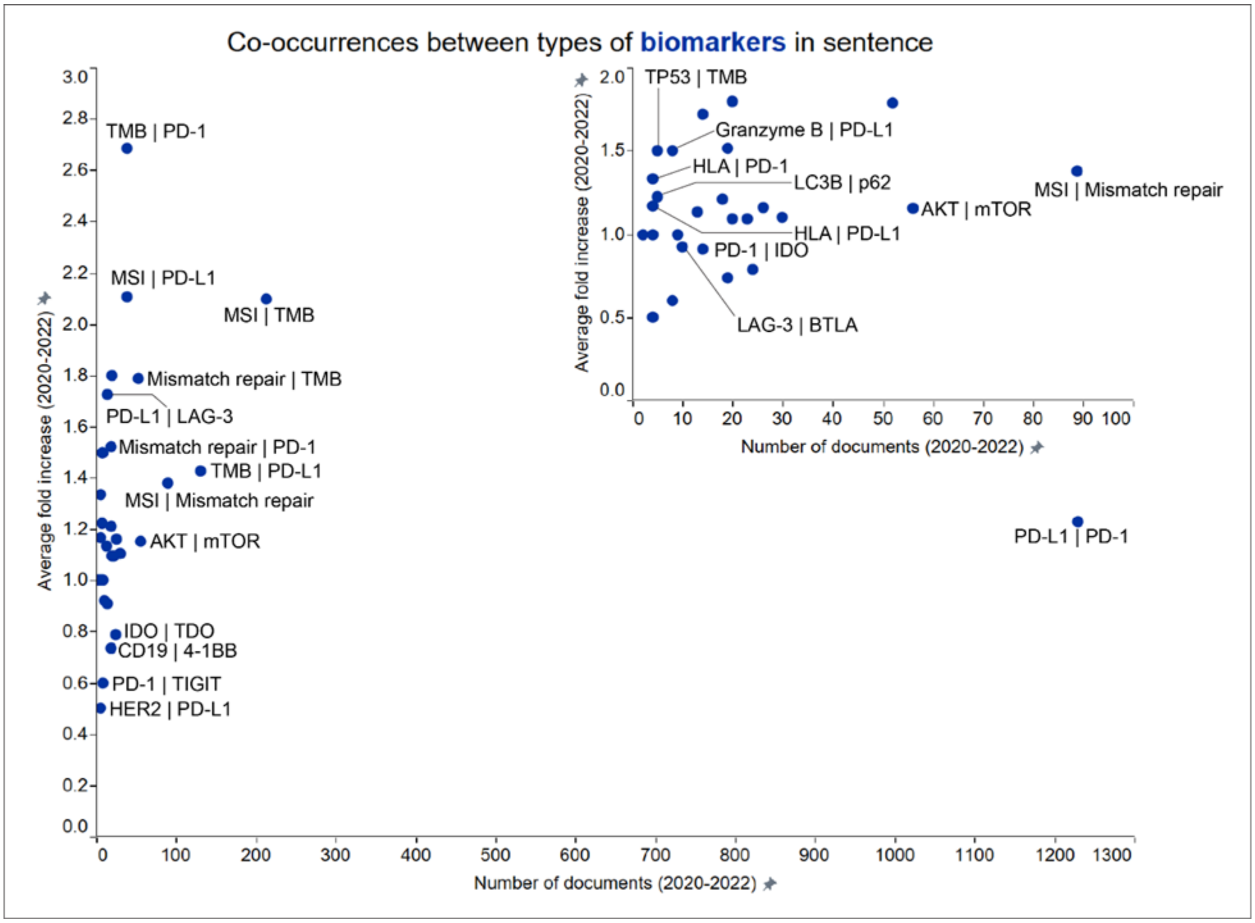

Figure S6. Co-occurrence analysis of emerging concepts in immuno-oncology focused on biomarkers. Concepts pairs co-occurred within a sentence in the title or abstract of publications. Labels outline certain noteworthy combinations exhibiting either high number of documents (right part of the graph) or high growth rate (upper left). Shown inset is a graph focused on data clustered in the lower left corner of the bigger graph.

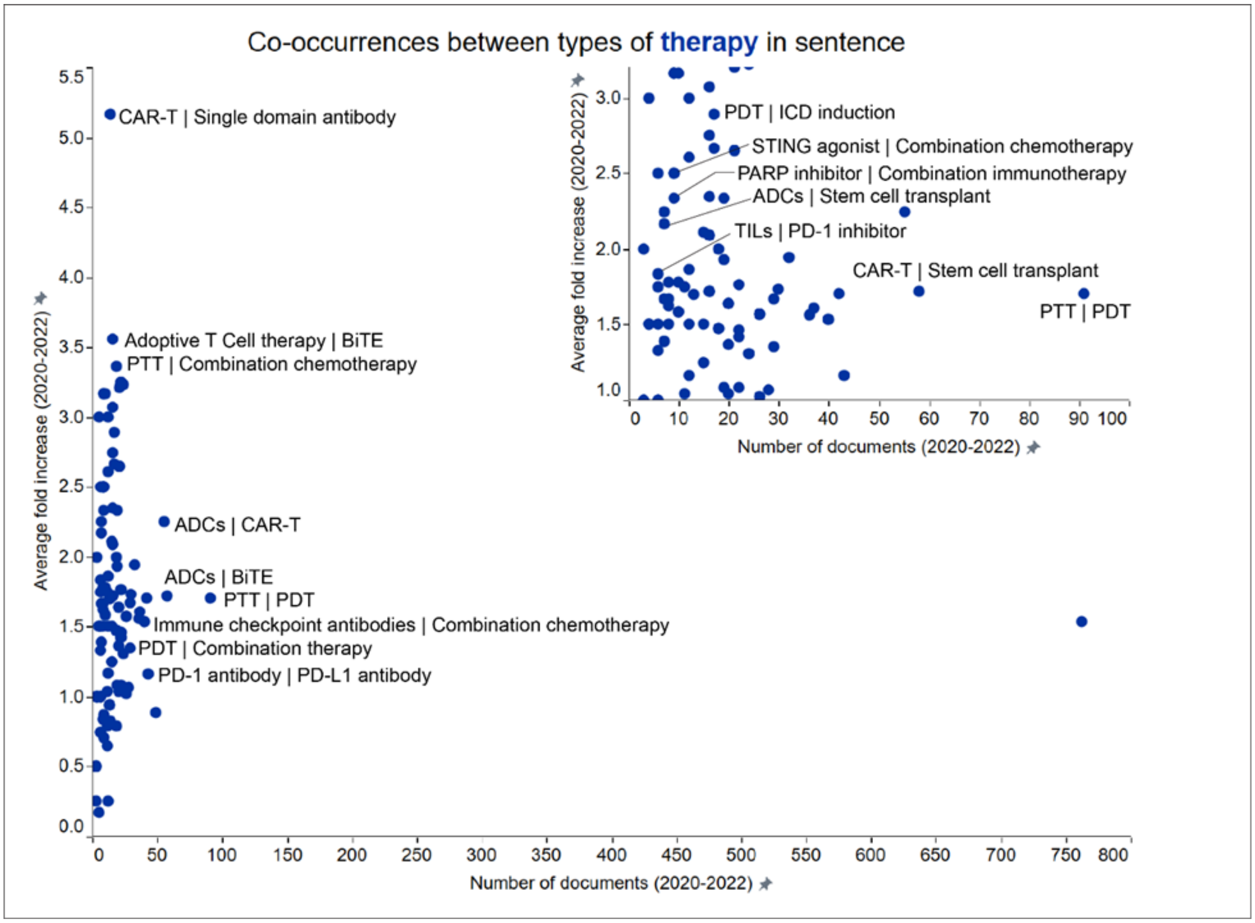

Figure S7. Co-occurrence analysis of emerging concepts in immuno-oncology focused on types of therapy. Concepts pairs co-occurred within a sentence in the title or abstract of publications. Labels outline certain noteworthy combinations exhibiting either high number of documents (right part of the graph) or high growth rate (upper left). Shown inset is a graph focused on data clustered in the lower left corner of the bigger graph.

References

(1) Iyer, K. A.; Ivanov, J.; Tenchov, R.; Ralhan, K.; Rodriguez, Y.; Sasso, J. M.; Scott, S.; Zhou, Q. A. Emerging targets and therapeutics in immuno-oncology: Insights from landscape analysis. *Journal of Medicinal Chemistry* **2024**, *67* (11), 8519-8544. DOI: 10.1021/acs.jmedchem.4c00568.
(2) Ivanov, J.; Lipkus, A.; Chen, H.; Aultman, C.; Iyer, K.; Tenchov, R.; Zhou, Q. [Pre-print] Emerging topics – bibliometric-based methodology. *ChemRxiv* **2023**. DOI: 10.26434/chemrxiv-2023-7d37m.
(3) Rose, S.; Engel, D.; Cramer, N.; Cowley, W. Automatic keyword extraction from individual documents. In *Text Mining: Applications and Theory*, Berry, M. W., Kogan, J. Eds.; John Wiley & Sons, Ltd, 2010; pp 1-20.
(4) TF-IDF. In *Encyclopedia of Machine Learning*, Sammut, C., Webb, G. I. Eds.; Springer, 2011; pp 986-987.
(5) Campos, R.; Mangaravite, V.; Pasquali, A.; Jatowt, A.; Jorge, A.; Nunes, C.; Jatowt, A. YAKE! Keyword extraction from single documents using multiple local features. *Information Sciences* **2020**, *509*, 257-289. DOI: 10.1016/j.ins.2019.09.013.
(6) Campos, R.; Mangaravite, V.; Pasquali, A.; Jorge, A. M.; Nunes, C.; Jatowt, A. A text feature based automatic keyword extraction method for single documents. In *Advances in Information Retrieval. ECIR 2018.* , G., P., B., P., L., A., A., H. Eds.; Lecture Notes in Computer Science, Vol. 10772; Springer, 2018.
(7) Campos, R.; Mangaravite, V.; Pasquali, A.; Jorge, A. M.; Nunes, C.; Jatowt, A. YAKE! Collection-independent automatic keyword extractor. In *Advances in Information Retrieval. ECIR 2018.*, G., P., B., P., L., A., A., H. Eds.; Lecture Notes in Computer Science, Vol. 10772; Springer, 2018.
(8) Blei, D. M.; Ng, A. Y.; Jordan., M. I. Latent dirichlet allocation. *Journal of Machine Learning Research* **2003**, *3*, 993-1022.
(9) Teh, Y. W.; Jordan, M. I.; Beal, M. J.; Blei, D. M. Hierarchical dirichlet processes. *Journal of the American Statistical Association* **2006**, *101* (476), 1566–1581. DOI: 10.1198/016214506000000302.
(10) Lee, D.; Seung, H. Learning the parts of objects by non-negative matrix factorization. *Nature* **1999**, *401*, 788–791. DOI: 10.1038/44565.
(11) Le, Q.; Mikolov, T. Distributed representations of sentences and documents. In *31st International Conference on Machine Learning*, Beijing, China, 2014; Vol. 32, pp 1188-1196.
(12) Lau, J. H.; Baldwin, T. An empirical evaluation of doc2vec with practical insights into document embedding generation. Berlin, Germany, August, 2016; Association for Computational Linguistics: pp 78-86. DOI: 10.18653/v1/W16-1609.
(13) Tan, S.; Cheng, X.; Wang, Y.; Xu, H. Adapting naive Bayes to domain adaptation for sentiment analysis. In Advances in Information Retrieval. ECIR 2009., 2009.
(14) Joyce, B.; Deng, J. Sentiment analysis using naive Bayes approach with weighted reviews - A case study. In *2019 IEEE Global Communications Conference (GLOBECOM)*, Waikoloa, HI, USA, 2019; pp 1-6. DOI: 10.1109/GLOBECOM38437.2019.9013588.
(15) Wang, F.; Zhang, J. L.; Li, Y.; Deng, K.; Liu, J. S. Bayesian text classification and summarization via a class-specified topic model. *Journal of Machine Learning Research* **2021**, *22* (89), 1-48.
(16) Rudolph, M.; Ruiz, F. J. R.; Mandt, S.; Blei, D. M. Exponential family embeddings. In *30th Conference on Neural Information Processing Systems (NIPS 2016)*, Barcelona, Spain, 2016.
(17) Baer, B. R.; Seto, S.; Wells, M. T. Exponential family word embeddings: An iterative approach for learning word vectors. In *32nd Conference on Neural Information Processing Systems (NIPS 2018)*, Montréal, Canada, 2018.
(18) Celikkanat, A.; Malliaros, F. D. Exponential family graph embeddings. In Proceedings of the AAAI Conference on Artificial Intelligence, 2020.
(19) Patro, A.; Patel, M.; Shukla, R.; Save, J. Real time news classification using machine learning. *IJAST* **2020**, *29* (9), 620-630.
(20) Kumar, R. R.; Reddy, M. B.; Praveen, P. Text classification performance analysis on machine learning. *International Journal of Advanced Science and Technology* **2019**, *28* (20), 691 – 697.
(21) Spirovski, K.; Stevanoska, E.; Kulakov, A.; Popeska, Z.; Velinov, G. Comparison of different model's performances in task of document classification. In WIMS '18: Proceedings of the 8th International Conference on Web Intelligence, Mining and Semantics, Novi Sad, Serbia; 2018.
(22) Islam, M. Z.; Liu, J.; Li, J.; Liu, L.; Kang, W. A semantics aware random forest for text classification. In CIKM '19: Proceedings of the 28th ACM International Conference on Information and Knowledge Management, 2019.

(23) Jalal, N.; Mehmood, A.; Choi, G. S.; Ashraf, I. A novel improved random forest for text classification using feature ranking and optimal number of trees. *Journal of King Saud University - Computer and Information Sciences* **2022**, *34* (6), 2733-2742. DOI: 10.1016/j.jksuci.2022.03.012.
(24) Alzamzami, F.; Hoda, M.; Saddik, A. E. Light gradient boosting machine for general sentiment classification on short texts: A comparative evaluation. *IEEE Access* **2020**, *8*, 101840-101858. DOI: 10.1109/ACCESS.2020.2997330.
(25) Singh, S.; Krishnan, D.; Sehgal, P.; Sharma, H.; Surani, T.; Singh, J. Gradient boosting approach for sentiment analysis for job recommendation and candidate profiling. In 2022 IEEE Bombay Section Signature Conference (IBSSC), Mumbai, India; 2022.
(26) Zhang, W.; Gao, F. An improvement to naive Bayes for text nlassification. *Procedia Engineering* **2011**, *15*, 2160-2164. DOI: 10.1016/j.proeng.2011.08.404.
(27) Kaur, R.; Singh, H. Naive Bayes: A machine learning based text classifier. In 8th International Conference on Advancements in Engineering and Technology, (ICAET-2020), BGIET, Sangrur; 2020.
(28) Singh, G.; Upadhyay, M.; Sharma, U.; Hussain, S.; Jain, U. Text classification system using naïve Bayes algorithm. *International Journal of Research Publication and Reviews* **2021**, *2* (7), 544-554.
(29) Sinaga, K. P.; Yang, M.-S. Unsupervised K-means clustering algorithm. *IEEE Access* **2020**, *8*, 80716-80727. DOI: 10.1109/ACCESS.2020.2988796.
(30) Dhillon, I. S.; Mallela, S.; Kumar, R. Enhanced word clustering for hierarchical text classification. In *KDD '02: Proceedings of the eighth ACM SIGKDD international conference on Knowledge discovery and data mining*, New York, NY, USA, 2002; pp 191-200. DOI: 10.1145/775047.775076.
(31) Andriyani, F.; Puspitarani, Y. Performance comparison of K-Means and DBScan algorithms for text clustering product reviews. *Sinkron : Jurnal Dan Penelitian Teknik Informatika* **2022**, *6* (3), 944-949. DOI: 10.33395/sinkron.v7i3.11569.
(32) Greenacre, M.; Groenen, P. J. F.; Hastie, T.; D'Enza, A. I.; Markos, A.; Tuzhilina, E. Principal component analysis. *Nature Reviews Methods Primers* **2022**, *2*, 100. DOI: 10.1038/s43586-022-00184-w.
(33) van der Maaten, L.; Hinton, G. Visualizing data using t-SNE. *Journal of Machine Learning Research* **2008**, *9*, 2579-2605.
(34) Luo, L.; Yang, Z.; Yang, P.; Zhang, Y.; Wang, L.; Lin, H.; Wang, J. An attention-based BiLSTM-CRF approach to document-level chemical named entity recognition. *Bioinformatics* **2018**, *34* (8), 1381-1388. DOI: 10.1093/bioinformatics/btx761 From NLM Medline.
(35) Arevian, G. Recurrent neural networks for robust real-world text classification. In *IEEE/WIC/ACM International Conference on Web Intelligence (WI'07)*, Fremont, CA, USA, 2007; pp 326-329. DOI: 10.1109/WI.2007.126.
(36) Thomas, M.; C.A, L. Sentimental analysis using recurrent neural network. *International Journal of Engineering and Technology* **2018**, *7* (2.27), 88-92. DOI: 10.14419/ijet.v7i2.27.12635.
(37) Meenakshi; Akkash, S. R.; Kumar, S. D.; Rajasekar, D.; Sharan Prasad, S. Sentiment analysis using recurrent neural networks. *International Research Journal of Engineering and Technology (IRJET)* **2020**, *7* (8), 204-207.
(38) Mienye, I. D.; Swart, T. G.; Obaido, G. Recurrent neural networks: A comprehensive review of architectures, variants, and applications. *Information* **2024**, *15* (9), 517. DOI: 10.3390/info15090517.
(39) Patel, A.; Tiwari, A. K. Sentiment analysis by using recurrent neural network. In *Proceedings of 2nd International Conference on Advanced Computing and Software Engineering (ICACSE) 2019*, 2019. DOI: 10.2139/ssrn.3349572.
(40) Kurniasari, L.; Setyanto, A. Sentiment analysis using recurrent neural network-LSTM in Bahasa Indonesia. *Journal of Engineering Science and Technology* **2020**, *15* (5), 3242 - 3256.
(41) Eswaraiah, P.; Syed, H. A hybrid deep learning GRU based approach for text classification using word embedding. *EAI Endorsed Transactions on Internet of Things* **2023**, *10*. DOI: 10.4108/eetiot.4590.
(42) Roy, P. K.; Kumar, A. Convolutional neural network for text: A stepwise working guidance. In *Proceedings of the Yukthi 2021- The International Conference on Emerging Trends in Engineering*, GEC Kozhikode, Kerala, India, 2021.
(43) Jacovi, A.; Sar Shalom, O.; Goldberg, Y. Understanding convolutional neural networks for text classification. Brussels, Belgium, November, 2018; Association for Computational Linguistics: pp 56-65. DOI: 10.18653/v1/W18-5408.
(44) Ge, J.; Lin , S.; Fang, Y. A text classification algorithm based on topic model and convolutional neural network. *Journal of Physics: Conference Series* **2021**, *1748*, 032036. DOI: 10.1088/1742-6596/1748/3/032036.

(45) Qiu, M.; Zhang, Y.; Ma, T.; Wu, Q.; Jin, F. Convolutional-neural-network-based multilabel text classification for automatic discrimination of legal documents. *Sensors and Materials* **2020**, *32* (8), 2659–2672. DOI: 10.18494/SAM.2020.2794.
(46) Fesseha, A.; Xiong, S.; Emiru, E. D.; Diallo, M.; Dahou, A. Text classification based on convolutional neural networks and word embedding for low-resource languages: Tigrinya. *Information* **2021**, *12* (2), 52. DOI: 10.3390/info12020052.
(47) Phuong, M.; Hutter, M. [Pre-print] Formal algorithms for transformers. *arXiv* **2022**, *2207.09238*.
(48) Durairaj, A. K.; Chinnalagu, A. Transformer based contextual model for sentiment analysis of customer reviews: A fine-tuned BERT. *International Journal of Advanced Computer Science and Applications(IJACSA)* **2021**, *12* (11). DOI: 10.14569/IJACSA.2021.0121153.
(49) Krishnan, A. [Pre-print] Exploring machine learning and transformer-based approaches for deceptive text classification: A comparative analysis. *arXiv* **2023**, *2308.05476*.
(50) Grootendorst, M. [Pre-print] BERTopic: Neural topic modeling with a class-based TF-IDF procedure. *arXiv* **2022**, *2203.05794*.
(51) Devlin, J.; Chang, M.-W.; Lee, K.; Toutanova, K. BERT: Pre-training of deep bidirectional transformers for language understanding. Minneapolis, Minnesota, June, 2019; Association for Computational Linguistics: pp 4171-4186. DOI: 10.18653/v1/N19-1423.
(52) Yenduri, G.; Ramalingam, M.; Selvi, G. C.; Supriya, Y.; Srivastava, G.; Maddikunta, P. K. R.; Raj, G. D.; Jhaveri, R. H.; Prabadevi, B.; Wang, W.; et al. GPT (Generative pre-trained transformer)— A comprehensive review on enabling technologies, potential applications, emerging challenges, and future directions. *IEEE Access* **2024**, *12*, 54608-54649. DOI: 10.1109/ACCESS.2024.3389497.
(53) Xue, L.; Constant, N.; Roberts, A.; Kale, M.; Al-Rfou, R.; Siddhant, A.; Barua, A.; Raffel, C. mT5: A massively multilingual pre-trained text-to-text transformer. Online, June, 2021; Association for Computational Linguistics: pp 483-498. DOI: 10.18653/v1/2021.naacl-main.41.
(54) Kang, C.; Choi, J. Impact of co-occurrence on factual knowledge of large language models. Singapore, December, 2023; Association for Computational Linguistics: pp 7721-7735. DOI: 10.18653/v1/2023.findings-emnlp.518.
(55) Pennington, J.; Socher, R.; Manning, C. GloVe: Global vectors for word representation. Doha, Qatar, October, 2014; Association for Computational Linguistics: pp 1532-1543. DOI: 10.3115/v1/D14-1162.
(56) Lin, W.; Wu, X.; Wang, Z.; Wan, X.; Li, H. Topic network analysis based on co-occurrence time series clustering. *Mathematics* **2022**, *10* (16), 2846. DOI: 10.3390/math10162846.
(57) Petit, Q.; Li, C.; Emad, N. An efficient and scalable approach to build co-occurrence matrix for DNN's embedding layer. *ICS '24: Proceedings of the 38th ACM International Conference on Supercomputing* **2024**, 286-297. DOI: 10.1145/3650200.3656629.
(58) Saravanan, K.; Choudhury, M.; Udupa, R.; Kumaran, A. An empirical study of the occurrence and co-occurrence of named entities in natural language corpora. Istanbul, Turkey, May, 2012; European Language Resources Association (ELRA): pp 3118-3125.
(59) Leydesdorff, L.; Vaughan, L. Co-occurrence matrices and their applications in information science: Extending ACA to the Web environment. *Journal of the American Society for Information Science and Technology* **2006**, *57* (12), 1616-1628. DOI: 10.1002/asi.20335.
(60) White, H. D.; McCain, K. W. Visualizing a discipline: An author co-citation analysis of information science, 1972–1995. *Journal of the American Society for Information Science* **1998**, *49* (4), 327-355. DOI: 10.1002/(SICI)1097-4571(19980401)49:4%3C327::AID-ASI4%3E3.0.CO;2-4.
(61) Bouma, G. Normalized (pointwise) mutual information in collocation extraction. *Proceedings of the Biennial GSCL Conference 2009* **2009**.
(62) Rijcken, E.; Zervanou, K.; Spruit, M.; Scheepers, F.; Kaymak, U. Effect of calculating pointwise mutual information using a fuzzy sliding window in topic modeling. In *IEEE International Conference on Fuzzy Systems (FUZZ)*, 2023; pp 1-6. DOI: 10.1109/FUZZ52849.2023.10309675.
(63) Mikolov, T.; Sutskever, I.; Chen, K.; Corrado, G.; Dean, J. Distributed representations of words and phrases and their compositionality. In *NIPS'13: Proceedings of the 27th International Conference on Neural Information Processing Systems*, 2013; Curran Associates Inc.: Vol. 2, pp 3111 - 3119.
(64) Shi, T.; Liu, Z. [Pre-print] Linking GloVe with word2vec. *arXiv* **2014**, *1411.5595*.
